# From computational screening to *in vitro* validation: exploring antimicrobial peptides against *Pseudomonas aeruginosa*

**DOI:** 10.3389/fmicb.2026.1796090

**Published:** 2026-06-11

**Authors:** Debolina Chatterjee, Indranil Biswas, Sumit Mittal, Sreyoshi Routh, Venkatraman Manickam, Kazi Tanvir, G. Karunanidhi, Atul Godha, Karthikeyan Sivashanmugam

**Affiliations:** 1 School of Biosciences and Technology, Vellore Institute of Technology, Vellore, India; 2 Department of Microbiology, Molecular Genetics and Immunology, University of Kansas Medical Center, Kansas City, KS, United States; 3 School of Advanced Sciences and Languages (SASL), VIT Bhopal University, Sehore, Madhya Pradesh, India; 4 School of Advanced Sciences, Vellore Institute of Technology, Vellore, India; 5 Anthem Biosciences, Bangalore, India

**Keywords:** antimicrobial peptides, antimicrobial resistance, MexB efflux pump, molecular dynamics simulation, multidrug resistance, Omiganan, *Pseudomonas aeruginosa*, SAAP-148

## Abstract

Advanced therapeutics such as antimicrobial peptides (AMPs) represent promising alternatives for combating antimicrobial resistance due to their diverse mechanisms of action, including membrane disruption and interference with bacterial resistance systems. *Pseudomonas aeruginosa* is recognized as a priority pathogen because of its intrinsic and acquired resistance to multiple antibiotics, largely mediated by the overexpression of efflux pump systems such as MexAB-OprM. In this study, an extensive *in-silico* screening approach was employed to identify AMPs capable of interacting with the MexB efflux pump protein. Molecular docking and molecular dynamics simulations identified Omiganan, SAAP-148, and their engineered variants as promising candidates with strong binding affinity toward MexB. Selected peptides were synthesized and evaluated *in vitro* against clinical and reference strains of *P. aeruginosa*. The peptides exhibited minimum inhibitory concentrations ranging from 8 to 64 μg/mL. Flow cytometric analysis using propidium iodide uptake and scanning electron microscopy confirmed membrane disruption in peptide-treated cells. Cytotoxicity assays demonstrated comparatively low toxicity for Omiganan and SAAP-148_SM1 in HEK293 and HepG2 cell lines. Collectively, these findings highlight the potential of rationally designed antimicrobial peptides as modulators of efflux-mediated resistance. The identified peptides provide a foundation for further optimization and *in vivo* evaluation as potential therapeutic agents or antibiotic adjuvants against multidrug-resistant *P. aeruginosa* infections.

## Graphical abstract

**Figure:**
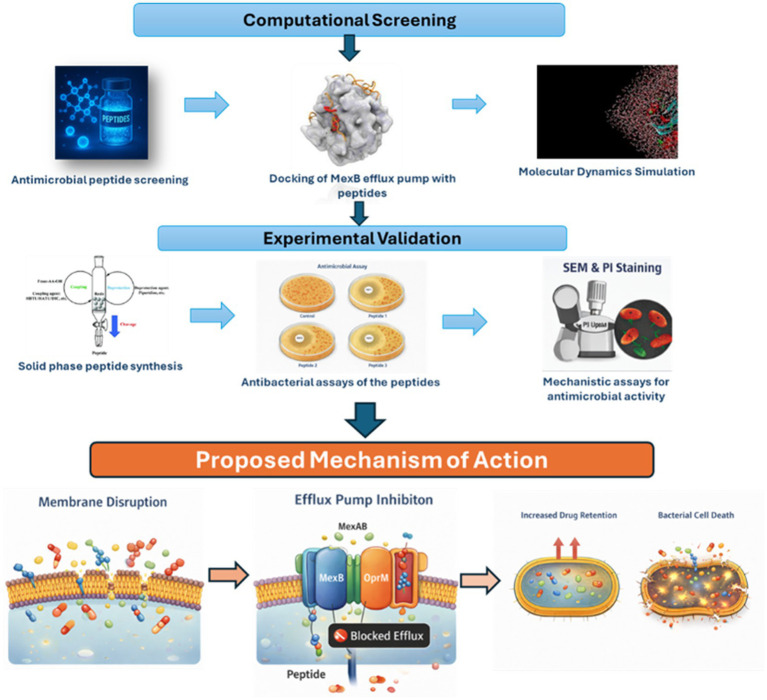

## 1 Introduction

*Pseudomonas aeruginosa* is a versatile and opportunistic pathogen, notorious for its intrinsic and acquired resistance to multiple classes of antibiotics, contributing to the rising threat of antimicrobial resistance (AMR) (Pang et al., 2019). The WHO has identified carbapenem-resistant *P. aeruginosa* as a priority pathogen for antibiotic research and development (Losito et al., 2022). Notably, the COVID-19 pandemic has significantly enhanced the prevalence of carbapenem-resistant *P. aeruginosa* infections, due to tenuous hospital stays and increased use of broad-spectrum antibiotics (Bongiovanni and Barda, 2023). Antibiotic overuse and a dearth of surveillance data from tertiary care facilities are contributing factors to the ongoing AMR epidemic (Salam et al., 2023).

The key factors contributing to the emergence of antimicrobial resistance in *P. aeruginosa* include the overexpression of genes responsible for modifying antibiotics, such as β-lactamases (Pang et al., 2019), point mutations in drug target sites, and the upregulation of efflux pump proteins that facilitate the extrusion of antimicrobial agents (Barnabas et al., 2022). Of these, efflux pump-mediated resistance warrants particular attention, as it plays a central role in reducing the bioavailability of antibiotics within bacterial cells, thereby limiting the effectiveness of many commonly used drugs (Akinduti et al., 2023). Efflux pumps play a crucial role in bacterial evolution and environmental adaptation, primarily due to their ability to extrude toxins, antibiotics, and antimicrobials; secondly, by regulating the expression of genes associated with bacterial virulence, and thirdly, contributing to biofilm formation, facilitating bacterial adherence to various surfaces and providing protective structures that enhance survival and resistance to antibiotics (Avakh et al., 2023; Christaki et al., 2020).

The RND group of efflux pumps in *P. aeruginosa,* such as the MexAB-OprM, MexCD-OprJ, and MexEF-OprN systems, actively pump out a wide range of antibiotics, including β-lactams, aminoglycosides, and fluoroquinolones from inside to outside of the bacterial cell, often leading to a reduced intracellular concentration of the drug below the therapeutic threshold (Akinduti et al., 2023; Elfadadny et al., 2024).

Efflux pump gene expression is controlled by both transcriptional and post-transcriptional factors. Transcriptional factors act as repressors and post-transcriptional factors act as activators of gene expression (Du et al., 2018). In the presence of antibiotics, post-transcriptional factors activate efflux pump gene expression leading to resistance to a particular drug (Grkovic et al., 2001). However, the resistance mechanism can be intrinsic and reversible. A combination of colistin and tobramycin can downregulate efflux pump genes and enhance lethality against *Pseudomonas* (Cianciulli Sesso et al., 2021). The focal point of several studies is to either suppress these efflux pumps or eliminate the efflux pump genes to control resistance to the mutations that confer AMR.

MexAB-OprM, belonging to the resistance nodulation division class of the efflux pumps in *P. aeruginosa* plays a significant role in resistance to beta-lactam antibiotics (Theel et al., 2024). With increased drug concentration, the MexB protein changes the conformational pattern to eject the active drug across the periplasm and outer membrane through the MexA and OprM. Overexpression of MexAB-OprM is often due to the mutations in *nalB* repressor gene, which is a repressor of the MexAB-OprM operon, making the cells resistant to antimicrobial agents (Canè et al., 2023) and also contributing to biofilm formation (Akinduti et al., 2023; Papadopoulos et al., 2008).

Efflux pump inhibitors, especially antimicrobial peptides are mostly naturally occurring peptides derived from living systems (Mookherjee et al., 2020). These peptides exhibit broad-spectrum activities such as disruption of the bacterial cell, modulation of the efflux pump activities and cell lysis. They were found to produce enhanced synergistic activity in combination with antibiotics against *Pseudomonas* (Avakh et al., 2023; Askoura et al., 2011). For instance, esculentin-1a [Esc(1–21)-1c], a frog-skin AMP, showed a synergistic effect with erythromycin, chloramphenicol, or tetracycline in inhibiting the growth of *P. aeruginosa* PAO1 without being cytotoxic to human cells (Canè et al., 2023). In another study, Trp-containing peptides exhibited synergistic activities with ceftazidime and piperacillin at low concentrations significantly decreasing β-lactamase activity and the expression of efflux pump genes (Shang et al., 2021).

The goal of this study was to develop a promising and targeted therapeutic approach for combating emerging multidrug-resistant (MDR) strains of *P. aeruginosa* and addressing the AMR challenge with more definitive and strategic interventions. In the study, an *in silico* approach was employed to screen the antimicrobial peptides deposited in the public database to find a suitable peptide against the MexB efflux pump inhibitor in a much more cost-effective and time-efficient manner. Also, *in vitro*, the selected peptides were synthesized and studied against various MDR strains of *P. aeruginosa* to understand the mechanism of action of these AMPs and their combinatorial activity with the commonly used antibiotics.

## 2 Materials and methods

### 2.1 *In silico* approach

#### 2.1.1 Retrieval of antimicrobial peptides from public databases

The antimicrobial peptides were retrieved from the Antimicrobial Peptide Database (APD3), the Data Repository of Antimicrobial Peptides (DRAMP), and the Database of Antimicrobial Activity and Structure of Peptides (DBAASP) under five categories: linear, cationic, cysteine-rich, neutral, and alpha-helical linear. AMPs were selected based on their chain length (20–30 amino acids), net positive charge, and defense activity against Gram-negative bacteria. The selected peptides were then used as parent AMPs for screening against the MexB efflux pump protein of *Pseudomonas aeruginosa.*

#### 2.1.2 Screening of antimicrobial peptides against the MexB efflux pump of *Pseudomonas aeruginosa*

The MexB structure was retrieved from the PDB database using the ID 2 V50. The structure was modified to remove the ligands and optimized using the GalaxyRefine server to remove the clash scores, poor rotamers, and percentage Ramachandran outliers (Lee et al., 2019). HPEP DOCK 2.0 web server was used to screen the AMPs against the MexB efflux pump of *Pseudomonas aeruginosa* (Zhou et al., 2018). Multiple peptide sequences in FASTA format of length 15–20 amino acids were used as the “Peptide Input,” and the MexB protein structure was uploaded in pdb format as the “Receptor Input.” The virtual screening method was chosen, in which each docked peptide is ranked using binding scores, and only the top-scored binding mode is produced as output for each peptide.

#### 2.1.3 Generation of mutant peptide library

The peptides with higher binding scores were identified through virtual screening using the HPEP Dock 2.0 server. The top 10 peptides were ranked based on their binding energy values and further selected for generating the mutant peptide library. Studies have demonstrated that the anti-microbial efficacy of the peptides could be enhanced by replacing the amino acids in the template sequence or on the parent peptide sequence (Duval et al., 2009). The substitution of the amino acids on the template peptide sequence was carried out in a 5′ → 3′ direction following the amino acid walking method by replacing minimally significant amino acids such as asparagine (N), tryptophan (W), valine (V), leucine (L), methionine (M), phenylalanine (F), histidine (H), tyrosine (Y), glutamic acid (E), and aspartic acid (D) with lysine (K), proline (P), cysteine (C), isoleucine (I) (Kumar et al., 2019). Further, mutated peptides were screened against the MexB efflux pump protein using the HPEP Dock 2.0 server against the MexB efflux pump protein of *Pseudomonas aeruginosa* (Basu et al., 2022).

#### 2.1.4 Evaluation of the physicochemical properties, antigenicity, and toxicity of the peptides

The physicochemical properties such as hydrophobicity, net positive charge (> + 2), isoelectric point, molecular weight, and relative stability, of the parent peptides along with their mutated variants were analyzed using the HLP server (Sharma et al., 2014). The cell-penetrating ability of the selected parent peptide and its variants was studied using a machine learning model-based MLCPP 2.0 server (Manavalan and Patra, 2022). The antigenic peptide tool was used to predict the antigenic properties of the peptides (es/Tools/antigenic).^1^ A support Vector Machine (SVM) based approach was used for classifying the AMPS as toxic or non-toxic with the help of the ToxinPred server (Gupta et al., 2013; Gupta et al., 2015). The correlation of the physicochemical properties, antigenicity, and toxicity of the parent AMPs with the docking profiles was carried out to shortlist the peptides further for the study process.

#### 2.1.5 Docking of the MexB protein with the antimicrobial peptides

The PEP-FOLD 4.0 server was used to determine the 3D structures of the parent peptides and their mutants (Rey et al., 2023). The models were validated using the MolProbity program, which identified the stereochemical characteristics of the residues that fell within the Ramachandran Plot’s permitted and prohibited areas (Davis et al., 2007). Protein-peptide docking was carried out using the ClusPro 2.0 server (Desta et al., 2020; Vajda et al., 2017). The low binding energy profiles of the peptide-protein complexes were generated through semi-derivative Monte-Carlo simulations in ClusPro 2.0. The binding interactions of the least binding energy complexes were studied using the DIMPLOT of the LigPlot+ package. Upon evaluation of the docking scores and physicochemical, antigenicity, CPP, and toxicity parameters, shortlisted peptide-protein complexes were subjected to molecular dynamics simulation.

#### 2.1.6 Molecular dynamics simulation of the shortlisted protein-peptide complexes

The interactions of short-listed peptides (6 peptides) with MexB in a solvated environment were investigated using all-atom classical molecular dynamics (MD) simulations. The protein-peptide complexes, obtained from docking, were used as the initial geometry for these simulations. Each complex was embedded in a lipid bilayer made up of 200 POPE and 150 POPG lipids, followed by solvation using explicit water molecules, and was charge neutralized by adding the requisite number of Na + and Cl- ions to achieve a physiological ionic strength of 0.15 M. The resulting systems were subjected to energy minimization using the steepest descent algorithm. Each system was equilibrated in two phases: 1 ns of NVT simulation followed by 1 ns of NPT simulation, both at 303.15 K. Subsequently, 100 ns production runs were conducted for each system under periodic boundary conditions. The temperature and pressure were maintained at 303.15 K using the Nose-Hover thermostat and coupling algorithm, and at 1 atm using the Parrinello-Rahman barostat, respectively. A time step of 2 fs was used in these simulations, while the Particle Mesh Ewald method was employed to account for long-range electrostatic interactions. All the simulations were performed using GROMACS 2023.3, while the CHARMM 36 m force field was used for all molecules and ions.

The binding affinity of each protein-peptide complex was estimated using the Molecular Mechanics Poisson-Boltzmann Surface Area (MM-PBSA) approach using the MMPBSA 1.6.3 tool. Further, the trajectories of each system were analyzed using GROMACS and VMD tools. Secondary structure analysis was carried out using DSSP to monitor changes in the peptide and protein’s structural elements during the simulations. The H-bond was quantified based on geometric criteria, with a donor-acceptor distance cutoff of 3.5 Å and a donor-hydrogen-acceptor angle cutoff of 30°.

### 2.2 *In vitro* analysis

#### 2.2.1 Materials

*Pseudomonas aeruginosa* (ATCC 27853) strain and clinical isolates were collected from CMC Hospital, Vellore, and Anthem Biosciences, Bangalore. The bacterial isolates were grown on Luria-Bertani broth (LB broth) and maintained on LB agar plates at 37° C. HEK-293 (human embryonic kidney) cells and HEPG2 (hepatoblastoma) cell lines were procured from NCCS, Pune, India. The cells were cultured in DMEM supplemented with 10% FBS (Fetal bovine serum) and 10,000 IU ampicillin and streptomycin in a humidified condition at 37 °C and 5% CO_2_. All the culture media and antibiotics were procured from HiMedia, India. Based on molecular dynamics simulation studies, understanding their dynamics and structural stability, four antimicrobial peptides were synthesized using the standard solid-phase synthesis method with 9-fluorenyl methoxycarbonyl (Fmoc) protection. The peptides were purified by reverse-phase HPLC, and the purity of the peptides was analyzed by LC–MS.

#### 2.2.2 Circular dichroism spectroscopy of the peptides

**Figure 1:**
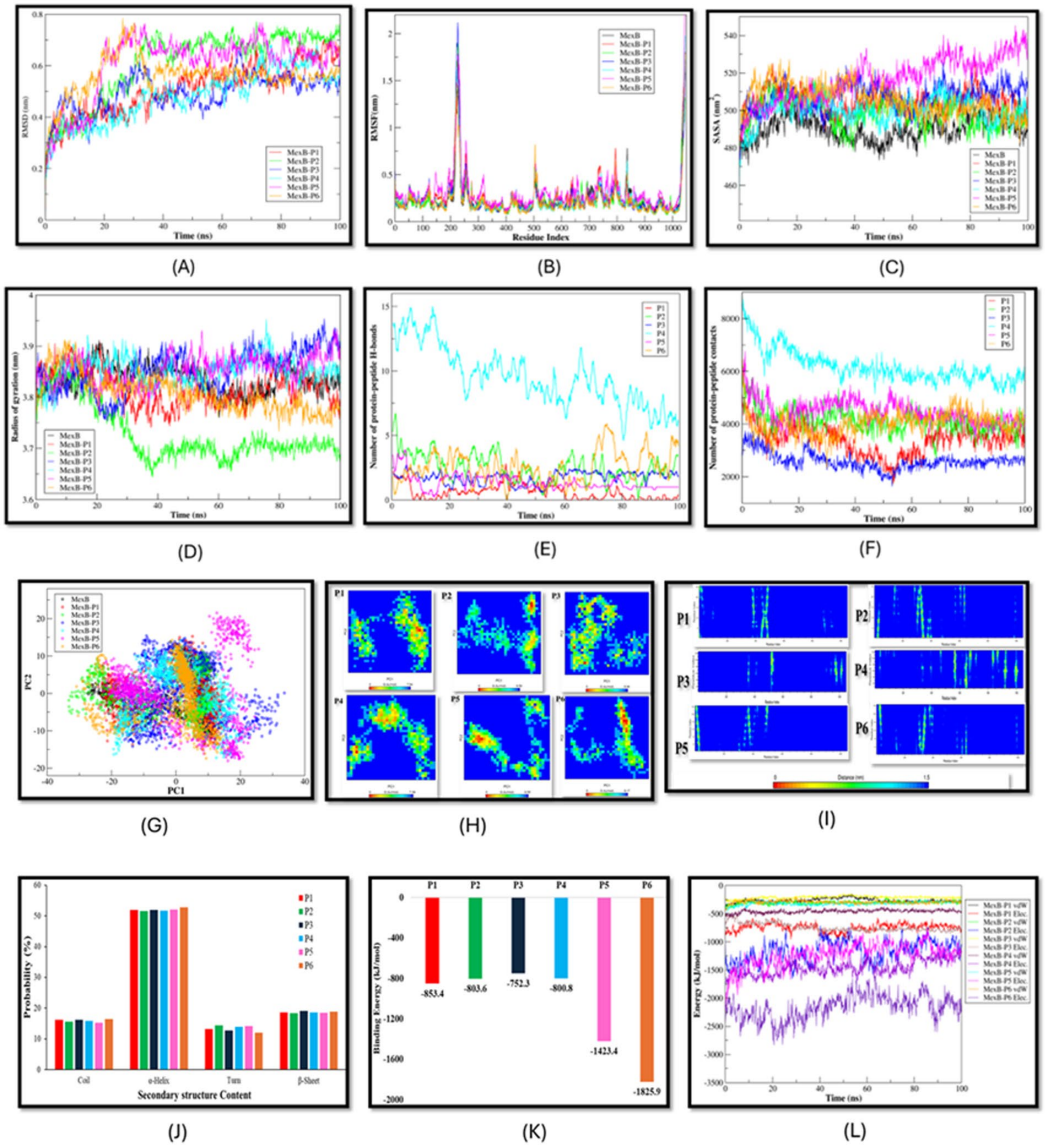
Molecular dynamics simulation of the MexB-peptide complexes. **(A)** Root mean square deviation of the protein-peptide complexes. **(B)** Root mean square fluctuations of the protein-peptide complexes. **(C)** Solvent access surface area (SASA) of the protein- peptide complexes. **(D)** The radius of gyration of the protein-peptide complexes. **(E)** Hydrogen bonding pattern of the protein-peptide complexes. **(F)** Protein-peptide contacts’ map. **(G)** Principal component analysis. **(H)** Free energy landscapes for the MexB-peptide complexes. **(I)** Distance-based contact maps between MexB protein and peptides. **(J)** Average propensity percentage of each secondary structure motif across the 6 peptides. **(K)** Binding energy calculated from MM-PBSA calculations. **(L)** Contribution of vdW and electrostatics interactions to binding energy calculated from MM-PBSA calculations. P1 stands for Apo C1, P2 indicates Apo C1_M34, P3 represents Omiganan, P4 stands for Omiganan_M14, P5 signifies SAAP-148, and P6 indicates SAAP-148_M57 peptides.

Secondary structure details of the synthesized AMPs were analyzed by far-UV circular dichroism (CD) spectroscopy with ThermoFisher Scientific Model No. 2821 PLC-230 LP at a wavelength range between 190 and 500 nm using a path length of 1 mm. The spectra of peptides were measured at a concentration of 1 mg/mL. Spectra were baseline corrected by subtracting a blank spectrum containing only buffer and expressed as Wavelength vs. milidegrees (mDeg). Figure 1 illustrates the CD data of the peptides along with the control (PBS), with wavelength in the X-axis and the mDegrees of the peptides in the Y-axis. BeStSel (Beta Structure Selection) webserver^2^ was used to analyze the spectral data to obtain the secondary structural details.

#### 2.2.3 Antibiotic susceptibility pattern of the bacterial isolates

The strains were tested for the antibiotic susceptibility pattern following the CLSI (Clinical and Laboratory Standards Institute) guidelines 2024 against commonly used antibiotics like nalidixic acid, tobramycin, amoxicillin, amikacin, ciprofloxacin, imipenem, meropenem and colistin following Kirby-Bauer’s disc diffusion method.

#### 2.2.4 Minimum inhibitory concentration of the peptides

Synthesized AMPs and the commonly used fluoroquinolone drug (Ciprofloxacin) and FDA-approved antimicrobial peptide (Colistin) were further tested for their MIC values against *Pseudomonas aeruginosa* ATCC 278536 and 6 multidrug-resistant *Pseudomonas aeruginosa* clinical isolates. To ensure the reliability and reproducibility of the experimental observations, appropriate positive and negative controls were included in all assays. In the antimicrobial susceptibility and MIC assays, untreated bacterial cultures served as the growth control, while sterile media without bacterial inoculum served as the sterility control. Ciprofloxacin and colistin were used as reference antimicrobial agents to validate the activity of the synthesized antimicrobial peptides. All the cultures were grown in nutrient broth until they reached the mid-exponential growth phase. A total of 50 μL of cation-adjusted Muller-Hinton broth (MHB) was added to the wells. Peptides along with the reference antibiotics were added at a concentration ranging from 128 to 1 μg/mL and incubated for 24 h. On incubation, the plate was read at A_630_ nm to determine the MIC of the peptides.

#### 2.2.5 Minimum biofilm inhibitory concentration of the AMPs

Minimum biofilm inhibitory concentration was determined according to Alenazy (2023). *Pseudomonas aeruginosa* strains were grown in LB broth for 24 h at 37 °C, 180 rpm. The overnight bacterial suspension was diluted in LB broth to a 1:100 dilution. A total of 100 μL of the bacterial suspension was added to the 96-well plate and incubated for 24 h at 37 °C. Following 24 h incubation, the planktonic cells are removed by washing the plate with sterile distilled water three to four times. After washing, 50 μL of sterile LB broth was added to the wells, and 50 μL of the peptides with 0.5 MIC and 0.25 MIC was added. The plates were incubated overnight followed by washing with distilled water 3–4 times. A total of 125 μL of 0.1% crystal violet solution was added to each well and air-dried for 1 h. To solubilize the crystal violet, 70% ethanol was used. The test samples and controls were measured at 575 nm. The wells containing bacterial cultures without peptide treatment were used as positive biofilm controls, whereas wells containing only media served as negative controls.

#### 2.2.6 Combination assay of the AMPs with antibiotics

The microbroth checkerboard assay was utilized as a two-dimensional method to evaluate the combinations of antimicrobial agents (Alenazy, 2023). Serial two-fold dilutions of the antimicrobial solutions (A and B) were prepared. Agent A represents the antimicrobial peptide and agent B represents the antibiotic (ciprofloxacin and colistin) with the concentration ranging from two times MIC to at least 1/8 to 1/16 times. A total of 50 μL of CAMHB was dispensed along the first row and column. A total of 50 μL of Agent A was added serially diluted along the rows whereas 50 μL of Agent B was serially diluted along the columns. Growth control and sterility control were maintained with 100 μL of CAMHB. MIC for the antimicrobials (A and B) was recorded in combination and FICI (Fractional Inhibitory Concentration Index) was calculated using the formula:

$$\begin{array}{l} \text{FICI}=(\begin{array}{l} \mathrm{MIC}\;\text{of Agent}\;A\;\text{in combination}/\\ \mathrm{MIC}\;\text{of Agent}\;A\;\text{alone} \end{array})+\\ \left(\begin{array}{l} \mathrm{MIC}\;\text{of Agent}\;B\;\text{in combination}/\\ \mathrm{MIC}\;\text{of Agent}\;B\;\text{alone} \end{array}\right) \end{array}$$ (1)

If the FICI value is less than 0.5, it indicates a synergistic interaction between the antibiotics and the antimicrobial peptides. A FICI value between 0.5 and 1 suggests an additive effect, while a FICI value greater than 1 indicates an antagonistic mechanism of action between the antibiotic and the peptide.

#### 2.2.7 Propidium iodide uptake assay

*Pseudomonas aeruginosa* ATCC 27853 was cultured in the mid-log phase in MHB media and diluted in MHB media to adjust the optical density at 600 nm to 0.1. The bacterial cultures were treated with 2X and 3X MIC of the AMPs in MHB solution and incubated in a shaker at 37 °C for 1 h. Following incubation, 20 μL of propidium iodide (1 mg/mL) was added to each of the peptide-treated bacterial cultures and incubated for 1 h under dark conditions. Untreated bacterial cells were used to establish the baseline fluorescence corresponding to intact cell membranes. Increased fluorescence in peptide-treated samples was compared against this control to validate membrane permeabilization. PI fluorescence was measured at excitation and emission wavelengths of 580 and 620 nm, respectively (Kwon et al., 2019).

#### 2.2.8 Ethidium bromide cartwheel assay

The Ethidium Bromide Cartwheel Assay (EtBrCwA) is a qualitative and practical approach for estimating the effectiveness of the antimicrobial peptides against control and multi-drug-resistant bacterial strains (Tian et al., 2024; Costa et al., 2013). The overnight bacterial culture of *Pseudomonas aeruginosa* ATCC27853 was swabbed onto the Tryptic Soy agar (TSA) plates containing EtBr at concentrations of 0.5 and 1 mg/L, respectively, and the antimicrobial peptides at 0.5X MIC and the positive controls which are known for efflux pump inhibitors like phenylalanine-arginine β-naphthylamide (PaβN) and clove oil were included as positive controls to confirm the inhibition of efflux pump activity. After an incubation at 37 °C for 16–18 h, EtBr-agar plates are visualized under a UV light source.

### 2.3 Safety evaluation of the peptides

#### 2.3.1 Hemolysis inhibition activity of the AMPs

Blood from a healthy individual was collected and then subjected to centrifugation at 3,000 rpm for 5 min using tubes treated with heparin, using the protocol (Rodríguez et al., 2014). Subsequently, it was rinsed thrice using an equivalent volume of saline solution (0.9% NaCl). Post-centrifugation, the volume of the blood was recorded, and the blood was then diluted to form a 10% volume/volume suspension in a phosphate-buffered saline solution with a pH of 7.4, consisting of 10 millimoles of sodium phosphate. Precisely, 500 μL of RBC suspension and 500 μL of AMPs at variable concentrations of 15, 50, and 100 μg/mL were mixed with 2.95 mL phosphate-buffered solution (pH 7.4). To perform the heat-induced hemolysis, the mixture was incubated in a water bath at 54 °C for 20 min followed by centrifugation at 2500 g for 3 min. The absorbance was measured at 540 nm. For the control, the phosphate-buffered solution was used. The percentage inhibition of hemolysis was calculated using the following equation using the formula:

$$\text{Percentage inhibition of hemolysis}=100^{*}(1-A2/\mathrm{A1})$$ (2)

where A1 = absorbance of the control; A2 = absorbance of test sample mixture.

#### 2.3.2 Cytotoxic effect of the antimicrobial peptides

MTT was used to assess cell viability. HEK-293 and HEPG2 cells were seeded in a 96-well plate with a density of 1 × 10^4^ cells and incubated overnight at 37 °C and 5% CO_2_. Post incubation, the cells were treated with varying concentrations of antimicrobial peptides and incubated for 24 h. Following incubation, the media was removed from the wells, and MTT (5 mg/mL) was added to each well and incubated in the dark for 4 h at 37 °C. The MTT solution was removed after 4 h, and 100 μL of DMSO was added to solubilize the formazon crystal. Absorbance was taken at 570 nm with the help of a microplate reader (Bio-Tek, United States), and cell viability was analyzed. Untreated HEK-293 and HepG2 cells served as negative controls to determine baseline cell viability, while cells treated with increasing concentrations of peptides were compared against this reference. All experiments were performed in independent replicates to ensure reproducibility, and the results were interpreted relative to the corresponding controls to validate the antimicrobial and efflux pump inhibitory effects of the peptides. Statistical analyses were carried out using the GraphPad Prism software, and a *p* < 0.05 was considered statistically significant.

## 3 Results

### 3.1 Retrieval of the MexB protein and initial screening of antimicrobial peptides from the AMP libraries

The MexB protein (PDB ID 2 V50) from *Pseudomonas aeruginosa* was obtained from the RCSB Protein Data Bank. The structure was visualized using PyMOL to analyze the interactions between the amino acid residues and the ligand. To prepare the target protein for subsequent procedures, heteroatoms including water and the ligand were removed. The hydrogen atoms and Gasteiger charges were assigned using Discovery Studio version 21.1.1. APD3, DRAMP, and DBAASP databases were used for the retrieval of the antimicrobial peptides that are less than 30 amino acids long. In total, 730 AMPS from ADP3, 708 from DRAMP, and 870 AMP sequences from the DBAASP database were retrieved. Supplementary file S1 contains the list of the AMPs that were retrieved. The peptides obtained were screened against the MexB efflux pump protein using the HPEP Dock 2.0 server. Based on the binding energy values, the top 10 peptides were selected for further study (Table 1).

**Table 1:** A list of the top 10 anti-microbial peptides as shortlisted arranged based on HPEP dock 2.0 binding score.

Peptide name	Peptide sequences	HPEP dock score
ecPis-2	FFFHIIKGLFHAGRMIHGLV	−333.496
SAAP-148	LKRVWKRVFKLLKRYWRQLKKPVR	−331.23
oc_45	MVVVGIVVGVVGIILLAFVAPLTWGFIGVM	−320.284
Chicken CATH-2	RFGRFLRKIRRFRPKVTITIQGSARFG	−315.624
Omiganan	ILRWPWWPWRRK	−307.878
APOC1	FSTKTRNWFSEHFKKVKEKLKDTFA	−305.184
Pleurocidin	GWGSFFKKAAHVGKHVGKAALTHYL	−304.767
PcAst-1a	SNGYRPAYRPAYRPSYRPGK	−302.581
Competence stimulating peptide	SGSLSTFFRLFNRSFTQALGK	−300.167
git_8	MVRILHSFLITKCIGSMRTFMPYCTCLAFR	−299.908

### 3.2 Assessment of the physicochemical properties, antigenicity, and toxicity of the AMP library

The top 10 shortlisted peptides and their mutants were assessed for their physicochemical properties such as molecular weight, charge, pH, half-life, cell-penetrating permeability, hydrophobicity, and stability in the intestinal environment which were listed in Supplementary file S3. Omiganan, Mutacin, SAAP-148, Pleurocidin, PcAst 1-a, and Competence Stimulating Peptide and their corresponding mutants showed normal to high relative stability, whereas ApoC1, Chicken Cathelicidin-2, Ec-Pis2, and some of their variants showed low stability. The peptides’ relative stability ranged from 2.5 to 4, their molecular weight ranged from 2,000 to 3,000 g/mol, and their isoelectric point ranged from 6 to 7. The hydrophobicity of SAAP-148 was 16.148 kJ/mol initially, but it increased by almost 20 kJ/mol in its mutant variants. The hydrophobicity values of Omiganan, ApoC1, and Chicken Cathelicidin-2 parent peptide were 13.588 kJ/mol and 10.989 kJ/mol, respectively. In the case of mutants, the hydrophobicity ranged between 8 and 13 kJ/mol. Pleurocidin, PcAst-1, Competence Stimulating Peptide, and Mutacin II exhibited lower hydrophobicity values. Based on the cell-penetrating permeability (CPP) of the AMPS, they were categorized into CPP and Non-CPP. SAAP-148 and its mutants were CPP Chicken Cathelicidin-2 parent peptides are non-CPP, whereas its mutants were CPP, Omiganan, and its mutants were CPP, Apo C1 parent peptide and its mutants (ApoC1_M1, ApoC1_M5, ApoC1_M10, ApoC1_M11, ApoC1_M33-46) were CPP, Pleurocidin and its mutants were non-CPP, PcAst 1A and its mutants (Pc_M1-3, Pc_M7, Pc_M10-12, Pc_17,18, Pc_21) were CPP, Competence Stimulating Peptides, Mutacin II and its mutants were non-CPP. None of the peptides (either parent/ mutant) was found to be toxic and the antigenicity probability of the peptides was between 1.02 and 1.08. The 3D structures of the AMPs were predicted, and the model qualities were determined for further docking studies.

### 3.3 Protein–peptide interactions of the shortlisted peptides

The ClusPro 2.0 docking server was used for docking the AMP library against the MexB protein of *Pseudomonas aeruginosa* which followed the Piper rigid body algorithm allowing 70,000 rotations in three-dimensional space with a radius of 1.0 Å. CHARMM energy minimization was used for shortlisting the top 1,000 energy conformations. Approximately, 600 peptides (template + mutant) were docked against the protein. The top 8 peptides, along with their mutants, are listed in Supplementary Table 2 with their screening scores and Cluspro Docking scores. Among the top 10 parent peptides, Omiganan (−1288.8 kcal/mol), ec-Pis 2 (−1237.3 kcal/mol), APOC1 (−1236.6 kcal/mol), and chicken cathelicidin 2 (−1195.6 kcal/mol), SAAP-148 (−1127.4 kcal/mol), Mutacin (−1047.1 kcal/mol) showed the highest binding affinities. In contrast, the highest negative binding energies reported by the top 10 peptide variants could be listed as the following: Mutacin_M2(−1438.8 kcal/mol), SAAP_M48 (−1417.2 kcal/mol), ecPis2_M31 (−1390.7 kcal/mol), ApoC1_M34 (−1327.6 kcal/mol), Omiganan_M14(−1271.3 kcal/mol). Most of the mutant peptides showed higher binding affinities towards the target protein than their parent peptides.

### 3.4 Molecular dynamics simulation of the peptide–protein complexes

**Figure 2:**
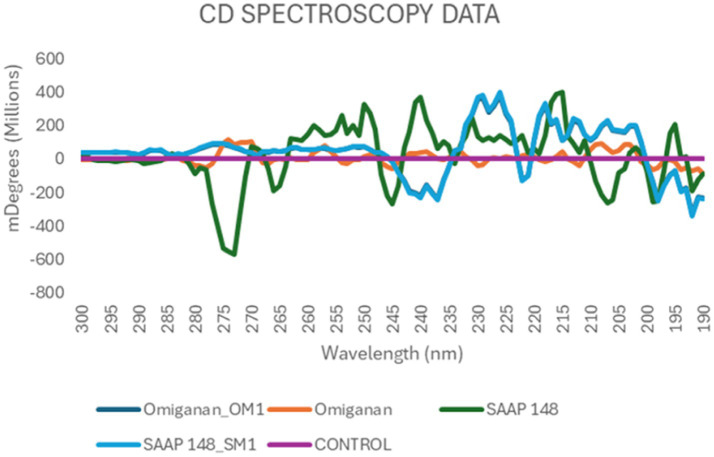
Secondary structure details of the synthesized antimicrobial peptides.

The Root Mean Square Deviation (RMSD) was calculated for the MexB protein in its unbound form and its complex with various antimicrobial peptides (P1-P6) over the 100 ns MD simulations (Figure 2A). In the unbound form, MexB showed the lowest average RMSD of 0.48 nm. The MexB-Omiganan (P3) and MexB-Omiganan_M14 (P4) complexes also showed comparable RMSD values of 0.49 and 0.48 nm, respectively. The MexB-ApoC1_M34 (P2) complex showed an RMSD of 0.61 nm, suggesting a significant conformational change from its initial conformation and lower stability compared to MexB alone.

In the Root Mean Square Fluctuation (RMSF) analysis (Figure 2B), MexB alone showed a pronounced peak around residue 200, where the RMSF reaches approximately 2 nm. When MexB was complexed with Apo C1 (P1), the overall RMSF profile remained similar to that of the unbound protein, but with a slight increase in flexibility observed at the N-terminal region (residues 0–100). The MexB-Apo C1_M34 (P2) complex also showed high flexibility around residue 200 and a slightly higher RMSF value as compared to MexB alone and the MexB-ApoC1 complex. Omiganan (P3) appears to have a stabilizing effect on MexB and the Omiganan_M14 variant (P4) introduces additional flexibility, especially in the C-terminal region (residues 800–1,000). The binding of SAAP-148 (P5) results in a noticeable increase in flexibility at residue 200 and in the intermediate region. The SAAP-148_M57/SAAP-148_SM1 (P6) amplifies this effect, leading to the highest overall RMSF values observed among all complexes.

The SASA (Solvent Access Surface Area) analysis (Figure 2C) showed that while all systems undergo conformational changes, the MexB-SAAP-148 (P5) complex showed the highest SASA values, while MexB and MexB-ApoC1_M34(P2) maintained relatively lower SASA values (Table 2).

**Table 2:** List of protein peptide interaction scores as obtained from Hpep Dock 2.0 server and ClusPro server.

Peptide name	Peptide sequence	HPEP dock score	ClusPro score
Chicken CATH-2	RFGRFLRKIRRFRPKVTITIQGSARFG	−315.624	−1195.6
CC_11	RFGRCCRKIRRFRPKVTITIQGSARFG	−238.805	−1,100
CC_26	RFGRFPRKIRRFRPKVTITIQGSARFG	−287.947	−1292.7
CC_29	RCGRFLRKIRRFRPKVTITIQGSARFG	−258.483	−1211.5
CC_17	RFGRFLRKIRRFRPKITITIQGSARIG	−258.164	−1,190
**OMIGANAN**	**ILRWPWWPWRRK**	−307.878	**−1288.8**
OM_14	IIRIPWWPWRRK	−264.469	−1271.3
OM_22	ILRWPWWPKRRK	−255.382	−1237.3
OM_34	ILRIPWWPWRRK	−261.86	−1256.7
OM_29	ILRCPWWPWRRK	−262.376	−1204.1
APOC1			
**AP_01**	**FSTKTRNWFSEHFKKVKEKLKDTFA**	−305.184	−1236.6
AP_18	FSTKTRNWFSCCFKKVKEKLKDTFA	−221.698	−1254.7
AP_29	FSTKTRNWFSIIFKKVKEKLKDTFA	−271.369	−1366.6
AP_34	FSTKTRNWFSEHFKKVKEKIKITFA	−265.919	−1327.6
AP_61	FSTKTRNWFSECFKKVKEKLKDTFA	−247.367	−1348.4
Mutacin			
**MU_1**	**NRWWQGVVPTVSYECRMNSWQHVFTCC**	**−296.927**	**−1047.1**
MU_2	KRKWQGVVPTVSYECRMNSWQHVFTCC	−238.753	−1438.8
MU_9	NRWWQGVVPTVSYKCRKNSWQHVFTCC	−265.195	−1,434
MU_36	NRWWQGVVPTVSYECRMCSCQHVFTCC	−248.048	−1499.4
MU_49	NRWWQGVVPTVSYECRMISIQHVFTCC	−254.958	−1,494
Ec-Pis			
**ec_1-original**	**FFFHIIKGLFHAGRMIHGLV**	−333.496	**−1237.3**
ec_31	FFFHIIKGLFHAGRMIHGCC	−278.823	−1390.7
ec_56	FFFPIIKGLFHAGRMIHGLV	−267.038	−1372.6
ec_76	FIFHIIKGLFHAGRMIHGLV	−265.542	−1358.4
ec_78	FFFIIIKGLFHAGRMIHGLV	−277.949	−1,366
SAAP 148			
**SP_1**	**LKRVWKRVFKLLKRYWRQLKKPVR**	−331.23	**−1127.4**
SP_48	PKRVWKRVFKLLKRYWRQLKKPVR	−274.351	−1417.2
SP_49	LKRVPKRVFKLLKRYWRQLKKPVR	−249.043	−1299.1
SP_57	CKRVWKRVFKLLKRYWRQLKKPVR	−259.894	−1435.2
SP_58	LKRVCKRVFKLLKRYWRQLKKPVR	−269.342	−1,359
Pn-Cath2			
**PN_ori**	EGCNILCLLKRKVKAVKNVVKNVVKSVVG	−234.642	−1330.3
PM_1	**P**GCNILCLLKRKVKAVKNVVKNVVKSVVG	−261.434	−1206.4
PM_2	EGCNILCLLKRKVKAVKNVVK**CC**VKSVVG	−302.271	−1140.7
PM_4	EGCNILCLLKRKVKAVKNVVKNV**C**KS**C**VG	−263.377	−1094.4
PM_3	EGCNILCLLKRKVKAVKNVVKNVVKSV**K**G	−298.89	−1084.7
Pcst-1			
PC_ori	**SNGYRPAYRPAYRPSYRPGK**	−241.511	−808.3
PCM1	SNG**K**RPAYRPAYRPSYRPGK	−230.246	−901.4
PCM2	SNG**C**RPAYRPAYRPS**K**RPGK	−239.659	−860.6
PCM3	SNGCRPAYRPAYRPS**I**RPGK	−232.624	−914.9
PCM4	S**K**G**K**RPAYRPAYRPSYRPGK	−247.498	−835.8

The radius of gyration data (Figure 2D) shows that MexB and its peptide complexes are relatively stable over the simulation time, with values fluctuating around 3.8–3.9 nm. The MexB- ApoC1_M34 (P2) complex showed a slightly lower value, 3.7 nm. The individual peptides display a wider range of compactness, with values ranging from approximately 0.8–1.2 nm.

The plot (Figure 2E) of the number of Hydrogen bonds (H-bonds) formed between the protein and the peptides showed that peptide P4 results in the highest number of H-bonds, ranging from 10 to 15. P2 showed a moderate and stable interaction, with an average value of 5 H-bonds, whereas peptides P1, P3, and P5 consistently exhibited low numbers of H-bonds.

These findings were further supported by the number of contacts between the protein and peptides (Figure 2F), which showed that P4 has the highest number of contacts, stabilizing between 6,000 and 7,000 contacts. Peptides P5 and P6 displayed a slightly lower number of contacts, fluctuating between 4,000 and 6,000 contacts.

The free energy landscapes plotted against the first two principal components (PC1 and PC2) displayed distinct energy minima and higher barriers, suggesting multiple stable states (Figure 2G). Peptides P1, P2, and P3 have complex landscapes with several smaller basins, indicating higher conformational variability, whereas peptides P4, P5, and P6 exhibit larger, more centralized basins, suggesting fewer but more stable conformations.

The PCA plot (Figure 2H) showed that the two principal components, PC1 and PC2, capture significant variance in the data, with substantial overlap among the systems. MexB-P5 and MexB-P6 appear more isolated, indicating potential outliers or regions of higher variance.

The distance-based contact maps revealed consistent and variable interaction patterns between the protein and peptide across P1-P6 (Figure 2I). Dense interactions were observed primarily around residue indices 300–500 and 800–900 in P1. P2 showed a more dispersed interaction pattern. P3 depicts fewer and more specific interactions. P4 presented a sparser contact map. P5 mirrors P1 interaction regions but with more scattered contacts. P6 resembled P1 with spread-out interactions.

The bar graph of the average propensity of secondary structure motifs showed (Figure 2J) that *α*-helix has the highest propensity (50–55%), followed by β-sheets (20–25%), with coils and turns being less prominent.

The MM-PBSA calculations (Figure 2K) revealed that peptides P1 to P6 exhibit binding energies of varying levels against MexB. MexB-P6 complex showed the highest binding affinity with a binding energy of −1825.866 kJ/mol, followed by MexB-P5 at −1423.421 kJ/mol. MexB-P1, MexB-P2, and MexB-P4 have lower binding affinities with energies of −853.353 kJ/mol, −803.601 kJ/mol, and −800.785 kJ/mol, respectively. MexB-P3 has the lowest binding affinity, with an energy of −752.299 kJ/mol.

### 3.5 Antibiotic susceptibility pattern of the bacterial strains

The bacterial strains were tested for antibiotic susceptibility pattern (AST) using Kirby-Bauer’s disc diffusion method. PA4, KP579, and PT1700 strains were found to be multidrug resistant with intermediate susceptibility to colistin, whereas the other strains were resistant to almost all the groups of drugs used under the category of beta-lactamases, aminoglycosides, fluoroquinolone, carbapenems, and antimicrobial peptide, Colistin. Table 3 shows the AST pattern following the CLSI guidelines 2024 of the *P. aeruginosa* isolates.

**Table 3:** Antibiotic susceptibility pattern of the clinical isolates and screening isolate of *Pseudomonas aeruginosa.*

Sl No.	Strain name	Antibiotic susceptibility pattern through Kirby Bauer’s disc diffusion method								
		Nalidixic acid	Tobramycin	Piperacillin	Amoxicillin	Meropenem	Imipenem	Ciprofloxacin	Amikacin	Colistin
1.	PA 1 (K1I499)	R	S	R	R	R	S	R	R	S
2.	PA 2 (VI798)	S	R	R	R	S	S	I	R	S
3.	PA3 (V2857)	S	R	R	R	R	R	R	S	S
4.	PA 4 (KSP509)	I	R	R	I	R	R	R	R	S
5.	PA 5 (KFF92)	S	S	R	I	S	R	R	R	S
6.	PT1700	R	R	R	R	R	R	R	R	I
7.	KP579	R	R	R	R	R	R	R	R	I
8.	ATCC 27853	S	R	S	S	S	S	S	I	S

R, Resistant; S, Sensitive; I, Intermediate.

### 3.6 Secondary structural details of the AMPs

The CD spectral data were analyzed using the BestSel web server, which enabled the prediction of the secondary structure of the synthesized peptides. The analysis revealed that all four peptides predominantly exhibited anti-parallel β-sheet structures, with turns as the second most abundant secondary structure element. Specifically, the predicted anti-parallel β-sheet content was 43.2% for Omiganan, 43.4% for Omiganan_OM1, 32.3% for SAAP-148, and 42.6% for SAAP-148_SM1. Additionally, the predicted turn content was 15.2% for Omiganan, 13.6% for Omiganan_OM1, 22.5% for SAAP-148, and 13.5% for SAAP-148_SM1. These findings underscore the prominence of anti-parallel β-sheets in the secondary structures of the peptides, with variations in the turn content across the different peptides.

### 3.7 Minimum inhibitory concentration of the antimicrobial peptides and antibiotics

A total of 4 antimicrobial peptides were shortlisted for solid-phase synthesis. The peptides are synthesized with >90% purity. The minimum inhibitory concentration of the AMPs was tested to evaluate their effectiveness against the various drug-resistant clinical isolates of *Pseudomonas aeruginosa* and strain ATCC 27853. Table 4 lists the MIC of the peptides, the commonly used fluoroquinolone, Ciprofloxacin, and the antimicrobial peptide colistin.

**Table 4:** Minimum Inhibitory concentration of the antimicrobial peptides and antibiotics against the various strains of *Pseudomonas aeruginosa.*

Strain name	Omiganan (μg/mL)	Omiganan_OM1 (μg/mL)	SAAP 148 (μg/mL)	SAAP 148_SM1 (μg/mL)	Ciprofloxacin (μg/mL)	Colistin (μg/mL)
**ATCC 27853**	32	16	8	16	16	4
PA1 (K1I499)	64	64	64	64	64	8
PA2 (VI798)	16	64	64	64	64	8
PA3 (V2857)	32	64	64	32	16	8
PA4 (KSP509)	32	64	64	64	64	8
PA5 (KFF92)	64	64	64	32	64	4
PT1700	64	32	16	32	>128	16
KP579	64	32	8	16	>128	16

### 3.8 Minimum biofilm inhibitory concentration of the AMPs

The AMPs’ biofilm inhibitory ability was tested on 5 biofilm-forming strains of *Pseudomonas aeruginosa,* such as ATCC 27853, PA1 (K1I499), PA4 (KSP509), PT1700, and KP579. SAAP-148, SAAP-148_SM1, Omiganan, and Omiganan_OM1 were able to inhibit the biofilm of the above-mentioned strains in concentrations ranging from 32 to 64 μg/ML. Table 5 lists the minimum biofilm inhibitory concentration of the AMPs.

**Table 5:** The minimum biofilm inhibitory concentration of the AMPs against various strains of *Pseudomonas aeruginosa*.

Strain Id	Minimum biofilm inhibitory concentration of the peptides			
	Omiganan (μg/mL)	Omiganan_OM1 (μg/mL)	SAAP 148 (μg/mL)	SAAP 148_SM1 (μg/mL)
ATCC 27853	32	64	64	32
PA1	64	32	64	32
PA4	32	64	32	32
PT1700	64	32	32	32
KP579	32	32	32	32

### 3.9 Combinatorial inhibitory effect of AMPs with antibiotics

**Figure 3:**
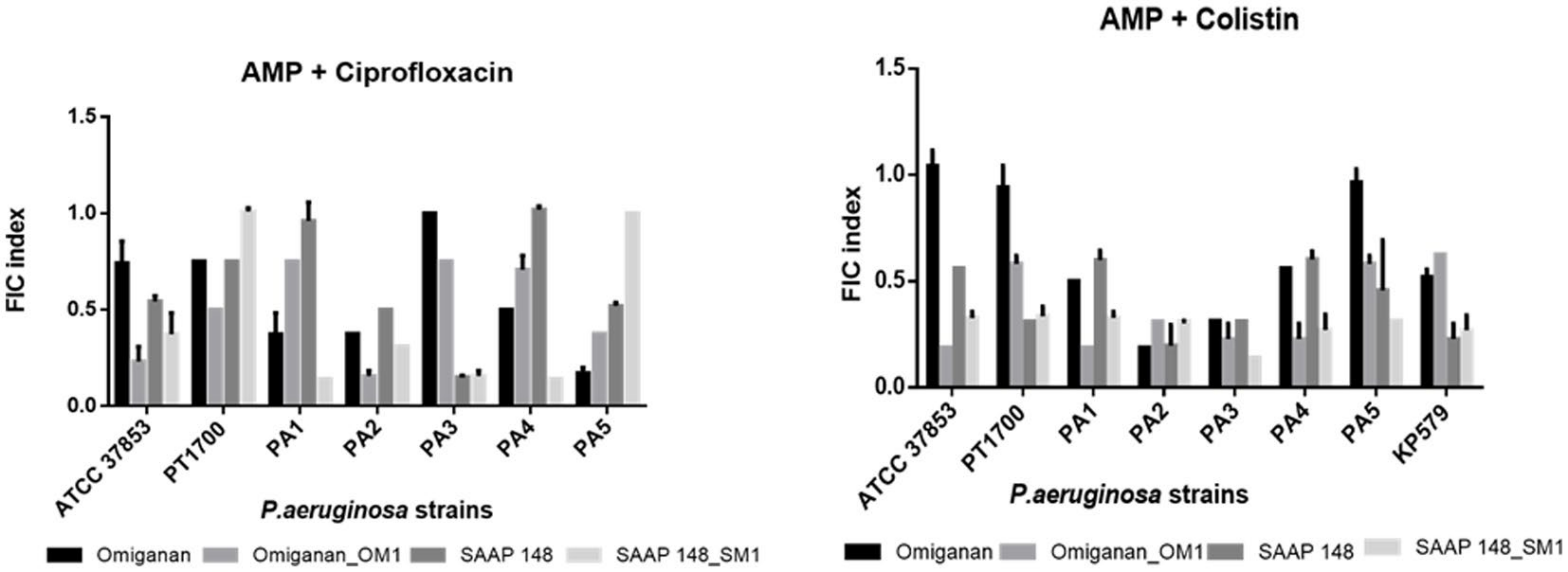
The combinatorial effect of the peptides with **(A)** ciprofloxacin **(B)** colistin. Data is presented as mean ± SD (*n* = 3), *p* < 0.05.

To determine the synergistic activity of the peptides with the clinically used antibiotics, we have carried out the checkerboard assay to evaluate the efficacy of combinations of Omiganan, Omiganan_OM1, SAAP-148, and SAAP-148_SM1 with the ciprofloxacin and colistin. A synergistic activity with ciprofloxacin (Figure 3A) (FIC < 0.5) was observed for SAAP-148_SM1 in ATCC 27853, Omiganan_OM1, SAAP-148 for KP579, Omiganan_OM1 for PT1700, SAAP-148_SM1 in PA1, Omiganan_OM1 and SAAP-148_SM1 for PA2, SAAP-148 and SAAP-148_SM1 for PA3, SAAP-148_SM1 for PA4 and Omiganan, Omiganan_OM1 for PA5. On the other hand, synergistic activity with colistin (Figure 3B) was noticed for Omiganan_OM1 and SAAP_SM1 in ATCC 27853, SAAP-148, SAAP-148_SM1 for KP579, Omiganan_OM1, SAAP-148 and SAAP-148_SM1 for PT1700, Omiganan_OM1 and SAAP-148_SM1 for PA1, Omiganan, Omiganan_OM1, SAAP-148 and SAAP-148_SM1 for PA2, Omiganan, Omiganan_OM1, SAAP-148 and SAAP-148_SM1for PA3, Omiganan_OM1, and SAAP-148_SM1 for PA4 and SAAP-148_SM1 for PA5.

### 3.10 Mechanism of action of the AMPs

**Figure 4:**
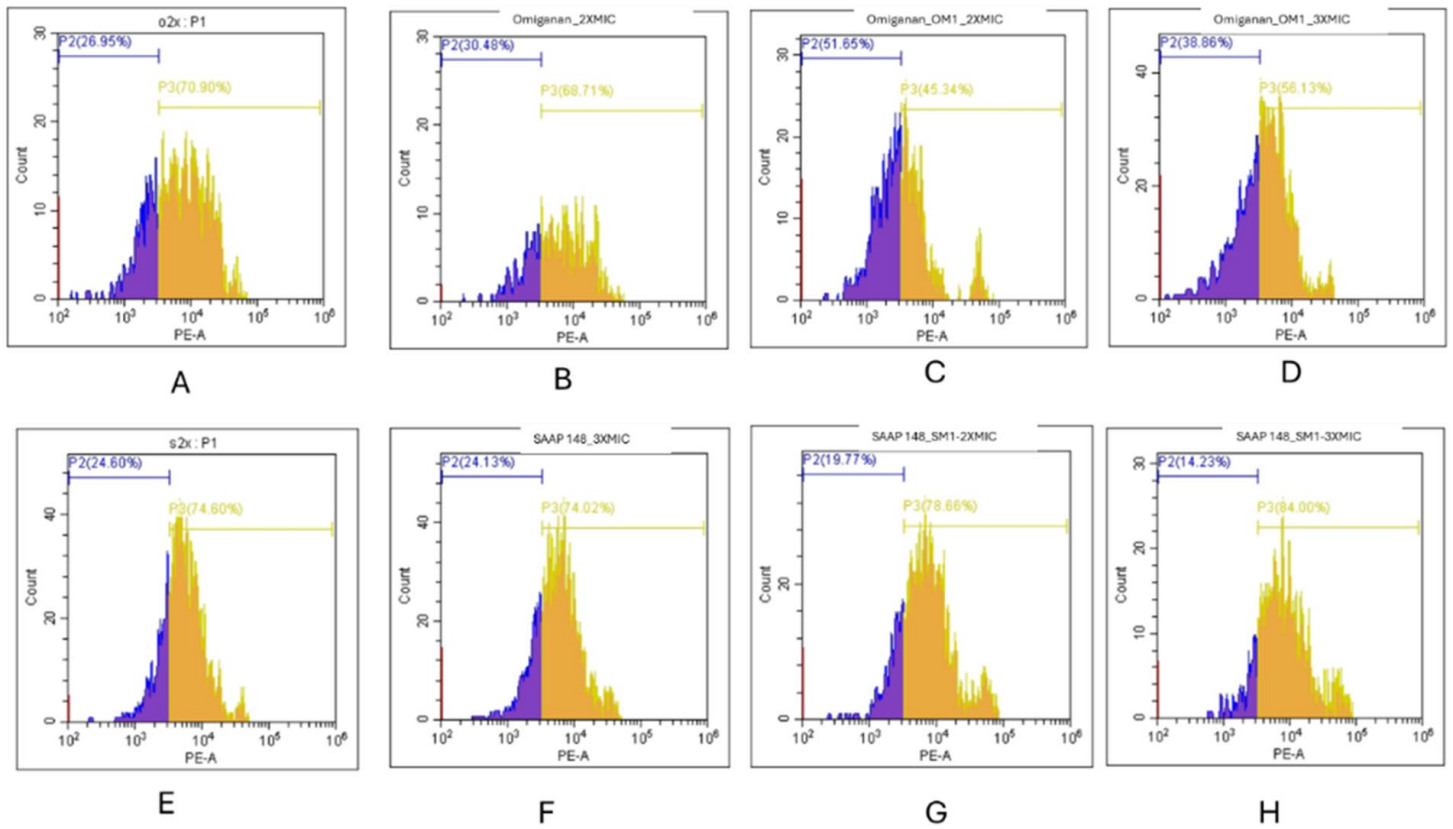
Cell membrane permeabilization assay using Propidium Iodide and analyzed by flow cytometry. **(A)** *P. aeruginosa* ATCC 27853 treated with Omiganan at 2X MIC. **(B)** *P. aeruginosa* ATCC 27853 treated with Omiganan at 3X MIC. **(C)** *P. aeruginosa* ATCC 27853 treated with Omiganan_OM1 at 2X MIC. **(D)** *P. aeruginosa* ATCC 27853 treated with Omiganan_OM1 at 3X MIC. **(E)** *P. aeruginosa* ATCC 27853 treated with SAAP-148 at 2X MIC. **(F)** *P. aeruginosa* ATCC 27853 treated with SAAP-148 at 3X MIC. **(G)** *P. aeruginosa* ATCC 27853 treated with SAAP-148_SM1 at 2X MIC. **(H)** *P. aeruginosa* ATCC 27853 treated with SAAP-148_SM1 at 3X MIC.

We first performed the propidium iodide assay to understand the mechanism of action of AMPs at the membrane level. Propidium iodide fluorescently stains the nucleic acids of the bacterial cells which have undergone cellular membrane disruption. Unstained *Pseudomonas aeruginosa* cells are used to set the negative population. In the absence of the peptides, 6.41% of *P.aeruginosa* showed no PI. The flow cytometric analyses of the peptides revealed 68.71% PI-positive cells for Omiganan (2X MIC) and 70.90% at 3X MIC, 45.34% for Omiganan_OM1 (2X MIC) and 56.31% at 3X MIC, 16.25% for SAAP-148 (2X MIC) and 74.02% at 3X MIC and 28.10% for SAAP-148_SM1 and 84% at 3X MIC (Figure 4). To examine the morphological changes following 24-h treatment with peptides (Omiganan, Omiganan_OM1, SAAP-148, and SAAP 1448_SM1) in *Pseudomonas aeruginosa*, scanning electron microscopy (SEM) imaging was also conducted using the ATCC 27853 strain (Figure 4). Cell wall disruptions, along with alterations in cellular morphology, were observed in the peptide-treated *Pseudomonas aeruginosa* cells, in contrast to the untreated cells.

### 3.11 Effect of the AMPs on efflux pumps

**Figure 5:**
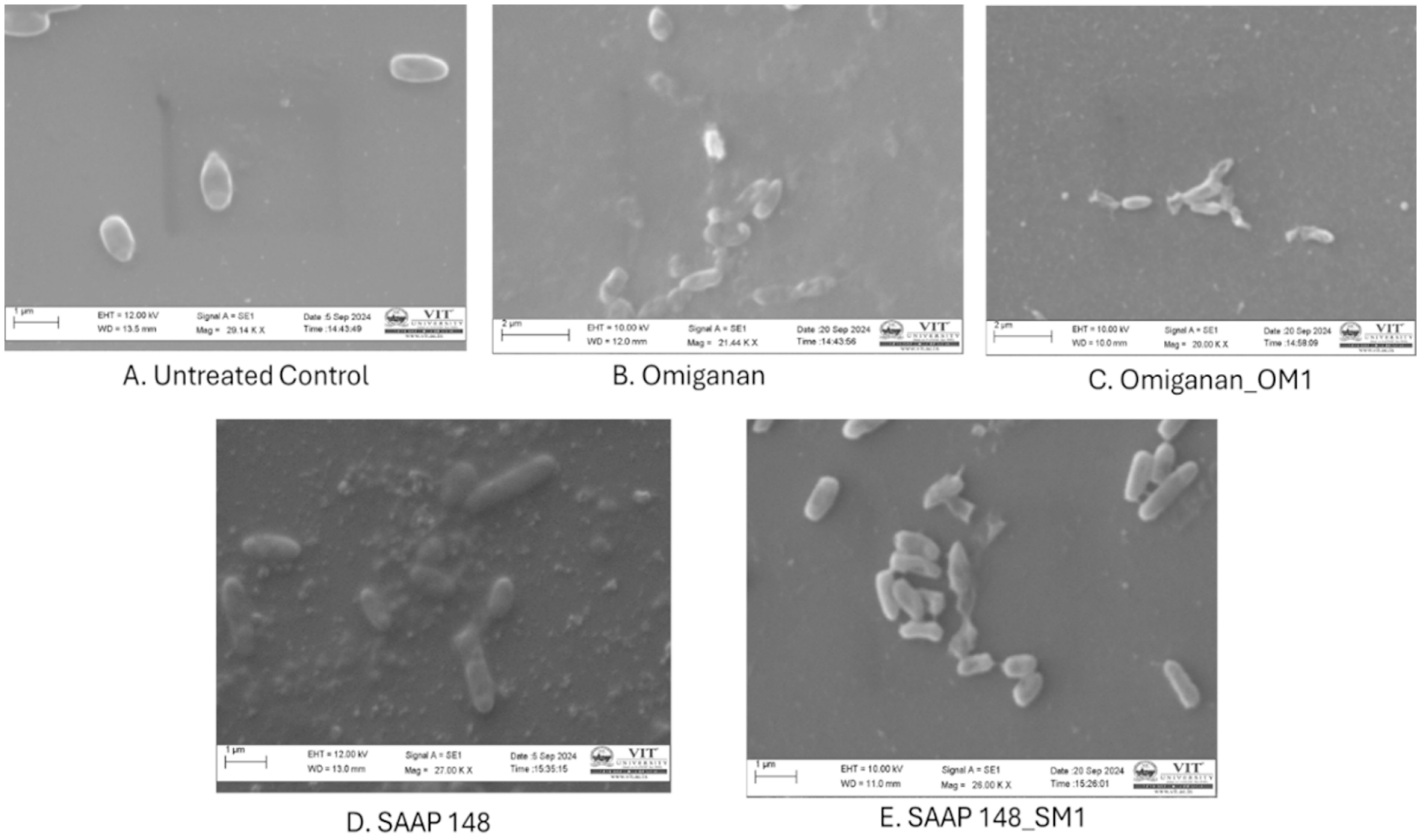
Scanning electron microscope images of *Pseudomonas aeruginosa* treated with AMPs. **(A)** *Pseudomonas aeruginosa* cells without any peptide treatment. **(B)** *P. aeruginosa* treated with Omiganan. **(C)** *P. aeruginosa* treated with Omiganan_OM1. **(D)** *P. aeruginosa* treated with SAAP-148. **(E)** *P. aeruginosa* treated with SAAP-148_SM1.

**Figure 6:**
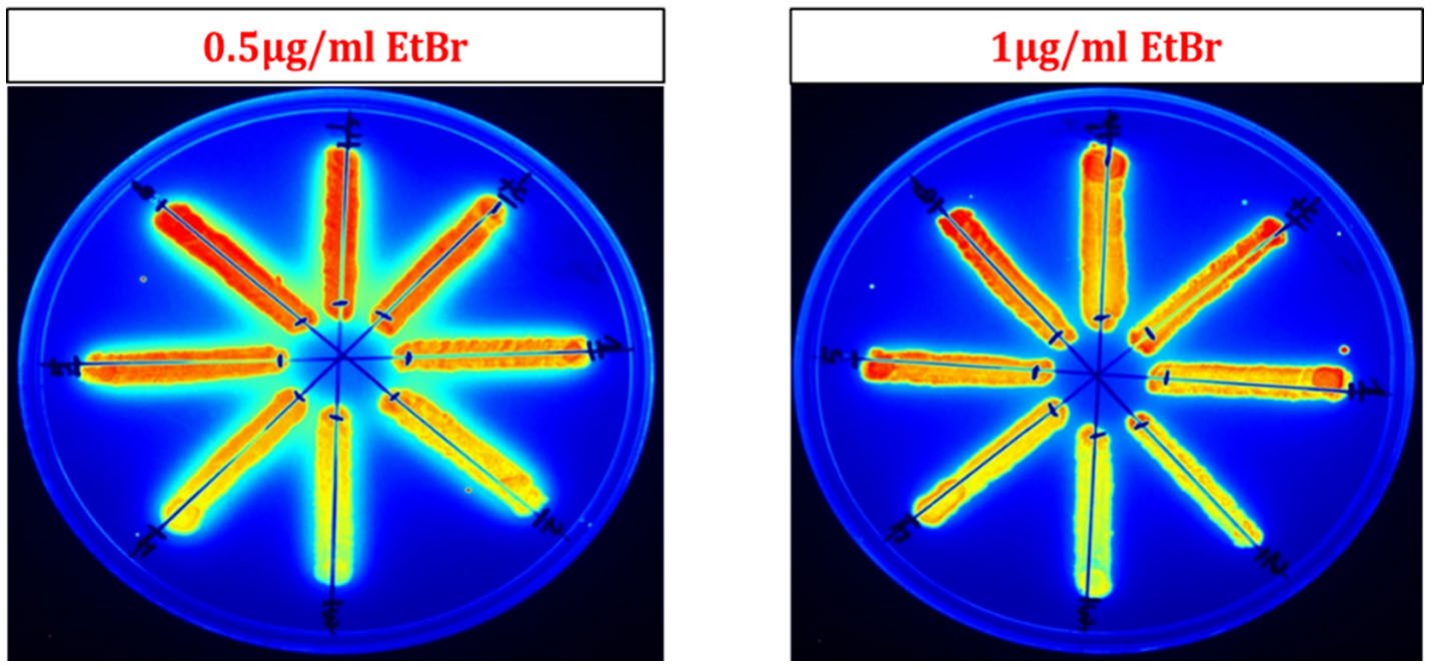
Ethidium bromide cartwheel assay for the inhibition of the efflux pump. Positive control (PC) − ATCC 27853 + 50 μg/mL PaβN, negative control (NC) − ATCC 27853, P1 − SAAP-148, P2 − ciprofloxacin (1 mg/mL), P3 − clove oil, P4 − SAAP-148_SM1 (1X MIC), P5 − omiganan, P6 − omiganan_OM1.

Ethidium bromide Cartwheel assay is widely used for understanding the mechanism of action of the AMPs whether through membrane disruption, inhibition of the efflux pumps, or both. EtBr can intercalate with the bacterial DNA and produce red color fluorescence. If an efflux pump is upregulated, the amount of fluorescence detected in the assay would be lower and if the AMP can inhibit the pumps, then the fluorescence will be higher. At 0.5 μg/mL (Figure 5), the highest fluorescence was observed for peptides Omiganan, and Omiganan_OM1 and at 1 μg/mL, the highest fluorescence intensity was observed for 3 peptides i.e., Omiganan, Omiganan_OM1, and SAAP-148 (Figure 6).

### 3.12 Safety evaluation of the AMPs

#### 3.12.1 Hemolysis inhibition assay

**Figure 7:**
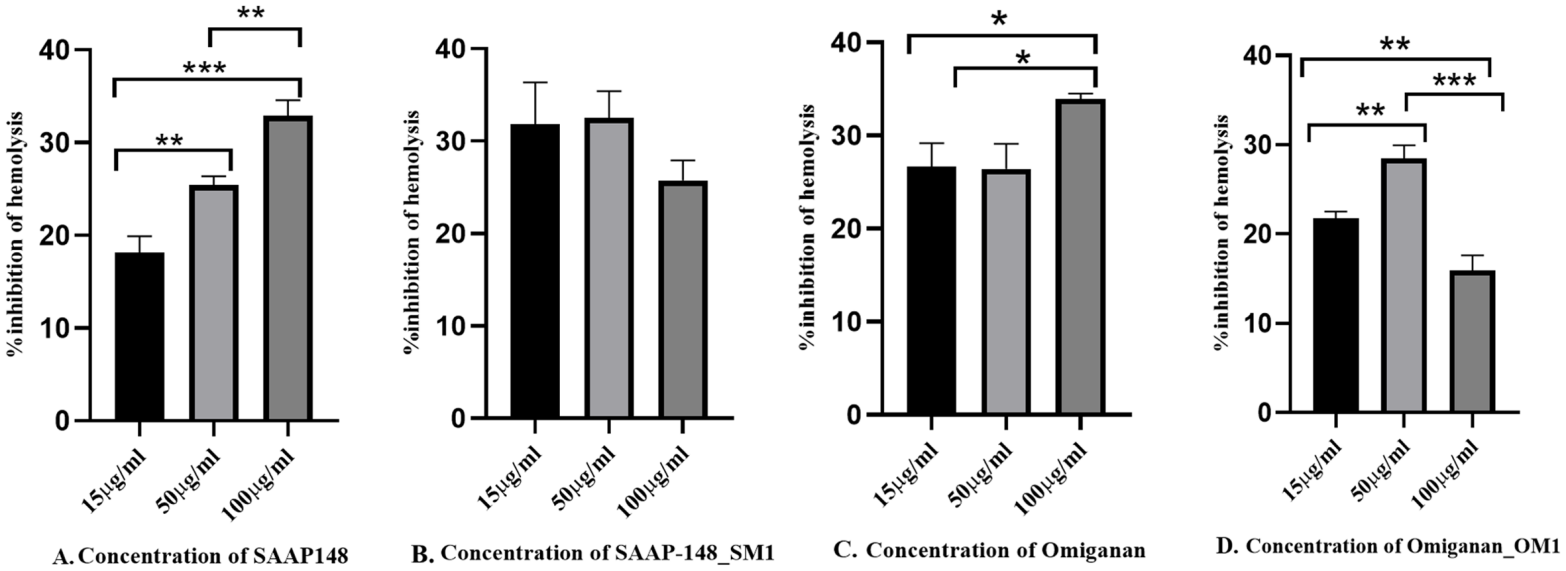
Hemolysis inhibition percentage of the antimicrobial peptides at concentrations 15, 50, and 100 μg/mL. **(A)** Hemolysis inhibition percentage of SAAP-148. **(B)** Hemolysis inhibition percentage of SAAP-148_SM1. **(C)** Hemolysis inhibition percentage of Omiganan. **(D)** Hemolysis inhibition percentage of Omiganan_OM1. Data is presented as mean ± SD (*n* = 3), **p < 0.05. “*”on the bar graphs denote statistical significance.

The inhibition percentage of heat-induced red blood cell hemolysis at different concentrations (15, 50, and 100 μg/mL) of the four different antimicrobial peptides (Omiganan, Omiganan_OM1, SAAP-148, and SAAP-148_SM1) was shown in Figure 7. The peptides significantly inhibited the haemolysis at lower concentrations, i.e., 15 and 50 μg/mL but a considerable decrease in the inhibition was observed as the concentration of the peptides increased to 100 μg/mL.

#### 3.12.2 Cytotoxicity assay on HEK cells and HEPG2 cells

**Figure 8:**
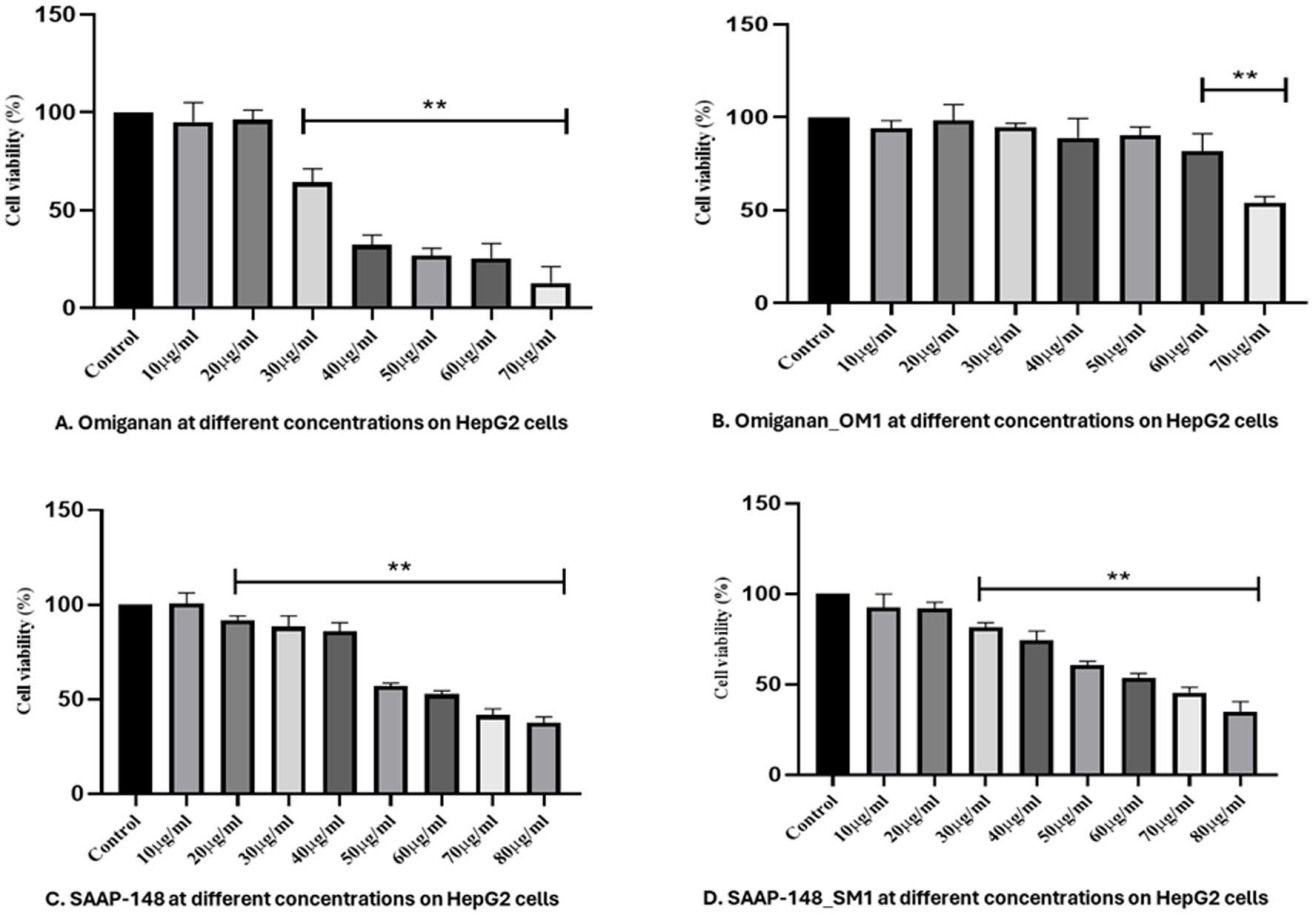
Effect of the antimicrobial peptides on HepG2 cell viability at concentrations ranging from 10 μg/mL–80 μg/mL. **(A)** Effect of Omiganan on HepG2 cells. **(B)** Effect of Omiganan_OM1 on HepG2 cells. **(C)** Effect of SAAP-148 on HepG2 cells. **(D)** Effect of SAAP-148_SM1 on HepG2 cells. Data is presented as mean ± SD (*n* = 3), **p < 0.05.

The cell viability treated with varying concentrations of antimicrobial peptides was assessed using an MTT assay. Omiganan exhibited no toxicity at concentrations ranging from 10 to 80 μg /mL (Figure 8A), and SAAP-148_SM1 was safe on HEK cell lines at concentrations between 10 and 40 μg /mL (Figure 8D). In contrast, SAAP-148 (Figure 8C) and Omiganan (Figure 8B) significantly reduced the HEK cell viability from 20 to 80 μg /mL. On the other hand, Omiganan_OM1 and SAAP-148_SM1 were relatively safer on the HepG2 cells when compared with the other peptides such as Omiganan and SAAP-148 from concentrations ranging from 20 to 80 μg/mL.

## 4 Discussion

In recent years, the efficacy of conventional antibiotics has diminished due to the increasing prevalence of multidrug-resistant (MDR) bacteria. A promising strategy to combat MDR bacteria involves using antimicrobial peptides (AMPs) that specifically target and inhibit efflux pump proteins, as an alternative to traditional antibiotics (Aparna et al., 2014). In the current situation, the combination of AMPs with traditional antibiotics could be a treatment choice but it possesses potential challenges. One of the major concerns is the potential for antagonistic interactions where certain combinations may reduce the efficacy rather than enhance the other one. AMPs are often susceptible to enzymatic degradation and reduce half-life, *in vivo*. Addressing these challenges requires rigorous *in silico*, *in vitro* studies coupled with *in vivo* validations (Gonçalves et al., 2025). In this study, we have focused on identifying antimicrobial peptides against the MexB efflux pump of *Pseudomonas aeruginosa,* which is an important factor in the intrinsic mode of resistance against several antibiotics, such as fluoroquinolones, aminoglycosides, beta-lactams, macrolides, and several other antimicrobial agents (Pang et al., 2019). MexAB-OprM is a crucial pump that plays a significant role in the MDR mechanism of *P. aeruginosa*. Upon overexpression of efflux pumps, antibiotics are expelled out of the cell through the OprM porin channel, a part of the MexAB- OprM efflux pump complex (Pesingi et al., 2019).

**Figure 9:**
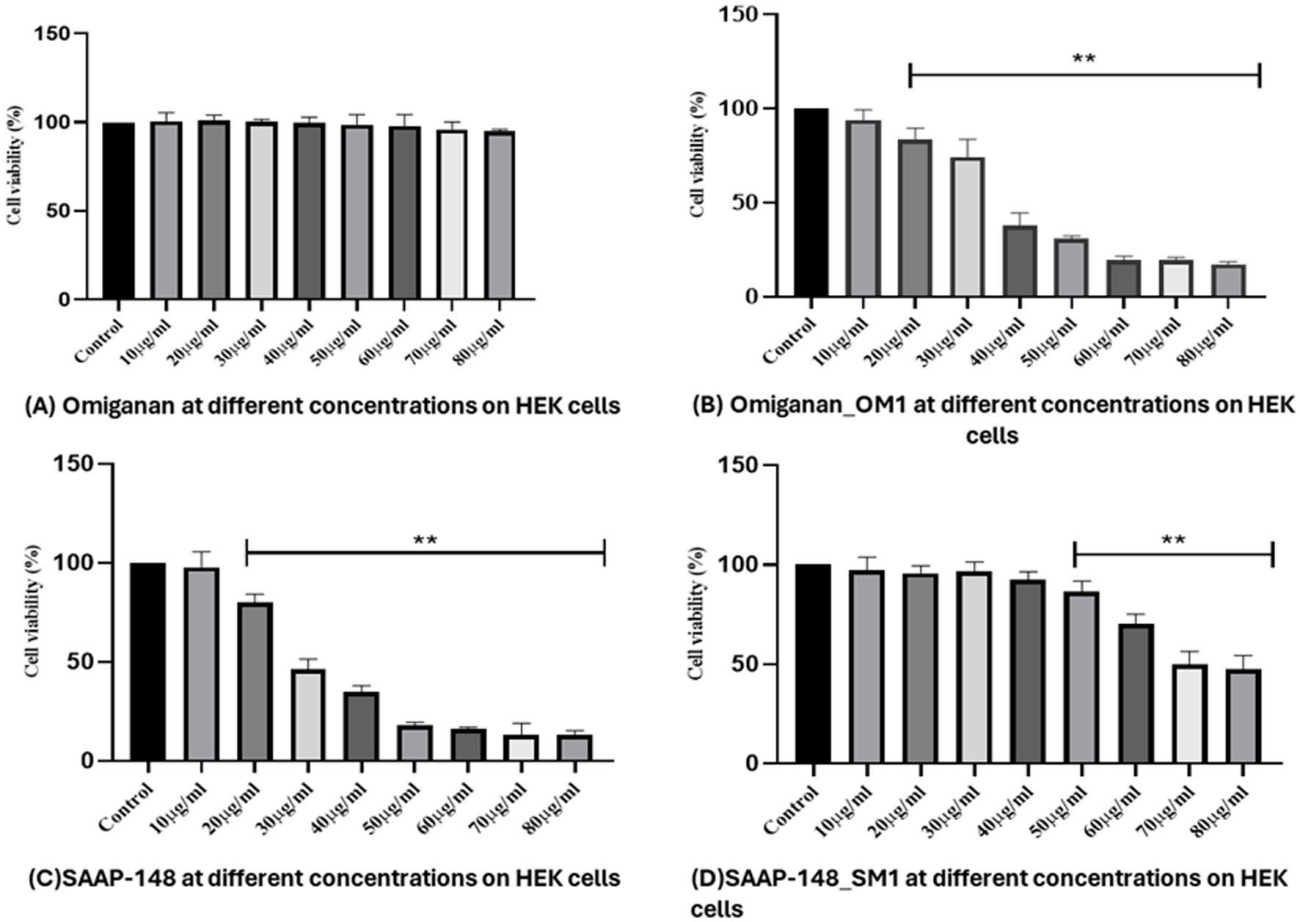
Effect of antimicrobial peptides on the cell viability of HEK293. The results are expressed as *p* < 0.05 versus control, *n* = 6. **(A)** Effect of Omiganan on HepG2 cells. **(B)** Effect of Omiganan_OM1 on HepG2 cells. **(C)** Effect of SAAP-148 on HepG2 cells. **(D)** Effect of SAAP-148_SM1 on HepG2 cells. Data is presented as mean ± SD (*n* = 3), *p* < 0.05. ”*” on the bar graphs denote statistical significance.

Database mining using the HPEPDOCK 2.0 server led us to the shortlisting of the 10 AMPs against the MexB protein. The peptide in its natural state generally exhibits low stability and higher cytotoxicity parameters. Consequently, methods including the substitution of the amino acids were carried out to enhance the peptides’ antimicrobial activity, hydrophobicity, net charge, and diminished cytotoxicity (Kumar et al., 2019; Porto et al., 2018; Liu et al., 2007). The molecular docking study using ClusPro 2.0 of 10 parent peptides and their analogs showed the highest binding energy (SAAP-148_SP57/SAAP-148_SM1) and the lowest binding energy (Mutacin_MU1). The peptides in the shortlisted category were non-toxic and non-immunogenic, with a length of ≤ 30 amino acids hence more stable as compared to the longer ones (Yadav et al., 2020). The parent peptides and the mutant peptides have almost 3–4 lysine residues indicating an increased positive charge contributing to their antimicrobial activity against both Gram-negative and Gram-positive bacteria (Rice and Wereszczynski, 2017). Upon docking with the MexB protein and carrying out molecular dynamics simulations, we observed a phenomenon close to what we hypothesized. The RMSD findings indicate that peptide binding influences the overall stability of the MexB protein. MexB-SAAP-148_SM1 and Omiganan_OM1 had lower RMSD values as compared to their parents, Omiganan and SAAP-148, signifying enhanced stability, which could impact their functionality. The RMSF studies revealed that the area around residue 200 exhibits increased flexibility, potentially influencing the protein’s efflux mechanism significantly, as observed in our study when MexB was complexed with FDA-approved drug molecules (Chatterjee and Sivashanmugam, 2024). Whereas, a different trend was observed while studying the RMSF trajectory, where Omiganan’s presence seems to stabilize the loop region around 200 residues, hence limiting oscillations that may compromise protein function but the other peptides enhanced flexibility in the C-terminal region, potentially suggesting a destabilizing impact (Rai et al., 2021). This instability may enable essential conformational alterations during substrate translocation; however, it could also heighten the likelihood of misfolding or functional loss (Shukla and Tripathi, 2020). Upon analyzing the SASA trajectory, we observed that the mutants, particularly the APO C1 mutant, Omiganan_OM1, and SAAP 148_SM1, exhibited lower SASA values compared to their parent molecules. Notably, this reduction did not result in significant destabilization or increased solvent exposure (Kushwaha et al., 2021). Furthermore, the Rg values indicate that most peptide-bound complexes exhibit a stable degree of compactness (3.8–3.9 nm), hence enhancing the stability of the protein-peptide complexes through tighter packing (Ul Haq et al., 2017). The peptide Omiganan_M14/Omiganan_OM1 routinely establishes 10–15 hydrogen bonds with MexB, indicating strong and dynamic interactions as compared to its parent counterpart Omiganan that likely enhance the overall stability of this complex. The presence of β-sheet structures, especially in Omiganan_M14/Omiganan_OM1, Omiganan, SAAP 148_SM1, indicates improved interaction dynamics, as these motifs may enable various binding modes and potentially enhance affinity for MexB (Dathe and Wieprecht, 1999). Thus, from the *in silico* study, we have observed that substituting parent peptides with lysine, cysteine, isoleucine, and proline can significantly alter their structural properties, thereby influencing molecular dynamics (MD) simulations and interactions with target proteins. Lysine, a positively charged residue, enhances electrostatic interactions and hydrogen bonding, potentially increasing peptide solubility and binding affinity. Cysteine, with its thiol group, can form disulfide bonds, stabilize the peptide structure, but also alter flexibility depending on the redox environment. Isoleucine, a hydrophobic residue, strengthens hydrophobic interactions, potentially improving binding to nonpolar regions of the target but may reduce solubility. Proline, known for its rigid cyclic structure, disrupts *α*-helices and introduces conformational constraints, affecting peptide folding and dynamics. These structural changes impact MD simulations by altering peptide stability, solvent accessibility, and binding affinity to the target protein (Basu et al., 2022). Hence, Omiganan, Omiganan_OM1, SAAP 148, and SAAP_148_SM1 necessitated *in vitro* validation to ensure that structural modifications enhance the desired bioactivity while maintaining stability and solubility. To discuss, the molecular docking analysis demonstrated that several mutant peptides exhibited improved binding affinity toward the MexB efflux pump of *P. aeruginosa* compared with their corresponding parent peptides, suggesting enhanced interaction with the target binding pocket. Structural analysis of the docked complexes indicated that substitutions in the mutant peptides increased the number of stabilizing interactions, including hydrogen bonding, electrostatic interactions, and hydrophobic contacts with key residues lining the substrate-binding cavity. In particular, the introduction of positively charged residues likely strengthened electrostatic interactions with negatively charged amino acids within the MexB binding region, thereby improving peptide anchoring and stability. Molecular dynamics simulations further supported these findings by demonstrating stable peptide–protein complexes throughout the simulation trajectory, with minimal fluctuations in RMSD and sustained intermolecular contacts (Figure 9).

A multitude of antimicrobial peptides have been examined in vitro for their capacity to impede *P. aeruginosa* infections. The antibacterial efficacy of CM4, a cationic antimicrobial peptide sourced from the silkworm *Bombyx mori*, with a MIC of 18 μM (Dathe and Wieprecht, 1999) and 6 K-F17, a synthetic peptide with MIC values between 2 and 256 μg/mL, displayed inhibitory action against many species of *P. aeruginosa* (Beaudoin et al., 2018). The *in-silico* findings were corroborated by evaluating the shortlisted AMPs against multiple strains of *P. aeruginosa*. Omiganan, a potent topical AMP shows promising potential as a new-generation therapeutic because of its membrane disruption capabilities. It was found to have powerful anti-bacterial and anti-fungal activity, but was found to be more effective in combination with other drugs (Wesseling and Martin, 2022; Żyrek et al., 2021). In the present study, the MIC of Omiganan was ≤ 64 μg/mL against several strains of *Pseudomonas aeruginosa*. Conversely, Omiganan_OM1, a mutant of Omiganan, demonstrated a broad spectrum of minimum inhibitory concentrations (MIC) ranging from 16 to 32 mg/L for most of the strains. According to Fritsche et al. (2008), the MIC 50 value of Omiganan against *E. coli* was 32 mg/L, whereas the MIC 50 value for *Klebsiella pneumoniae* was 128 mg/L (Fritsche et al., 2008). Similarly, in the present study, SAAP-148 and SAAP-148_SM1 showed MIC ranging from 8 to 64 μg/mL whereas the analog of SAAP-148_SM1 inhibits biofilm at 32 μg/mL. Previous studies have shown that *E. coli*, *P. aeruginosa*, and *S. epidermidis* showed higher susceptibility to SAAP-148 in comparison to *K. pneumoniae* and *S. aureus* in the range between 10 and 160 μg/mL (Gan et al., 2024). The checkerboard assay was performed to study the combinations of antibiotics and potential AMPs as a screening method that gives the FIC index used to interpret the combinations (Black et al., 2023). A synergistic effect among all combinations tested was observed by combining Omiganan and Ciprofloxacin or Omiganan and Colistin against the quality strain *P. aeruginosa* ATCC 37853 and test strains PA1, PA4, and KP579. A combination of SAAP-148_SM1 along with Ciprofloxacin or colistin was effective against all the *P. aeruginosa* strains. Prior research demonstrated that SAAP-148 and Halicin were utilized to synergistically eradicate biofilms produced by *E. coli*, *A. baumanii*, and *K. pneumoniae* (Bouhrour et al., 2023).

Here, we have also observed that Omiganan and SAAP-148 analogs block efflux pump activity, thereby enhancing the intracellular concentration of antibiotics. Indolicidin, a cationic antimicrobial peptide, has recently been shown to synergistically enhance the efficacy of aminoglycoside antibiotics against *P.aeruginosa*, indicating a dual approach involving membrane rupture and inhibition of efflux pump (Taheri-Araghi, 2024). A fluorometric study utilizing propidium iodide and SEM imaging was performed to elucidate the underlying mechanism of action of the AMPs. A significant increase in fluorescence percentage was noted in AMP-treated bacterial cells, suggesting enhanced cell permeability of the AMPs relative to the control, which correlated with the cellular damage observed in SEM images. The Ethidium bromide cartwheel assay reveals that the accumulation levels of ethidium bromide in *P. aeruginosa* ATCC27853 correlate with efflux pumps and the resistance mechanism (Silpa et al., 2022).

Antimicrobial peptides demonstrate extensive antimicrobial efficacy; however, cytotoxicity poses a significant challenge for their systemic use (Zhu et al., 2015). In our study, we evaluated the toxicity of AMPs using HepG2 and HEK 293 cells. Our findings indicate that Omiganan and SAAP-148_SM1 exhibit relatively lower toxicity on HepG2 cells at higher concentrations, while Omiganan_OM1 demonstrates moderate toxicity on HEK293 cells. Moderate safety was observed for the SAAP-148_SM1 variant.

Finally, from the methods adopted and the results obtained from the study, we can discuss that the antimicrobial susceptibility assays demonstrated effective growth inhibition of the tested strains following peptide treatment, membrane permeability analyses indicated increased uptake of propidium iodide in treated cells, confirming disruption of membrane integrity. Scanning electron microscopy further revealed morphological alterations in peptide-treated bacterial cells, consistent with membrane destabilization and cellular damage. Taken together, these *in vitro* and *in silico* findings support the hypothesis that the peptides may enhance intracellular accumulation of antibacterial agents by compromising membrane integrity and potentially interfering with the efflux pump function, thereby sensitizing multidrug-resistant *P. aeruginosa* to antimicrobial stress.

## 5 Conclusion

The comprehensive analysis presented in this study highlights the intricate interplay between peptide structure, binding affinity, and the resultant conformational dynamics of the MexB protein. Antimicrobial peptides, Omiganan, SAAS-148, and mutant variants having high binding affinities against *P. aeruginosa* MexB efflux pump were studied in this investigation. Synthesized AMPs showed strong antibacterial action, inhibited biofilm formation, and worked in tandem with traditional antibiotics. They were shown to be involved in membrane disruption and efflux pump inhibition and were found to be less toxic to human cells. AMPs show promise as therapeutic agents against multidrug-resistant *P. aeruginosa*, enabling *in vivo* and clinical research. Future studies focusing on optimizing peptide structures, investigating the effects of various modifications, and exploring potential synergies with existing antibiotics may pave the way for innovative strategies to mitigate the impact of antibiotic resistance in clinical settings.

## Data Availability

The original contributions presented in the study are included in the article/Supplementary material, further inquiries can be directed to the corresponding author.

## Footnotes

1
http://imed.med.ucm

2
https://bestsel.elte.hu/
