# Supplementary material for: From computational screening to in vitro validation: exploring antimicrobial peptides against Pseudomonas aeruginosa
Source: Front Microbiol. 2026 Jun 11;17:1796090. doi: 10.3389/fmicb.2026.1796090 (PMC13294467; doi:10.3389/fmicb.2026.1796090)
Supplement: Supplementary file 2 [file Table_2.DOCX]

Supplementary Dataset 1: List of antimicrobial peptides retrieved from publicly available databases

| **APD3** | |
| --- | --- |
| **Peptide Name** | **Peptide Sequence** |
| a9 | WEPNFGFSLSKYF |
| ecPis-2 | FFFHIIKGLFHAGRMIHGLV |
| SAAP-148 | LKRVWKRVFKLLKRYWRQLKKPVR |
| oc_45 | MVVVGIVVGVVGIILLAFVAPLTWGFIGVM |
| Chicken CATH-2 | RFGRFLRKIRRFRPKVTITIQGSARFG |
| Omiganan | ILRWPWWPWRRK |
| APOC1 | FSTKTRNWFSEHFKKVKEKLKDTFA |
| Pleurocidin | GWGSFFKKAAHVGKHVGKAALTHYL-NH2 |
| PcAst-1a | SNGYRPAYRPAYRPSYRPGK |
| Competence Stimulating Peptide | SGSLSTFFRLFNRSFTQALGK |
| git_8 | MVRILHSFLITKCIGSMRTFMPYCTCLAFR |
| Mutacin II | NRWWQGVVPTVSYECRMNSWQHVFTCC |
| YR26 | YYHFWHRGVTKRSLSPHRPRHSRLQR |
| Clavanin A | VFQFLGKIIHHVGNFVHGFSHVF |
| git_11 | MGMALLALCFCALSLVLALFVGILCLVFG |
| RP7 | FFGRLKAVFRGARQGWKEHRY |
| Arenicin-3 | GFCWYVCYRNGVRVCYRRCN |
| git_14 | MKKFYTKRFSLICAFAASALLLGGGMQRG |
| HKPLP | FLGLIFHGLVHAGKLIHGLIHRNR |
| TP3 | FIHHIIGGLFSVGKHIHSLIHGH |
| Chrysophsin-1 | FFGWLIKGAIHAGKAIHGLIHRRRH |
| moroNC-NH2 | FFWHHIGHALDAAKRVHGMLSG |
| ecPis-3 | IFGLLLHGAIHVGKLIHGLVRRH |
| Oreoch-1 ( | FIHHIIGGLFSVGKHIHGLIHGH |
| OP-145 | IGKEFKRIVERIKRFLRELVRPLR |
| git_16 | MMAFLLSVGVPVGVILFFALTLTVSVMSS |
| oc_4 | MVVAKVAKTKPRKKKGMSLRAFIMLMTMA |
| oc_19 | MVVAKVAKTKPRKKKGMSLRAFIMLMTMA |
| [K18]-Moronecidin | FFHHIFRGIVHVGKTIHKLVT |
| Pis1 | FIHHIIGWISHGVRAIHRAIHG |
| WLBU2, | RRWVRRVRRWVRRVVRVVRRWVRR |
| Tritrpticin | VRRFPWWWPFLRR |
| BTM-P1 | VAPIAKYLATALAKWALKQGFAKLKS |
| Mag 2 | GIGKFLHSAKKFGKAFVGEIMNS |
| Chionodracine | FFGHLYRGITSVVKHVHGLLSG |
| Horine | WWWLRRRW |
| oc_26 | MTNQISTATKQYFYWFFIQRTGKELFVVR |
| SmHep1P | QSHISLCRWCCNCCKANKGCGFCCKF |
| Trematocine | FFGHLLRGIVSVGKHIHGLITG |
| CPF-St4 | SLFGTFAKMALKGASKLIPHLLPSRQQ |
| Ib-AMP1 | QWGRRCCGWGPGRRYCVRWC |
| sb-Moronecidin | FFHHIFRGIVHVGKTIHRLVTGT |
| Verine | RRRWWWWV |
| Piscidin 1 | FFHHIFRGIVHVGKTIHRLVTG |
| Mellitin (MELT) | GIGAVLKVLTTGLPALISWIKRKRQQ |
| oc_1 | MIIVNTFRNSFFRLLTTYADTRAFKIFTS |
| Moronecidin-like | FFRNLWKGAKAAFRAGHAAWRA |
| Caerin 1.18 | GLFSVLGSVAKHLLPHVVPVIAEKL |
| Greater amberjack piscidin | FIHHIIKGIFHIGKIIHSAINRRRH |
| Latarcin 1 | SMWSGMWRRKLKKLRNALKKKLKGE |
| git_4 | MLNIWYILIKYFIKYCKDNKYIYICDVIIE |
| Gramicidin A | VGALAVVVWLWLWLW |
| Codesane | GMASLLAKVLPHVVKLIK |
| Hedistin | LGAWLAGKVAGTVATYAWNRYV |
| Streptococcin A-FF22 | GKNGVFKTISHECHLNTWAFLATCCS |
| OH-CM6 | KFFKKLKKAVKKGFKKFAKV |
| Salusin-beta | AIFIFIRWLLKLGHHGRAPP |
| ToAP2 | FFGTLFKLGSKLIPGVMKLFSKKKER |
| Css54 | FFGSLLSLGSKLLPSVFKLFQRKKE |
| skin_5 | MKKRKNFFEYIFIVIKYINNVLVIINIELF |
| LGH2 | GNPLKLFLPSTWVHFFKFLR |
| Temporin-L | FVQWFSKFLGRIL |
| wb-Moronecidin | FFHHIFRGIVHVGKTIHKLVTGT |
| Ixosin | GLHKVMREVLGYERNSYKKFFLR |
| CRP-AC1 | GMWGTVFKGIKTVAKHLLPHVFSSQQS |
| Galensin | CYSAAKYPGFQEFINRKYKSSRF |
| Meucin-22 | FFGHLFKLATKIIPSLFQRKKE |
| Pc-CATH1 | RIKRFWPVVIRTVVAGYNLYRAIKKK |
| BacCH91 | ITSFIGCTPGCGKTGSFNSFCC |
| Rbpisc | GEGFLGMLLHGVGHAIHGLIHGK |
| oc_25 | MPALVKTMFSTREKNGKDWDYWYHGKMEK |
| PseA | GDCGGTCTWTKDCSICPSWSCWSWSC |
| Senegalin | FLPFLIPALTSLISSL |
| Ruminococcin A | GNGVLKTISHECNMNTWQFLFTCC |
| Ascaphin-1 | GFRDVLKGAAKAFVKTVAGHIAN |
| Plantaricin CS | GGYKNFYGSALRKGFYEGEAGRAIRR |
| Pac-525 | KWRRWVRWI |
| Im6 | FFFLPSLIGGLVSAIK |
| Gramicidin B | VGALAVVVWLFLWLW |
| Bthepc | QSHLSLCRWCCNCCHNKGCGFCCKF |
| cAMP1009 (cAMP_1009; Predicted, gut microbiome, | KMAVKVAINGFGRIGRLAFRQMFG |
| Phylloseptin-H2 | FLSLIPHAINAVSTLVHHF |
| Lividin-1 | ILPFVAGVAAEMMQHVYCAASKKC |
| Peptide L1 | VNFLLHKIYGNIRYS |
| puro a | FPVTWKWWKWWKG-NH2 |
| oc_34 | MIAIVGQYKFADHDFGRIDAPAFSKVTFK |
| Shepherin I | GYGGHGGHGGHGGHGGHGGHGHGGGGHG |
| LyeTx I | IWLTALKFLGKNLGKHLAKQQLAKL |
| Magainin-F3 | GVSKILHSAGKFGKAFLGEIMKS |
| CPF-B1 | GLGSLLGKAFKIGLKTVGKMMGGAPREQ |
| ChBac3.4 | RFRLPFRRPPIRIHPPPFYPPFRRFL |
| buCATHL4A | GLPWILLRWLFFRG |
| puro b1 | FPVTWPTKWWKG-NH2 |
| Palustrin-1b | ALFSILRGLKKLGNMGQAFVNCKIYKKC |
| Plantaricin A | KSSAYSLQMGATAIKQVKKLFKKWGW |
| Polyphemusin I | RRWCFRVCYRGFCYRKCR |
| Arenicin-2 | RWCVYAYVRIRGVLVRYRRCW |
| Hs-1 | FLPLILPSIVTALSSFLKQG |
| P-04 | FSLFFPYAALKWLRKLLKK |
| lacticin LMG | GNGVIKTVSHECKMNTWQFLFTCCS |
| Androctonin | RSVCRQIKICRRRGGCYYKCTNRPY |
| Human neutrophil peptide-2 | CYCRIPACIAGERRYATCIYQGRLWAFCC |
| Lycotoxin I | IWLTALKFLGKHAAKHLAKQQLSKL |
| PP13 | GAARKSIRLHRLYTWKATIYTR |
| Smp24 | IWSFLIKAATKLLPSLFGGGKKDS |
| Isracidin | RPKHPIKHQGLPQEVLNENLLRF |
| VLL-28 | VLLVTLTRLHQRGVIYRKWRHFSGRKYR |
| VLL-28 | VLLVTLTRLHQRGVIYRKWRHFSGRKYR |
| Peptide 8557 | WKIFKLKLRMLW |
| ZY4 | VCKRWKKWKRKWKKWCV |
| Lycosin-I | RKGWFKAMKSIAKFIAKEKLKEHL |
| H4 | KFKKLFKKLSPVIGKEFKRIVERIKRFLR |
| Marcin-22 | FFGHLFKLATKIIPSFFRRKNQ |
| Ctriporin | FLWGLIPGAISAVTSLIKK |
| g11 | MSYLQRYKILRKNMGEQPLHKPTFQEHYWK |
| ALF-E | GCYVNRSPYLKKFEVHYRADVKCG |
| Clavaspirin | FLRFIGSVIHGIGHLVHHIGVAL |
| RV-23 | RIGVLLARLPKLFSLFKLMGKKV |
| AamAP1 | FLFSLIPHAIGGLISAFK |
| Alarin | APAHRSSTFPKWVTKTERGRQPLRS |
| Ranacyclin-B-AL1 | AAFRGCWTKNYSPKPCL |
| Em-Pis1M | FIFHIIKGLFHAGKMIHGLVTRRRH |
| PROTEGRIN-1 | RGGRLCYCRRRFCVCVGR |
| Protegrin 1 | RGGRLCYCRRRFCVCVGR |
| g2 | RGTCYNRVGLIIRNFSKLKGKKV |
| cAMP1655 (pAMP_1655; candidate AMP 1655; Predicted, gut microbiome | RGTCYNRVGLIIRNFSKLKGKKV |
| g5 | MGGVLLAGIIMGLASAGLLGALSTLFSVG |
| Frenatin 3 | GLMSVLGHAVGNVLGGLFKS |
| Buforin II | TRSSRAGLQFPVGRVHRLLRK |
| KYE21 | KYEITTIHNLFRKLTHRLFRR |
| GzLEAP-2B | MTPLWRILASKPQGAYCHDHIECSTGLCR |
| Trichoplaxin | FFGRLKSVWSAVKHGWKAAKSR |
| Gaegurin-RN1 | FIGPVLKIAAGILPTAICKIFKKC |
| Lycosin-II | VWLSALKFIGKHLAKHQLSKL |
| Variacin | GSGVIPTISHECHMNSFQFVFTCCS |
| Peptide VR-23 | VIGSILGALASGLPTLISWIKNR |
| puro b4 | FAVTWATKWWKG-NH2 |
| Epinecidin-1 | GFIFHIIKGLFHAGKMIHGLV |
| Hominicin | ITPATPFTPAIITEITAAVIA |
| Pseudhymenochirin-1Pb | IKIPSFFRNILKKVGKEAVSLIAGALKQS |
| Streptin 1 ( | VGSRYLCTPGSCWKLVCFTTTVK |
| ug_1 | MPNGKKKKGHKMATHKRKKRLRKNRHKSK |
| Figainin 1 | FIGTLIPLALGALTKLFK |
| Kunitzin-RE | AAKIILNPKFRCKAAFC |
| Peptide #22 | LLGAALSALSSVIPSVISWFQK |
| XPF-St4 | GWASSIGSILGKFAKGGAQAFLQPK |
| TsAP-2 | FLGMIPGLIGGLISAFK |
| Gallidermin | IASKFLCTPGCAKTGSFNSYCC |
| Tachyplesin I | KWCFRVCYRGICYRKCR-NH2 |
| g7 | MLTVGVALVMVTVVGSLIAGSVPTVISTV |
| Hepcidin 25 | DTHFPICIFCCGCCHRSKCGMCCKT |
| Peptide 536_1 | RWRLVCFLCRRKKV |
| Hejiangin-A1 | RFIYMKGFGKPRFGKR |
| PR-bombesin | QKKPPRPPQWAVGHFM |
| Phylloxin-B1 | GWMSKIASGIGTFLSGMQQ |
| Pandinin 2 | FWGALAKGALKLIPSLFSSFSKKD |
| Latarcin 3a | SWKSMAKKLKEYMEKLKQRA |
| Drosophila MPAC | QRPYTQPLIYYPPPPTPPRIYRA |
| Deserticolin 1 | GLADFLNKAVGKVVDFVKS |
| Megin 1 | FLKGCWTKWYSLKPKCPF |
| BHL-bombinin | GIGGALLSFGKSALKGLAKGLAEHF |
| Caerin 1.11 | GLLGAMFKVASKVLPHVVPAITEHF |
| limnonectin-1Fa | SFPFFPPGICKRLKRC |
| G H12 | GLLWHLLHHLLH |
| Plicatamide | FFHLHFH |
| RP557 | RFCWKVCYKGICFKKCK |
| Histatin 5 | DSHAKRHHGYKRKFHEKHHSHRGY |
| Ci-MAM-A24 | WRSLGRTLLRLSHALKPLARRSGW |
| oc_18 | MVLGWGLYICVVAVYGPASWCGVVGGGVV |
| Myticalin B1 | LRWTPTPSYPRYPTRSRGSRWSR |
| BMAP 18 | GRFKRFRKKFKKLFKKLS |
| g6 | MISYRKLAMRVLGHPLRVPTTPPPGPRLTG |
| HsAp1 ( | SGTSEKERESGRLLGVVKRLIVCFRSPFP |
| Brevicillin | SDSVVSDIICTTFCSVTWCQSNCC |
| EA-CATH1 | KRRGSVTTRYQFLMIHLLRPKKLFA |
| MSI-78/Pexiganan | GIGKFLKKAKKFGKAFVKILKK |
| Japonicin-1 | FFPIGVFCKIFKTC |
| Arenicin-1 | RWCVYAYVRVRGVLVRYRRCW |
| Apidaecin IA | GNNRPVYIPQPRPPHPRI |
| oc_17 | MVKKSWIRLSSAKKAAKMRLRPPLTKFFKL |
| Catestatin | SSMKLSFRARAYGFRGPGPQL |
| Panurgine K | LDVKKIICVACKIKPNPACKKICPK |
| B1CTcu1 ( | LIAGLAANFLPKLFCKITK |
| Andersonin-D1 | FIFPKKNIINSLFGR |
| Drosocin | GKPRPYSPRPTSHPRPIRV |
| Alvinellacin | RGCYTRCWKVGRNGRVCMRVCT |
| Phylloseptin-1 | FLSLIPHIVSGVASIAKHF |
| oc_9 | MIDQARKAPTRKMSKEERKKFLDVWHYNDK |
| Cryptonin | GLLNGLALRLGKRALKKIIKRLCR |
| Temporin-GHd | FLQHIIGALSHFF |
| Vasoactive intestinal polypeptide | HSDAVFTDNYTRLRKQMAVKKYLNSILN |
| Nukacin ISK-1 | KKKSGVIPTVSHDCHMNSFQFVFTCCS |
| attacin | DTPFFKSSWEPSTSFSFSKYF |
| Bombinin | GIGALSAKGALKGLAKGLAEHFAN |
| HD-5(1-13) | ATCYCRTGRCATR |
| Cyanophlyctin | FLNALKNFAKTAGKRLKSLLN |
| git_23 | MVGMKGGFTDGKNSRETFRDRKDSACDRR |
| Mucroporin-M1 | LFRLIKSLIKRLVSAFK |
| Melimine | TLISWIKNKRKQRPRVSRRRRRRGGRRRR |
| Peptide-G2 | LVKDNPLDISPKQVQALCTDLVIRCMCCC |
| C-SbGPRP1 | GHGGHGVFGGGYGHGGYGHGYGG |
| oc_27 | MWMSVEHQVQQAREQMELQHLHMELINRL |
| Allomyrinasin | AAVTRRILCWFA |
| Gaegurin-LK1 | FIGPVLKMATSILPTAICKGFKKC |
| Oncopeltus antibacte-rial peptide 4 | VDKPPYLPRPPPPRRIYNNR |
| Capistruin | GTPGFQTPDARVISRFGFN |
| RTD-1 (RTD 1; rhesus theta defensin 1 | GFCRCLCRRGVCRCICTR |
| delta-lysin I | MAADIISTIGDLVKLIINTVKKFQK |
| Hs02 | KWAVRIIRKFIKGFIS |
| Magainin II | GIGKFLHSAGKFGKAFVGEIMKS |
| oc_24 | MLGGVVGGVVGGVVGGVLGGVVGGVVGGV |
| PGLa-St2 ( | GMATKAGTAFGKAAKAIIGAAL |
| Marmelittin | FLFSLIPSAISGLISAFKGRRKRDLN |
| Hainanenin 1 | FALGAVTKLLPSLLCMITRKC |
| oc_8 | MGIYIMGGRKLIEKTENPSNVLNVFFEIK |
| Imcroporin | FFSLLPSLIGGLVSAIK |
| Temporin-PF | FLPLIAGLFGKIF |
| Alyteserin-1a | GLKDIFKAGLGSLVKGIAAHVAN |
| Cacaoidin ( | SSAPCTIYASVSASISATASWGC |
| Lariatin A | GSQLVYREWVGHSNVIKP |
| Con10 | FWSFLVKAASKILPSLIGGGDDNKSSS |
| NKL-24 | IKNQLAIVCDGIGFLKSLCRWFVN |
| Styelin A | GFGKAFHSVSNFAKKHKTA |
| BMAP-27 | GRFKRFRKKFKKLFKKLSPVIPLLHLG |
| oc_12 | MPIINFAKGASRTGSVSHTGKIFGKVYTAK |
| Japonicin-2LF | FIVPSIFLLKKAFCIALKKC |
| CPF-P2 | GLASFLGKALKAGLKIGSHLLGGAPQQ |
| XT-1 | GFLGPLLKLAAKGVAKVIPHLIPSRQQ |
| Astacidin 1 | FKVQNQHGQVVKIFHH |
| P3 | VNFKLLSHSLLVTLASHL |
| m2386 ( | DSIRDVSPTFNKIRRWFDGLFK |
| Caerin 1.10 | GLLSVLGSVAKHVLPHVVPVIAEKL |
| Metchnikowin | HRHQGPIFDTRPSPFNPNQPRPGPIY |
| git_26 | MELKVAGHDDRLFSSNNDYYFDVFSDKGFH |
| Pln149 | YSLQMGATAIKQVKKLFKKKGG |
| Im5 | FLGSLFSIGSKLLPGVIKLFQRKKQ |
| P. sarana Hepcidin | QSHLSLCRYCCNCCRNKGCGYCCKF |
| dicentracin-like peptide | FLRSLLRGAKAIYRGARAGWRG |
| Calcitermin C | VAIALKAAHYHTHKE |
| Human Calcitermin | VAIALKAAHYHTHKE |
| Cytolysin | TTPACFTIGLGVGALFSAKFC |
| DRP-AC4 | SLLSTLGNMAKAAGRAALNAITGLVNQ |
| Delta-Myrtoxin-Mp1a | IDWKKVDWKKVSKKTCKVMLKACKFLG |
| CM15 ( | KWKLFKKIGAVLKVL |
| git_9 | MAGYKKQHTDGPNSEDKALDLFAEMMILC |
| Tolworthcin 524 | DWTCWSCLVCAACS |
| Cinnamycin | CRQSCSFGPFTFVCDGNTK |
| Temporin-GHc | FLQHIIGALTHIF |
| Bactenecin | RLCRIVVIRVCR |
| Maculatin 1.1 | GLFVGVLAKVAAHVVPAIAEHF |
| Uperin 2.1 | GIVDFAKKVVGGIRNALGI |
| Pleurain-a1-thel | RILTMTKRVKMPQLYKQIVCRLFKTC |
| XPF-SE1 ( | GLFLDTLKKFAKAGMEAVINPK |
| NRC-18 | GWKKWFTKGERLSQRHFA |
| Caerin 1.1 | GLLSVLGSVAKHVLPHVVPVIAEHL |
| Hylin-Pul3 | FLGALIPAIAGAIGGLIRK |
| Holothuroidin 1 | HLGHHALDHLLK |
| ToAP1 | FIGMIPGLIGGLISAFK |
| Nigroain-B1 | CVISAGWNHKIRCKLTGNC |
| Maximin 1 | GIGGKILSGLKTALKGAAKELASTYLH |
| Granuliberin-SSa | FIFLPIFRRPVS |
| PMAP-23 | RIIDLLWRVRRPQKPKFVTVWVR |
| Cyclic dodecapeptide | RICRIIFLRVCR |
| Bmkb1 | FLFSLIPSAISGLISAFK |
| puro b5 | FKVTWKTKWWKG-NH2 |
| oc_7 | MAYLREQNVDIIFEKVKELQKFERKRMEKF |
| Brevinin-GR23 | GVTFNALKGVAKTVAAQLLKTAR |
| git_1 | MIGIGWYKKSKNDKKIFQYMKEQNNFEKKH |
| Pleurain-A1 | SIITMTKEAKLPQLWKQIACRLYNTC |
| CPF-AM4 | GLGSLVGNALRIGAKLL |
| Pep39 | RLFRHAFKAVLRL |
| NRC-9 | FFRLLFHGVHHGGGYLNAA |
| Human α-defensin 1 | DCYCRIPACIAGEKKYGTCIYQGKLWAFCC |
| Warnericin RK | MQFITDLIKKAVDFFKGLFGNK |
| Bombinin H2 | LIGPVLGLVGSALGGLLKKI |
| Tricholongin BI | AGFAAQAAASLAPVAAQQL |
| Buffalo hepcidin | DTHFPICIFCCGCCRKGTCGMCCRT |
| Shuchin 3 | KAYSMPRCKGGFRAVMCWL |
| Gomesin | QCRRLCYKQRCVTYCRGR |
| Um2 | ISQSDAILSAIWSGIKSLF |
| **Apidaecin IB** | GNNRPVYIPQPRPPHPRL |
| Apidaecin IB | GNNRPVYIPQPRPPHPRL |
| git_3 | MKTIVKVYLKDEHGNKDWFVTPINLPECRY |
| UyCT1 | GFWGKLWEGVKNAI |
| Eryngin | ATRVVYCNRRSGSVVGGDDTVYYEG |
| CPF-SE1 | GFLGPLLKLGLKGVAKVIPHLIPSRQQ |
| Jingdongin-1-GN1 | FLPLFLPKIICEITKKC |
| mini-ChBac7.5Nalpha | RRLRPRRPRLPRPRPRPRPRPR |
| Kassiniatuerin-3 | FIQHLIPLIPHAIQGIKDIF |
| Nigroain-C2 | FKTWKRPPFQTSCWGIIKE |
| dermaseptin-PT9 (DPT9 | GLWSKIKKAAKTAGKAALGFVNKMV |
| StCT1 | GFWGSLWEGVKSVV |
| mini-ChBac7.5Nbeta | RRLRPRRPRLPRPRPRPRPRP |
| Haloduracin | CAWYNISCRLGNKGAYCTLTVECMPSCN |
| SA-hepcidin1 | QSHLSMCRYCCNCCRNNKGCGFCCKF |
| Hc-CATH | KFFKRLLKSVRRAVKKFRKKPRLIGLSTLL |
| Lasioglossin LL-I | VNWKKVLGKIIKVAK |
| Phylloseptin-H1 | FLSLIPHAINAVSAIAKHN |
| AaeAP1 | FLFSLIPSVIAGLVSAIRN |
| MeuFSPL-2 | FLFSLIPSAISGLINAFK |
| Peptide 5 | VGAVAFGPVGAVVGGLASGFTGKQT |
| Dybowskin-1 | FLIGMTHGLICLISRKC |
| git_25 | MWMEKLQQIRLLQKIIMEQILLQKENMVL |
| XPF (xenopsin precursor fragment | GWASKIGQTLGKIAKVGLKELIQPK |
| Xac-1 | GFVALLKKLPLILKHLH |
| Bac-GM100 | DWTFANWSCLVCDDCSVNLTV |
| Silkworm 001 | YGQSTHAVIYAQGYTYSSDWR |
| FGG(398–413) | YSLKKTSMKIIPFTRL |
| Sm-Piscidin | KGARQAWKDYKYNRNMQKMNQGYGQQGG |
| Epidermin | IASKFICTPGCAKTGSFNSYCC |
| puro b3 | FRVTWRTKWWKG-NH2 |
| XPF-AN1 | GWASKIGQTLGKMAKVGLQELIQPK |
| Salivaricin 9 | GNGVVLTLTHECNLATWTKKLKCC |
| CZS-2 | GFLDVIKHVGKAALGVVTHLINQ |
| oc_16 | MSVVRLEPTSKRRATVFFLNEELKLKMKE |
| Lactoferricin B | FKCRRWQWRMKKLGAPSITCVRRAF |
| Ib-AMP3 | QYRHRCCAWGPGRKYCKRWC |
| IsCT2 | IFGAIWNGIKSLF |
| Jingdongin-1 | FLPLFLPKIICVITKKC |
| Ranalexin | FLGGLIKIVPAMICAVTKKC |
| Andersonin-Y2 | FLPKLFAGIISKNF |
| Neurotensin | QLYENKPRRPYIL |
| Peptide PGQ | GVLSNVIGYLKKLGTGALNAVLKQ |
| Pseudin-1 | GLNTLKKVFQGLHEAIKLINNHVQ |
| Phylloseptin-Co | FLSMIPKIAGGIASLVKNL |
| AJHbalpha | FAHWPDLGPGSPSVKKHGKVIM |
| Uy192 | FLSTIWNGIKGLL |
| Thanatin | GSKKPVPIIYCNRRTGKCQRM |
| Temporin-Cpa | IPPFIKKVLTTVF |
| Salivaricin A | KRGSGWIATITDDCPNSVFVCC |
| LP1A | QRFSQPTFKLPQGRLTLSRKF |
| Human KS-27 | KSKEKIGKEFKRIVQRIKDFLRNLVPR |
| Phylloseptin-PHa | FLSLIPAAISAVSALANHF |
| Amolopin P2 | NVLSSVANGINRALSFFG |
| CAMA | KWKLFKKIGIGKFLHSAKKF |
| DRS-CA-1 | ALWKDLLKNVGKAAGKAVLNKVTDMVNQ |
| Plasticin-B1 | GLVTSLIKGAGKLLGGLFGSVTGGQS |
| Kassinatuerin-2Ma | FLGAIAAALPHVINAVTNAL |
| human MUC7 20-Mer | LAHQKPFIRKSYKCLHKRCR |
| git_15 | MKQGKFKMEGKTKPGNKGFTGHYRTSDFG |
| NCR247 | RNGCIVDPRCPYQQCRRPLYCRRR |
| oc_29 | MGFFSRLTTAKQRNLNGNLNEKVKPKVEER |
| Schmackerin-C1 | AAPRGGKGFFCKLFKDC |
| oc_32 | MALKAKKATKVTKAKLVLKAKKATKVTLV |
| Brevicidine | NYWKKGKWTIGS |
| salivaricin A2 | KRGTGWFATITDDCPNSVFVCC |
| MCF (melittin C-terminal fragment, | GLPALISWIKRKRQQ |
| Hylin-Pul1 | FLGALIPAITGLIGGLINR |
| m2163 | KRKCPKTPFDNTPGAWFAHLILGC |
| Atr-AMP1 | MLCPSYRRCPRVPP |
| Phibilin | RGDILKRWAGHFSKLL |
| NRC-01 | GKGRWLERIGKAGGIIIGGALDHL |
| temporin-shf | FFFLSRIF |
| Microcin J25 | GGAGHVPEYFVGIGTPISFYG |
| Pyrrhocoricin | VDKGSYLPRPTPPRPIYNRN-NH2 |
| Pyrrhocoricin | VDKGSYLPRPTPPRPIYNRN |
| git_10 | MAKILFAKLVNKDYLYRRKALKVNQHEEE |
| Hp1090 | IFKAIWSGIKSLF |
| Microbisporicin A1 ( | VTSWSLCTPGCTSPGGGSNCSFCC |
| StCT2 | GFWGKLWEGVKSAI |
| Spinigerin | HVDKKVADKVLLLKQLRIMRLLTRL |
| B. pascuorum apidaecin | GNRPVYIPPPRPPHPRL |
| Pd_mastoparan PDD-A | INWKKIFEKVKNLV |
| Halocidin | WLNALLHHGLNCAKGVLA |
| LL-23 | LLGDFFRKSKEKIGKEFKRIVQR |
| Bi-bombolitin | LNLKKILGKIGVMLSHLN |
| oc_42 | MLSANRGGSRLPTHGSTGRGGGNGGGGNGH |
| Circulin-A | GIPCGESCVWIPCISAALGCSCKNKVCYRN |
| Acidocin J1132 | NPKVAHCASQIGRSTAWGAVSGA |
| oc_43 | MLSANRVGSRLPTHGSTGRGGGNGGGGNGH |
| Duramycin | CKQSCSFGPFTFVCDGNTK |
| UyCT3 | ILSAIWSGIKSLF |
| BsaA2 | ITSHSLCTPGCAKTGSFNSFCC |
| AMP-IBP5 | AVYLPNCDRKGFYKRKQCKPSR |
| Michiganin A | SSSGWLCTLTIECGTIICACR |
| LMW peptide | APVPFSCTRGCLTHLV |
| pLR (peptide leucine arginine | LVRGCWTKSYPPKPCFVR |
| Melectin | GFLSILKKVLPKVMAHMK |
| GSS extract derived AMP | RHPDYSVALLLR |
| Kassinatuerin-1 | GFMKYIGPLIPHAVKAISDLI |
| PGLa-MW1 | GMASKAGSVLGKITKIALGAL |
| ToAP3 | FIGMIPGLIGGLISAIK |
| Cruzioseptin-16 | GFLDVLKGVGKAALGAVTHLINQ |
| Peptide 19347_2 | RPILIRVRRIRVI |
| Cypemycin | ATPATPTVAQFVIQGSTICLVC |
| Lycocitin 1 | GKLQAFLAKMKEIAAQTL |
| Halictine 1 ( | GMWSKILGHLIR |
| Panitide L2 | QLPICGETCVLGGCYTPNCRCQYPICVR |
| Medusin-PH | LLGMIPVAISAISALSKL |
| oc_5 | MAARYRKRNGSKQGRSRATPRADEVPKFK |
| VmCT1 | FLGALWNVAKSVF |
| git_13 | MIFRDKSKGKGKSKRRKERRQENVFRKER |
| Hymenochirin-1B | IKLSPETKDNLKKVLKGAIKGAIAVAKMV |
| Holothuroidin 2 | ASHLGHHALDHLLK |
| DJK5 | VQWRAIRVRVIR |
| P-02 | GKGLEVIKWKLKHVIQL |
| g1 | KKHKGHKMATHKRKKRLRKNRRKKK |
| Labaditin | VWTVWGTIAG |
| Caeridin-a1 | GLFDIVGKLLGGLGL |
| Distinctin | NLVSGLIEARKYLEQLHRKLKNCKV |
| Saha-CATH5 | KRIGLIRLIGKILRGLRRLG |
| oc_13 | MPSGKKRKRHKVANHKRKKRARANRHKKKK |
| Um4 | FFSALLSGIKSLF |
| Formicin | CEWYNISCQLGNKGQWCTLTKECQRSCK |
| g10 | MRALGYDLAFRWDEPGEGGRQAAMRYKTCC |
| Eumenitin | LNLKGIFKKVASLLT |
| Hylaranin-L1 | GVLSAFKNALPGIMKIIV |
| Pictuseptin-1 | GFLDTLKNIGKTVGRIALNVLT |
| Pilosulin 3 | LIGLVSKGTCVLVKTVCKKVLKQ |
| Alpha-MSH | SYSMEHFRWGKPV |
| git_17 | MNIAPRIRTGGSDGDGKDRFGHDVWAKSY |
| oc_44 | MSIFAFRYNERLEININKEQKQNDESRYHQ |
| RANATUERIN 1 | SMLSVLKNLGKVGLGFVACKINKQC |
| Crabrolin | FLPLILRKIVTAL |
| Xac-2 ( | GFVALLKKLPLILKHLP |
| Ubonodin | GGDGSIAEYFNRPMHIHDWQIMDSGYYG |
| Signiferin 2.1 | IIGHLIKTALGMLGL |
| Palustrin-2ISc | GFMDTAKNVAKNVAATLLDKLKCKITGGC |
| Piceain 1 | KSLRPRCWIKIKFRCKSLKF |
| HNr | VVYTLKRNGRTLYGF |
| Odorranain-HP | GLLRASSVWGRKYYVDLAGCAKA |
| cationic peptide 1037 | KRFRIRVRV |
| The K4 peptide | KKKKPLFGLFFGLF |
| Actagardine | SSGWVCTLTIECGTVICAC |
| Polydim-I | AVAGEKLWLLPHLLKMLLTPTP |
| Cerecidin A1 | TTPLCVGVIIGLTTSIKICK |
| Hp1404 | GILGKLWEGVKSIF |
| Peptide 9 | FPPPGESAVDMSFFYALSNP |
| oc_28 | MEKMKRRAGRLKGRGKRGKHGSPEGEGEEE |
| Kenojeinin I ( | GKQYFPKVGGRLSGKAPLAAKTHRRLKP |
| Caerin 2.6 | GLVSSIGKVLGGLLADVVKSKGQPA |
| oc_15 | MSKYWNGHGGKGGTIKDAKKDNEELRKKQ |
| Scolopin 1 | FLPKMSTKLRVPYRRGTKDYH |
| Hyicin 3682 | ITSFSLCTPGCAKTGSFNSYCC |
| Antapin | GLLSALRKMIPHILSHIKK |
| Peptide 12530 | KFKKVIWKSFL |
| Panurgine 1 | LNWGAILKHIIK |
| Kassorin S | FLGGILNTITGLL |
| Temporin 1OLa | FLPFLKSILGKIL |
| oc_22 | MPSGKKRKRHKMATHKRKKRLKKNRHKKKK |
| Plantazolicin A | RCTCTTIISSSSTF |
| Sviceucin | CVWGGDCTDFLGCGTAWICV |
| Pantinin-1 | GILGKLWEGFKSIV |
| oc_31 | MAGVKDTLKRKLDNTFKMRYNNEKVKIIV |
| H4-(86-100) | VVYALKRNGRTLYGF |
| PDC213 | VTQPLAPVHNPISV |
| CPF-RP-F1 | GFGSVLGKALKFGANLL |
| HP 2-20 | AKKVFKRLEKLFSKIQNDK |
| p138c | GLEETVYIYGANMAS |
| Stigmurin | FFSLIPSLVGGLISAFK |
| Alamethicin | PAAAAQAVAGLAPVAAEQ |
| Hymenochirin-1Pa | LKLSPKTKDTLKKVLKGAIKGAIAIASMA |
| Salmocidin 2A | SGFVLKGYTKTSQ |
| Tigerinin 1 | FCTMIPIPRCY |
| Pelteobagrin | GKLNLFLSRLEILKLFVGAL |
| Lunatusin | KTCENLADTFRGPCFATSNC |
| Temporin-1CEh | FVDLKKIANILNSIF |
| Spiniferin | ILGEIWKGIKDIL |
| oc_3 | MPNGKKKKGHKMATHKRKKRLKKNRHKKKK |
| Pleskein-1 | FFPLIPGVRCKILRTC |
| BacFL31 | GLEESPGHPGQPGPPGPPGAPGP |
| Brevinin-1GHa | FLGAVLKVAGKLVPAAICKISKKC |
| Protonectin | ILGTILGLLKGL |
| Peptide 19347_1 | LIVLTCRKKKKPF |
| AN5-2 | FCKSLPLPLSVK |
| git_12 | MGSVIKKRRKRMSKKKHRKLLRKTRHQRK |
| CZS-1 | GFLDIVKGVGKVALGAVSKLF |
| BING | IRIILRAQGALKI |
| 1018 | VRLIVAVRIWRR-NH2 |
| VK10 | ARTKQTARKSTGGKAPRKQLAT |
| msHemerycin | SVEIPKPFKWNDSF |
| Lt-MAP2 | LIKKLKEYLKKLI |
| temporin A | FLPLIGRVLSGIL |
| Bicarinalin | KIKIPWGKVKDFLVGGMKAV |
| oc_36 | MIDSKGKIMADRTTKYMMLRPVHPRLDSM |
| git_20 | MRMRERMNENKNENKNKNDRENENEIKRE |
| P3-Hp-1891 | RLGTALPALLKTLLAGLNG |
| Jindongenin-1a | DSMGAVKLAKLLIDKMKCEVTKAC |
| Gramicidin S | VKLFPVKLFP |
| Laticeptin | GVVDILKGAAKDLAGHLATKVMNKL |
| oc_33 | MAVKKKKIVISKLNRNNNHKHSEKRKRNK |
| t-DPH1 | GLWSKIKNVAAAAGKAALGAL |
| Tridecaptin A1 | VKGSWSKKFEVIA |
| Peptide 5 | SPPSEQLGKSFNF |
| Peptide 536_2 | GFIVKRFKILV |
| Peptide 536_2 | GFIVKRFKILV |
| Cyclic L27-11 | TWLKKRRWKKAKPP |
| Formaecin 2 | GRPNPVNTKPTPYPRL |
| Scolopendin 2 | AGLQFPVGRIGRLLRK |
| IsCT ( | ILGKIWEGIKSLF |
| Lactocin 63 | RQQPMTLDYRW |
| BjRPS23(67-84) | RKCVRVQLIKNGKKITAF |
| Alyteserin-2Ma | FIGKLISAASGLLSHL |
| Peptide 2 | SPPNQPSIMTFDYAKTNK |
| oc_14 | MPSGKKRKRHKVATHKRKKRARANRHKKKK |
| PGLa-AM1 | GMASKAGSVLGKVAKVALKAAL |
| EC-hepcidin3 | APAKCTPYCYPTHDGVFCGVRCDFQ |
| Uy17 | ILSAIWSGIKGLL |
| BmKn1 | FIGAVAGLLSKIF |
| Fallaxidin 3.1 | GLLDLAKHVIGIASKL |
| Signiferin 2.2 | IIGHLIKTALGFLGL |
| Planosporicin | ITSVSWCTPGCTSEGGGSGCSHCC |
| P-03 | LFAKINGLKVGPLKIQIV |
| Kassporin-KS1 | FLALALIQEAIAKLK |
| BTD-1 (Baboon theta-defensin-1; | GFCRCVCRRGVCRCVCTR |
| Meucin-13 | IFGAIAGLLKNIF |
| Peptide 3967 | FRIMRILRVLKL |
| g8 | MNIKKNLYSQPKSVSTPKADDYMSSYGEK |
| Chromacin | YPGPQAKEDSEGPSQGPASREK |
| git_24 | MVITLSKIPKAKSDGDWKEMQAPLKISVE |
| oc_35 | MIATDSVVMNSKTAPDRKATRKVDMVVRR |
| Aurein 2.2 | GLFDIVKKVVGALGSL |
| Formaecin 1 | GRPNPVNNKPTPHPRL |
| VESP-VB1 ( | FMPIIGRLMSGSL |
| PGLa-SE1 | GMATKAGTALGKVAKAVIGAAL |
| Champacyclin | KIIFLIAI |
| Thuricin S | DWTAWSALVAAACSVELL |
| CPF-PG1 | GFGSLLGKALKIGTNLL |
| Ctri9594 | GVVDTLKNLLMGLL |
| Lysocin E | TRSGFLREQWIT |
| Misgurin | RQRVEELSKFSKKGAAARRRK |
| CPF-ST3 | GLLGPLLKIAAKVGSNLL |
| PGLa-AN2 | GMASKAGSVLGKLAKVAIGAL |
| KN2 (G3K- A4R) | FIKAIARLLRKIF |
| Sclerosin | TPALAVVTTVLPAAAVTTAKSV |
| RP6 | FFQELKEGWKNIKKA |
| oc_39 | MINIPADAQRPAGPDPHEDADRAIFEGRRS |
| HYL | GIMSSLMKKLAAHIAK |
| Lactoferricin B (M4) | CKCRRCQIRCKKPGAPSITCIRRAK |
| Dominulin A | INWKKIAEVGGKILSSL |
| AS-hepc2 | SPAGCRFCCGCCPNMRGCGVCCRF |
| oc_11 | MKAIGRVEVPQEAFVAALGADQPTGRDKK |
| Thaulin-1 | NGNLLGGLLRPVLGVVKGLTGGLGKK |
| Aurein 2.1 ( | GLLDIVKKVVGAFGSL |
| HD-5(1-28) | ATCYCRTGRCATRESLSGVCEISGRLYR |
| Peptide-2 | FLGALGNALSRVL |
| NRWC | NRWCFAGDD |
| Pictuseptin-2 | GFLDTLKNIGKTVGGIALNVLT |
| Peoriaerin IBSD35 | AAGIQAQAGFGLSDSIQGTGKQKCSFCK |
| Odorranain-B1 | AALKGCWTKSIPPKPCFGKR |
| BTD-2 (Baboon theta-defensin-2 | GVCRCVCRRGVCRCVCRR |
| CPF-C1 | GFGSLLGKALRLGANVL |
| RTD-5 (rhesus macaque theta-defensin-5 | GICRCLCRRGVCRCICVL |
| OdVP2 | ILGIITSLLKSLGKK |
| git_6 | MVEMYIGDKESIAYPGGRRKNASDGWKGLS |
| Macropin 1 | GFGMALKLLKKVL |
| human KR-20 | KRIVQRIKDFLRNLVPRTES |
| Peptide 8361_1 | CLIMKVRRKK |
| Casecidin 17 | YQEPVLGPVRGPFPIIV |
| GP-19 ( | GPVGLLSSPGSLPPVGGAP |
| Pth-St1 | RNCESLSHRFKGPCTRDSN |
| git_19 | MPNGKKHKRHKMATHKRKKRLRKNRHKKK |
| g9 | MPNGKKHKRHKMATHKRKKRLRKNRHKKK |
| Lassomycin | GLRRLFADQLVGRRNI |
| Esculentin-1A | GIFSKLAGKKIKNLLISGLKG |
| Mastoparan-X | INWKGIAAMAKKLL |
| git_2 | MKILGKLFRKKKTGYLTMSNKPTKKRKKAR |
| git_27 | MGIKRRYNREEKKSGYKEKIKREDIKVNIR |
| Durancin L28-1A | ENDHRMPYELNRPNNLSKGGAKCGAAIA |
| PFQa-St2 | FIGALLRPALKLLAGK |
| pCM19 | CLRIGMRGRELMGGIGKTM |
| Urechistachykinin I | LRQSQFVGSR |
| Temporin-1CEb | ILPILSLIGGLLGK |
| air_1 | MKNNDRETTHREHKHSGNPAKHKDKKGPS |
| Cc-LTP2 | ITCQQVTAELEPCVPYLTQGIP |
| oc_2 | MKKKKKEKIEEIRERIRLRLEEKKEKKEVE |
| HD-5(7-32) | TGRCATRESLSGVCEISGRLYRLCCR |
| PGLa ( | GMASKAGAIAGKIAKVALKAL |
| P1-Hp-1971 | TKPTLLGLPLGAGPAAGPGKR |
| oc_37 | MILVATDLSKTKKSRTYALDLGDYEKEKF |
| Substance P | RPKPQQFFGLM |
| Mastoparan B | LKLKSIVSWAKKVL |
| Scolopendrasin II | KYALMKKIAELIPNLKSRQVK |
| Lasiocepsin | GLPRKILCAIAKKKGKCKGPLKLVCKC |
| Cm38 | ARDGYIVDEKGCKFACFIN |
| Picturin-1 ( | GVFKDALKQLGAALLDKAANALKPK |
| oc_23 | MKKQEQIQQKLEELENEVKLLWLKIKKEK |
| Hylain 1 | GILDAIKAFANALG |
| git_21 | MVEQEREVERVEWVDVVEQEREEKVEENE |
| RP-1 | ALYKKFKKKLLKSLKRLG |
| Tryglysin A | VNSWGKH |
| git_5 | MSKKNKENRNNNNNNSQNEERQNNNNKNSK |
| NAI-802 | ASSGWVCTLTIECGTVICACR |
| Jelleine -I | PFKISIHL |
| KDAMP | RAIGGGLSSVGGGSSTIKY |
| KAMP-19 | RAIGGGLSSVGGGSSTIKY |
| Mersacidin | CTFTLPGGGGVCTLTSECIC |
| Medipeptin A | TPAAAVVTTVISGAAVTTAKSI |
| Dahlein 1.1 | GLFDIIKNIVSTL |
| Aurein 1.2 | GLFDIIKKIAESF |
| PE1 | KIKFLKVLT |
| Decoralin | SLLSLIRKLIT |
| a14 | GAGLGLSHHFG |
| oc_41 | MKKEEIIEILKLMKEILNDNQINKEEVKKK |
| HD-5(1-9) | ATCYCRTGR |
| Chaxapeptin | GFGSKPLDSFGLNFF |
| PN5 | FKFLARTGKFL |
| PN5 | FKFLARTGKFL |
| oc_6 | MATAMKCSSGAKKASPKTSPNPALIHKPRP |
| lacrain | RYPAVGYT |
| B2RP-ERa ( | GVIKSVLKGVAKTVALGML |
| Seg-5L | KKKLLLLLLLLLKKK |
| Seg5D | KKKLLLLLLLLLKKK |
| Griselimycin | VPSLPLVPLG |
| Secretolytin | QKIAEKFSGTRRG |
| oc_20 | MITYAKELNFEKAAEIRDKIKELEKILEK |
| D-K6L9 | LKLLKKLLKKLLKLL |
| Peptide AN5-1 | YSKSLPLSVLNP |
| Leptoglycin | GLLGGLLGPLLGGGGGGGGGLL |
| Shuchin 2 | NALSSPRNKCDRASSCFG |
| Mel 4 | KNKRKRRRRRRGGRRRR |
| Citropin 1.1 | GLFDVIKKVASVIGGL |
| Citropin 1.1 | GLFDVIKKVASVIGGL |
| Histone H2A | AERVGAGAPVYL |
| Lugdunin | CVWLVVV |
| TRP1-TINF | GPSGFLGMR |
| Teixobactin | FISQIISTARI |
| CPF-AM1 (caerulein precursor fragment-AM1 | GLGSVLGKALKIGANLL |
| Riparin 2.1 | IIEKLVNTALGLLSGL |
| So-D1 | TCESPSHKFKGPCATNRNCES |
| oc_10 | MITADEMKKLAFEKKEELSEDNINIEKLIM |
| Pinipesin | VAEARQGSFSY |
| Peptide 8 | APPPGYAMESDSFS |
| U-VVTX-Vm1e | INLRAIAAFAKKLL |
| Ocellatin-P1 | GLLDTLKGAAKNVVGSLASKVMEKL |
| g4 | MASRPPEGKEGKKRKEKKFDGIRHRELQKK |
| Ocellatin-S1 | GVLDILKGAAKDLAGHVATKVINKI |
| oc_21 | MIRGIEDDTTDALTLRHRGSDRKEEDHEDR |
| P1-Ll-1577 | DEMKLDGFNMHLE |
| Seg6L | LLLLLKKKKKKLLLL-NH2 |
| Seg6D | LLLLLKKKKKKLLLL |
| Balteatide | LRPAILVRIK |
| oc_30 | MIEPRAGKNQGKMIPKAGSRSKESDGGMKL |
| oc_38 | MKKIRNRGIREKPPKRGEKGGKAPPAGRA |
| skin_3 | MSQGHCVELRTQAAKEERKRERERERERE |
| cAMP70 | ANAIIGVIASLSPKARNNTNCCTL |
| Nigrocin-HLM | GLLSGILGAGKKIVF |
| Polybia-MP-II | INWLKLGKMVIDAL |
| Polybia-MP-II | INWLKLGKMVIDAL |
| Lt-MAP3 | LAKKLAKYLKKAL |
| Micrococcin P1 | SCTTCVCTCSCCTT |
| LFX01 | ITGGPAVVHQA |
| LFX01 | ITGGPAVVHQA |
| Amurin-2b | FLPLLLSALPSFLCLVFKKC |
| Eumenine mastoparan-AF | INLLKIAKGIIKSL |
| Feleucin-BO1 | FLGLLGSLL |
| Ocellatin-F1 | GVVDILKGAAKDIAGHLASKVMNKL |
| Guentherin | VIDDLKKVAKKVRRELLCKKHHKKLN |
| Oligoventin | QPFSLERW |
| Plantaricin SLG1 | YGNGVFSVIK |
| Myxinidin | GIHDILKYGKPS |
| Jelleine-III | EPFKISIHL-NH2 |
| git_7 | MVNNVICLKRKTKDGEDSKYRKGDGGFHDG |
| HRNR1132–1143 | GSGSRQSPSYGR |
| Jelleine-IV | TPFKISIH-NH2 |
| git_22 | MVGIEGGFTDGKNSRETFRDRKDSACDRR |
| VCP-VT2 | FLPIIGKLLSG |
| Jelleine-II | TPFKISIHL-NH2 |
| Dendropsophin 1 | NLLNDALGTVNGLLGRS |
| Acidocin LCHV | NVGVLNPPPLV |
| MP-VB1 | INMKASAAVAKKLL |
| skin_1 | MHCCCLCEEEEEEEEEEEEEEEEEEEEEE |
| Polybia-CP | ILGTILGLLKSL |
| cAMP533 (cAMP_533; Predicted, gut microbiome, UCLL1) | GKGIKFVGEEIRRKSGKSAGAK |
| ASP-1 | FTVATFI |
| Nigrocin-PN | GLLGKILGAGKKVLCGVSGLC |
| VK7 | AKKPVAKKAAGGVKKPK |
| Histone H6-like protein | PKRKSATKGDEPA |
| Wollamide A | WLLINK |
| Brucin | HTLCMDGGATY |
| Lichenin | ISLEICAIFHDN |
| Paenibacterin | KVTKSVKSIPVKI |
| RP9 | RGSALTHLP |
| puro b2 | FPVTWRTKWWKG-NH2 |
| Mastoparan M | INLKAIAALAKKLL |
| GL13K | GKIIKLKASLKLL |
| GL13K | GKIIKLKASLKLL |
| TP | ASVVNKLTGGVAGLLK |
| VK25 | SPKKTKPVKPKKVA |
| Mastoparan-VT2 | NLKAIAALAKKLL |
| Cr-ACP1 | AWKLFDDGV |
| U-VVTX-Vm1b | INLRAIAALAKKLL |
| Parkerin (M2) | GKAKTKKIKAGGKCKITGAA |
| Mastoparan-C | LNLKALLAVAKKIL |
| P-10 | VSKIKKYLKYKDRI |
| VK6 | AVKPKTAKPKTAKPKTA |
| Enterocin LD3 | QGGQANQ |
| skin_2 | MIVICCDYDEDDNENDDDDDDDDDDDDNF |
| Taromycin A | WNDTGKDADGAEY |
| Anoplin | GLLKRIKTLL |
| Parasin I | KGRGKQGGKVRAKAKTRSS |
| Lactocillin | SCTTCTCCCSCCA |
| P2-Ll-1298 | AAGKGLVSNLLEK |
| Andricin 01 | AIGHCLGATL |
| Triculamin | SKKSKPGDGIRGKGVRG |
| Crinicepsin-1 | VGVGGGFGR |
| Hylaseptin P1 | GILDAIKAIAKAAG |
| Colistin A | KTKKKLLKKT |
| Polymyxin B | KTKKKFLKKT |
| Dosotamide A | WLLING |
| Desotamide B | WGNVLL |
| Emericellipsin A | PQAAIVASG |
| Cn-AMP1 | SVAGRAQGM |
| Mycosubtilin | NYNQPSN |
| Frenatin 1 | GLLDALSGILGL |
| Leucrocin I | NGVQPKY |
| JCpep7 | KVFLGLK |
| Baceridin | WAIVLL |
| Sesquin | KTCENLADTY |
| A21978C1 | WNDTGKDADGSEY |
| Oncorhyncin I | SKGKKANKDVELARG |
| Paracentrin 1 | EVASFDKSKLK |
| Peptide fraction II | GGGGGGHLVA |
| Delftibactin-A | SDTGTKSRK |
| YD1 | APKGVQGPNG |
| Peptide 7 | APPPGLSAGV |
| Carnocin UI49 | GSEIQPR |
| MDN-0066 | LETLLSLI |
| cOB1 | VAVLVLGA |
| Cathepsin G(1-5) | IIGGR |
| Gageostatin A | ELLVDLL |
| Fusaricidin A | TVVTNA |
| RLID 12.1 | TPPQS |
| EP2 | AMVSS |
| skin_4 | MKKEEEEEEEKDDEDDDEDEKEEKEEEEEM |
| XLAsp-P2 | DEDLDE |
| XLAsp-P1 | DEDDD |
| SAAP fraction 3 | DDDDDDD |
| EP5-1 | ACSAG |

| **DRAMP** | |
| --- | --- |
| **Peptide Name** | **Peptide Sequence** |
| Gageotetrin A (Bacteriocin) | LE |
| Gageotetrin B (Bacteriocin) | LLLE |
| Gageotetrin C (Bacteriocin) | LLLE |
| Coconut antifungal peptide (Plants) | EQCREEEDDR |
| Malanin chain B (Plant defensin) | DETXTDEEFN |
| Fusaricidin A (Bacteriocin) | TVVTNA |
| Fusaricidin B (Bacteriocin) | TVVTQA |
| Chitinase (Bacteriocin; Streptomyces violaceusniger, Bacteria) | GDGTGPGPGP |
| Microcin 7 (Bacteriocin; Escherichia coli, Bacteria) | MRTGNAD |
| JCpep7 (Plants) | KVFLGLK |
| Lantibiotic carnocin-UI49 (Bacteriocin) | GSEIQPR |
| YD1 (bacteriocin) | APKGVQGPNG |
| YD1 (bacteriocin) | APKGVQGPNG |
| Bacteriocin | ASILTNAS |
| PGLa-H (chain of PYLa/PGLa A; Frogs, amphibians, animals) | KIAKVALKAL |
| Antifungal protein J (AFP-J; Plants) | LPSDATLVLDQTGKELDARL |
| Patellamide D(Bacteriocin) | ISVCITVC |
| S. amritsarensis lipopeptide (Bacteriocin) | ATQSHQ |
| S. amritsarensis lipopeptide (Bacteriocin) | ATQSHQ |
| Antifungal lectin AMML (AMML; Plant defensin) | ESGINLQGDATLANN |
| VK12 (histone derived; animals) | AKKPVAKKAAGGVKKPKK |
| Trifolitoxin (TFX; Bacteriocin) | DIGGSRQGCVA |
| Taromycin A (Bacteriocin) | WNDTGKDADGAEY |
| Bioactive peptide 3 (BAP3; Curvalicin-28c; Bacteriocin) | NIPQLTPTP |
| Bioactive peptide 3 (BAP3; Curvalicin-28c; Bacteriocin) | NIPQLTPTP |
| Patellamide A(Bacteriocin) | ITVCISVC |
| BTL (Bacteriocin) | KQQLATEAESAGPIL |
| BTL (Bacteriocin) | KQQLATEAESAGPIL |
| Plantaricin ZJ008 (Bacteriocin) | QWGGG |
| Guentherin (AMP-3; Frogs, amphibians, animals) | VIDDLKKVAKKVRRELLCKKHHKKLN |
| Gageostatin A (Bacteriocin) | LLDVLLE |
| Gageostatin B (Bacteriocin) | LLDVLLE |
| Gageostatin C (Bacteriocin) | LLDVLLE |
| Trichamide(Bacteriocin) | EDGLHPRLCSC |
| Trichamide(Bacteriocin) | EDGLHPRLCSC |
| Microcin C7 (MccC7; Microcin C51, MccC51; Bacteriocin) | MRTGNAN |
| Daptomycin(Bacteriocin) | WNDTGKDADGSEY |
| Daptomycin(Bacteriocin) | WNDTGKDADGSEY |
| Daptomycin(Bacteriocin) | WNDTGKDADGSEY |
| Daptomycin(Bacteriocin) | WNDTGKDADGSEY |
| Trifolitoxin (TFX; Bacteriocin) | DIGGSRQGCVA |
| Anticancerous peptide 1 (Cr-ACP1; Plants) | AWKLFDDGV |
| Fusaricidin D (Bacteriocin) | TVYTQA |
| Fusaricidin C (Bacteriocin) | TVYTNA |
| Rhamnosin A (Bacteriocin) | AVPAVRKTNETLD |
| nocathiacin I(Bacteriocin) | SCTTCECSCSCSS |
| Amythiamicin C/D(Bacteriocin) | SCNCVCGVCCSC |
| Fengycin A2(Bacteriocin) | EKYTEAPEYV |
| Paenibacterin (Bacteriocin) | KVTKSVKSIPVKI |
| Frenatin-1 (Frogs, amphibians, animals) | GLLDALSGILGL |
| Baceridin(Bacteriocin) | WAIVLL |
| Defensin (Insects, animals) | AHCLAIGRK |
| VK12 (histone derived; animals) | AKKPVAKKAAGGVKKPKK |
| Antifungal protein Pr-2 (Pr-2; Plant defensin) | QGIGVGDNDGKRGKR |
| Tn-AFP1 (Trapa natans antifungal peptide; Plant defensin) | LMCTHPLDCSN |
| Uperin-3.6 (toads, amphibians, animals) | GVIDAAKKVVNVLKNLP |
| Odorranain-V1 (OdV1; Frogs, amphibians, animals) | GLLSGTSVRGSI |
| Antimicrobial peptide 1 (Cn-AMP1; Plant defensin) | SVAGRAQGM |
| Hylaseptin-P1 (HSP1) | GILDAIKAIAKAAG |
| Ala-6-fenycin (Bacteriocin) | EKYTEAPEYI |
| Fengycin A(Bacteriocin) | EKYTEAPEYI |
| VK11 (histone derived; reptiles; animals) | AKAVKPKTAKPKTAKPKTAKA |
| Riparin-2.1 (Frogs, amphibians, animals) | IIEKLVNTALGLLSGL |
| NRC-1 (fish, chordates, animals) | GKGRWLERIGKAGGIIIGGALDHL |
| Protein PR-L3 (Plant defensin) | GIFTFEDESTTTVAPAKLYK |
| Mastoparan B (MP-B) | LKLKSIVSWAKKVL |
| Patellamide C(Bacteriocin) | VTACITFC |
| Parasin I (histone-H2A-derived; catfishes, chordates, animals) | KGRGKQGGKVRAKAKTRSS |
| Neurokinin A (NKA; chicken, animals) | HKTDSFVGLM |
| Amythiamicin A/B (Bacteriocin) | SCNCVCGVCCSCSP |
| Putative antimicrobial protein 1 (Ls-AMP1; Plant defensin) | EALYNSEDLYEETSDSDD |
| Dermaseptin derivative K4-S4-(1-13) | ALWKTLLKKVLKA |
| Nigrocin-1-OR1 (Frogs, amphibians, animals) | GLISGILGVGKKLVCGLSGLC |
| Amythiamicin A/B (Bacteriocin) | SCNCVCGVCCSCSP |
| PGLa-AN2 | GMASKAGSVLGKLAKVAIGAL |
| Polybia-MP-II (insects, vertebrates, animals) | IDWLKLGKMVIDAL |
| Nigrocin-2HSb (Frogs, amphibians, animals) | GLLGSIFGAGKKIACALSGLC |
| Listeriolysin S(Bacteriocin) | CCSCSCSTCTCTCTCASSAATKM |
| Del 1-2 (Ranalexin analog) | GGLIKIVPAMICAVTKKC |
| Amaryllin (Plant defensin) | QKIQEIDLQTYLQPQ |
| VK6 (histone derived; reptiles; animals) | AVKPKTAKPKTAKPKTA |
| Bifidin I(Bacteriocin) | KYGDVPLY |
| Nosiheptide (NOS; Antibiotic 9671-RP) | SCTTCECCCSCS |
| Crinicepsin-2 (insects, arthropods, invertebrates, animals) | RERSKGSKYLYVG |
| Antimicrobial peptide 3 (Cn-AMP3; Plant defensin) | TCSYTMEA |
| Putative antimicrobial protein 2 (Ls-AMP2; Plant defensin) | SPPEAAYGPGNTNSDSGDK |
| Antimicrobial protein 1 (Antimicrobial protein AN5-1) | YSKSLPLSVLNP |
| Spinigerin (Insects, animals) | HVDKKVADKVLLLKQLRIMRLLTRL |
| Hlsal-defensin (H. longicornis salivary gland defensin; Ticks, Arthropods, animals; Predicted) | NCIQQCVSKGAQGGYCTNEKCTCY |
| [A6]-IsCT (Mutant: W6A; IsCT analog) | ILGKIAEGIKSLF |
| Halocin-C8 (HalC8; Bacteriocin) | DIDITGCSACKYAAG |
| IMX942 | KSRIVPAIPVSLL |
| [L6, K11]-IsCT (IsCT analog through amino acids substitution) | ILGKILKGIKKLF |
| VK14 (histone derived;reptiles; animals) | KAAAAKKSPKKPKKPAAAKK |
| VK14 (histone derived;reptiles; animals) | KAAAAKKSPKKPKKPAAAKK |
| Nigrocin-2GRb (Frogs, amphibians, animals) | GLFGKILGVGKKVLCGLSGMC |
| Alyteserin-1Mb (toads, amphibians, animals) | GFKEVLKAGLGSLVKGIPAHVAN |
| Nigrocin-2VB (Frogs, amphibians, animals) | SILSGNFGVGKKIVCGLSGLC |
| VK25 (histone derived; reptiles; animals) | SPKKTKPVKPKKVA |
| VK25 (histone derived; reptiles; animals) | SPKKTKPVKPKKVA |
| Bacteriocin serracin-P 43 kDa subunit (Bacteriocin) | DYHHGVRVL |
| Cryptonin (Insects, animals) | GLLNGLALRLGKRALKKIIKRLCR |
| Cucurmoschin (Plants) | PQRGEGGRAGNLLREEQEI |
| Del 1-4 (Ranalexin analog) | LIKIVPAMICAVTKKC |
| Ala4-uperin 3.6 (toads, amphibians, animals) | GVIAAAKKVVNVLKNLF |
| Plantazolicin (PZN; Bacteriocin) | RCTCTTIISSSSTF |
| Plantazolicin (PZN; Bacteriocin) | RCTCTTIISSSSTF |
| Nigrocin-2SCa (Frogs, amphibians, animals) | GILSGILGAGKSLVCGLSGLC |
| Nigrosin-OG13 (Frogs, amphibians, animals) | GLLSGILGAGKHIVCGLSGLR |
| 40S ribosomal protein S30 (Fish, chordates, animals) | KVHGSLARAGK |
| Halocin-C8 (HalC8; Bacteriocin) | DIDITGCSACKYAAG |
| Fengycin B(Bacteriocin) | EKYTEVPEYI |
| Citropin-1.3 (Frogs, amphibians, animals) | GLFDIIKKVASVIGGL |
| Lectin | TGVAWRIT |
| Endochitinase 3 (Plant defensin) | MYDESTGYSSALK |
| VK7 (histone derived; reptiles; animals) | AKKPVAKKAAGGVKKPK |
| Cecropin A (1-7)-melittin (1-8)hybrid peptide | KWKLFKKGIGAVLKV |
| SPX(1-16)(truncated peptide of Syphaxin; Frogs, amphibians, animals) | GVLDILKGAAKDLAGH |
| Temporin-1P (Temporin-1M; Temporin-1CSa; Frogs, amphibians, animals) | FLPIVGKLLSGLL |
| CGA47-60 (hrVS-1-derived peptide) | RILSILRHQNLLKE |
| VK10 (histone derived; reptiles; animals) | ARTKQTARKSTGGKAPRKQLAT |
| Nigrocin-OG5 (Frogs, amphibians, animals) | GLLSGILGAGKQKVCGLSGLC |
| Ala7-uperin 3.6 (toads, amphibians, animals) | GVIDAAAKVVNVLKNLF |
| GLK-19 | GLKKLLGKLLKKLGKLLLK |
| dPSM | VGTIIKIIKAIIDIFAK |
| Nigrosin-OG21 (Frogs, amphibians, animals) | GLLSGVLGVGKKVLCGLSGLC |
| 27 kDa antibacterial protein | GIGGKPVQTAFVDNDGIYD |
| Mersacidin (Bacteriocin; Preclinical) | CTFTLPGGGGVCTLTSECIC |
| Mersacidin | CTFTLPGGGGVCTLTSECIC |
| Mersacidin (Bacteriocin; Preclinical) | CTFTLPGGGGVCTLTSECIC |
| VK11 (histone derived; reptiles; animals) | AKAVKPKTAKPKTAKPKTAKA |
| [K7, P8, K11]-IsCT (IsCT analog through amino acids substitution) | ILGKIWKIKKLF |
| Nigrocin-1-OW1 (Frogs, amphibians, animals) | GILSGVLGMGKKIVCGLSGLC |
| Nigrocin-2JDa (Frogs, amphibians, animals) | GIFGKILGAGKKVLCGLSGLC |
| Pleurocidin-like peptide AP3 (NRC-13; fish, chordates, animals; Predicted) | GWRTLLKKAEVKTVGKLALKHYL |
| Odorranain-H1 (OdH1; Frogs, amphibians, animals) | GIFGKILGVGKKVLCGLSGVC |
| Nigrocin-2GRc (Frogs, amphibians, animals) | GLLSGILGAGKNIVCGLSGLC |
| Vespid chemotactic peptide 5e (VCP 5e; Insects, animals) | FLPIIAKLLGGLL |
| SPX(1-22)(truncated peptide of Syphaxin; Frogs, amphibians, animals) | GVLDILKGAAKDLAGHVATKVI |
| Antimicrobial peptide scolopin-1 | FLPKMSTKLRVPYRRGTKDYH |
| Alyteserin-2a (toads, amphibians, animals) | ILGKLLSTAAGLLSNL |
| LL-23 (Derived from LL-37) | LLGDFFRKSKEKIGKEFKRIVQR |
| Ocellatin-F1 (Fallaxin; Frogs, amphibians, animals) | GVVDILKGAAKDIAGHLASKVMNKL |
| Cecropin A (1-8)-melittin (1-13)hybrid peptide | KWKLFKKIEKVGQGIGAVLKVLTTGL |
| Temporin-1Lc (Temporin 1Lc; Frogs, amphibians, animals) | FLPILINLIHKGLL |
| BacFL31(Bacteriocin) | GLEESPGHPGQPGPPGPPGAPGP |
| Sungsanpin (a class 2 lasso peptide; bacteriocins) | GFGSKPIDSFGLSWL |
| Sungsanpin (a class 2 lasso peptide; bacteriocins) | GFGSKPIDSFGLSWL |
| Nigrocin-2GRa (Frogs, amphibians, animals) | GLLSGILGAGKHIVCGLSGLC |
| H. erythraea B2RP | GVIKSVLKGVAKTVALGML |
| Ranacyclin-E (Frogs, amphibians, animals) | SAPRGCWTKSYPPKPCK |
| Nigrocin-2ISc (Frogs, amphibians, animals) | GILSTVFKAGKGIVCGLSGLC |
| Antifungal peptide (Cm-p1; Plants) | SRSELIVHQR |
| Nigrocin-1-OA2 (Frogs, amphibians, animals) | GIFGKILGVGKKTLCELSGMC |
| Ala14-uperin 3.6 (toads, amphibians, animals) | GVIDAAKKVVNVLANLF |
| Nigrocin-1-OW3 (Frogs, amphibians, animals) | GILGNIVGMGKKVVCGLSGLC |
| GE2270A(Bacteriocin) | SCNCVCGFCCSCSPSA |
| Ranacyclin-B-RN2 (Frogs, amphibians, animals) | SALVGCGTKSYPPKPCFGR |
| Temporin-1La (Temporin 1La; Frogs, amphibians, animals) | VLPLISMALGKLL |
| D16F-N23 (single amino acid substitution) | GILDTLKQFAKGVGKFLVKGAAQ |
| Dermaseptin-H5 (Dermaseptin-like peptide 5, DMS5; Frogs, amphibians, animals) | GLWSTIKNVGKEAAIAAGKAVLGSL |
| Bombinin-H1/H3 (Frogs, amphibians, animals) | IIGPVLGMVGSALGGLLKKI |
| Nigrocin-1-OA1 (Frogs, amphibians, animals) | GLLSGVLGVGKKIVCGLSGLC |
| Alyteserin-1Ma (toads, amphibians, animals) | GFKEVLKADLGSLVKGIAAHVAN |
| Lichenin (Bacteriocin-like) | ISLEICXIFHDN |
| Aurein-1.1 (Frogs, amphibians, animals) | GLFDIIKKIAESI |
| Protein PR-L1 (Plant defensin) | GIFTFEDESTSTVAPAKLYK |
| Amphipathic peptide CT1 (StCT1; Non-disulfide-bridged peptide 5, NDBP-5; Arthropods, animals) | GFWGSLWEGVKSVV |
| Bacteriocin TSU4 (bacteria, prokaryotes) | SMSGFSKPHD |
| Salivaricin 9 (Sal9; Bacteriocin) | GNGVVLTLTHECNLATWTKKLKCC |
| Grammistin Gs B (Group II grammistin; soapfish, chordates, animals) | IGGIISFFKRLF |
| Ocellatin-P1 (Pentadactylin; Frogs, amphibians, animals) | GLLDTLKGAAKNVVGSLASKVMEKL |
| Cecropin A (1-7)-melittin (3-10)hybrid peptide | KWKLFKKGAVLKVLT |
| Protein PR-L5 (Plant defensin) | SIFAFQDESPSAIAQAKLFK |
| Thiocillin (Bacteriocin) | SCTTCVCTCSCCTT |
| Thiocillin (Bacteriocin) | SCTTCVCTCSCCTT |
| M-lycotoxin-Ls3a (M-LCTX-Ls3a; Lycocitin-1; spiders, Arthropods, animals) | GKLQAFLAKMKEIAAQTL |
| Aurein-2.6 (Frogs, amphibians, animals) | GLFDIAKKVIGVIGSL |
| Nigrocin-2SCc (Frogs, amphibians, animals) | GILSNVLGMGKKIVCGLSGLC |
| Frenatin-2 (Frogs, amphibians, animals) | GLLGTLGNLLNGLGL |
| Signiferin-2.1 (Frogs, amphibians, animals) | IIGHLIKTALGMLGL |
| Nigrocin-2ISb (Frogs, amphibians, animals) | GILGTVFKAGKGIVCGLTGLC |
| Antifungal protein 1 (GAFP-1; Plant defensin) | LDSLSFSYNNFEEDD |
| Grammistin PpIIb (Group II grammistin; fish, chordates, animals) | FIGGIISFIKKLF |
| L-amino-acid oxidase (LAAO; LAO; Dactylomelin-P) | DGVCSNRRQCNKEVCGSSYDVAIVGA |
| Nigroain-K1 (Frogs, amphibians, animals) | SLWETIKNAGKGFIQNLDKIR |
| Nigroain-K1 (Frogs, amphibians, animals) | SLWETIKNAGKGFIQNLDKIR |
| Temporin-1Lb (Temporin 1Lb; Frogs, amphibians, animals) | NFLGTLINLAKKIM |
| Polybia-MP-III (insects, vertebrates, animals) | IDWLKLGKMVMDVL |
| Grammistin Pp3 (Group III grammistin; fish, chordates, animals) | NWRKILGQIASVGAGLLGSLLAGYE |
| Kunitz-type serine protease inhibitor 1 (Xb-KTI; Plants) | PVVDTTGNNPLQQQEEYYV |
| Myeloid antimicrobial peptide BMAP-28 (1-18) (mammals, animals) | GGLRSLGRKILRAWKKYG |
| Myeloid antimicrobial peptide BMAP-28 (1-18) (mammals, animals) | GGLRSLGRKILRAWKKYG |
| Venom antimicrobial peptide-6 (Meucin-13; NDBP-5; Arthropods, animals) | IFGAIAGLLKNIF |
| Del 1 (Ranalexin analog) | LGGLIKIVPAMICAVTKKC |
| Antimicrobial protein BL-A60 | LYKLVKVVLNM |
| K7 (Bac2A variant through single amino acid substitution) | RLARIVKIRVAR |
| G18C (truncated isoform of thanatin, residue 1-18) | GSKKPVPIIYCNRRTGKC |
| P2 (Human, mammals, animals) | KAGLAFPVGRVHRLLRK |
| M-lycotoxin-Ls3b (M-LCTX-Ls3b; Lycocitin-2; spiders, Arthropods, animals) | GRLQAFLAKMKEIAAQTL |
| Temporin-A (Frogs, amphibians, animals) | FLPLIGRVLSGIL |
| Citropin-1.1 (Frogs, amphibians, animals) | GLFDVIKKVASVIGGL |
| R11 (Bac2A variant through single amino acid substitution) | RLARIVVIRVRR |
| Malanin chain A (Plant defensin) | DYPKLTFTTS |
| Nigrocin-2HSa (Frogs, amphibians, animals) | GLLGSLFGAGKKVACALSGLC |
| Nigrocin-2JDb (Odorranain-H2; Frogs, amphibians, animals) | GIFGKILGVGKKVLCGLSGMC |
| Lariatins A (lasso peptide; Bacteriocin) | GSQLVYREWVGHSNVIKP |
| Antimicrobial peptide 6 (XT-6; Frogs, amphibians, animals) | GFLGSLLKTGLKVGSNLL |
| Bombinin-H5 (Frogs, amphibians, animals) | IIGPVLGLVGSALGGLLKKI |
| Ranatuerin-4 (Frogs, amphibians, animals) | FLPFIARLAAKVFPSIICSVTKKC |
| Ranatuerin-4 (Frogs, amphibians, animals) | FLPFIARLAAKVFPSIICSVTKKC |
| Leptoglycin (Gly-rich; Frogs, amphibians, animals) | GLLGGLLGPLLGGGGGGGGGLL |
| Antimicrobial peptide 7 (XT-7; Frogs, amphibians, animals) | GLLGPLLKIAAKVGSNLL |
| Polybia-CP (Polybia chemotactic peptide; Venom protein CP; Insects, animals) | ILGTILGLLKSL |
| delta-lysin I (Bacteriocin) | MAADIISTIGDLVKLIINTVKKFQK |
| Nigrocin-OG4 (Frogs, amphibians, animals) | GLLSGILGAGKHIICGLSGLC |
| Deoxyactagardine B(Bacteriocin) | ASGWVCTLTIECGTLVCAC |
| Actagardine B(Bacteriocin) | ASGWVCTLTIECGTLVCAC |
| Pleurostatin (Fungus) | VRPYLVAF |
| Amphiphatic peptide CT2 (VmCT2; Non-disulfide-bridged peptide 5.14, NDBP-5.14; Arthropods, animal | FLSTLWNAAKSIF |
| Y12M (truncated isoform of thanatin, residue 10-21) | YCNRRTGKCQRM |
| PlnA-17 (Bacteriocin) | GATAIKQVKKLFKKWGW |
| Citropin-1.1 sm2 (Frogs, amphibians, animals) | GLFDVIAKVASVIGGL |
| G19Q (truncated isoform of thanatin, residue 1-19) | GSKKPVPIIYCNRRTGKCQ |
| Novispirin T-7 (mutation of Ovispirin-1) | KNLRRITRKIIHIIKKYG |
| Bombinin-like peptide 3 (BLP-3; toads, amphibians, animals) | GIGAAILSAGKSALKGLAKGLAEHF |
| Alyteserin-1c (toads, amphibians, animals) | GLKEIFKAGLGSLVKGIAAHVAS |
| Gaegurin-RN4 (Frogs, amphibians, animals) | FVGPVLKIAAGILPTAICKIYKKC |
| LL-37(17-32)(C-terminal fragment of LL-37; Human, mammals, animals) | FKRIVQRIKDFLRNLV |
| Del 20 (Ranalexin analog) | FLGGLIKIVPAMICAVTKK |
| Vespid chemotactic peptide 5g (VCP 5g; Insects, animals) | FLIIRRPIVLGLL |
| Antimicrobial peptide 2 (Cn-AMP2; Plant defensin) | TESYFVFSVGM |
| Nigrocin-2LVb (Frogs, amphibians, animals) | GILSGILGMGKKLVCGLSGLC |
| Gaegurin-RN5 (Frogs, amphibians, animals) | FLGPIIKIATGILPTAICKFLKKC |
| R5 (Bac2A variant through single amino acid substitution) | RLARRVVIRVAR |
| Citropin-1.2 (Frogs, amphibians, animals) | GLFDIIKKVASVVGGL |
| Odorranain-A-OA1 (Frogs, amphibians, animals) | VVKCSYRLGSPDSQCN |
| Nigrocin-1-OR2 (Frogs, amphibians, animals) | GLISGILGVGKMLVCGLSGLC |
| MSI-78(Pexiganan) | GIGKFLKKAKKFGKAFVKILKK |
| Aurein-1.2 (Frogs, amphibians, animals) | GLFDIIKKIAESF |
| Antimicrobial peptide scolopin-2 | GILKKFMLHRGTKVYKMRTLSKRSH |
| Thuricin 439A,439B(Bacteriocin) | GWVAXVGAXGTAALASGGV |
| Mersacidin (Bacteriocin; Preclinical) | CTFTLPGGGGVCTLTSECIC |
| CGA41-60 (hrVS-1-derived peptide) | TLRGDERILSILRHQNLLKE |
| Grammistin Pp1 (Group II grammistin; fish, chordates, animals) | FIGGIISFFKRLF |
| Crinicepsin-1 (insects, arthropods, invertebrates, animals) | VGVGGGFGR |
| Mucroporin (Antimicrobial peptide 36.21; Arthropods, animals) | LFGLIPSLIGGLVSAFK |
| Cecropin A (1-7)-melittin (5-12)hybrid peptide | KWKLFKKVLKVLTTG |
| PhTD-1 (PhTD1; primates, mammals, animals) | RCVCRRGVCRCVCTRGFC |
| Dermaseptin-like PBN2 (DRP-PBN2; Plasticin-B1a; Frogs, amphibians, animals) | GLVTSLIKGAGKLLGGLFGSVTGGQS |
| NAI-802 (Bacteriocin) | ASSGWVCTLTIECGTVICACR |
| Thiocillin GE37468 (Antibiotic GE37468; Bacteriocin) | STNCFCYICCSCSS |
| Thiocillin GE37468 (Bacteriocin) | STNCFCYICCSCSS |
| Thiocillin GE37468 (Antibiotic GE37468; Bacteriocin) | STNCFCYICCSCSS |
| Thiocillin GE37468 (Bacteriocin) | STNCFCYICCSCSS |
| Plantaricin ZJ5 (Bacteriocin) | KTKQQFLIKAQTQLFKVFGYTL |
| P1 (Human, mammals, animals) | FLAKAVAKAAAKALAKAL |
| Citropin-1.1 sm1 (Frogs, amphibians, animals) | GLFAVIKKVASVIGGL |
| Magainin-2 (Magainin II; chain of Magainins; Frogs, amphibians, animals) | GIGKFLHSAKKFGKAFVGEIMNS |
| Bioactive peptide 1 (BAP1; Curvalicin-28a; Bacteriocin) | TPVVNPPFLQQT |
| Bioactive peptide 1 (BAP1; Curvalicin-28a; Bacteriocin) | TPVVNPPFLQQT |
| Thiocillin GE2270 (Antibiotic GE2270; Bacteriocin) | SCNCVCGFCCSCSP |
| Thiocillin GE2270 (Antibiotic GE2270; Bacteriocin) | SCNCVCGFCCSCSP |
| Gaegurin-RN1 (Frogs, amphibians, animals) | FIGPVLKIAAGILPTAICKIFKKC |
| Protein PR-L4 (Plant defensin) | SVFAFQDESTSTIAQARLFI |
| Maculatin-2.1 (Frogs, amphibians, animals) | GFVDFLKKVAGTIANVVT |
| Anacyclamide A10(Bacteriocin) | TSQTWGSPVP |
| V16M (truncated isoform of thanatin, residue 6-21) | VPIIYCNRRTGKCQRM |
| Temporin-ALh (Frogs, amphibians, animals) | FLPIVGKLLSGLSGLS |
| Cathelicidin-1 (Bactenecin-1, Bac1; Cyclic dodecapeptide; mammals, animals) | RLCRIVVIRVCR |
| Cathelicidin-1 (Bactenecin-1, Bac1; Cyclic dodecapeptide; mammals, animals) | RLCRIVVIRVCR |
| Pleurocidin-like peptide AP1 (NRC-11; fish, chordates, animals; Predicted) | GWKSVFRKAKKVGKTVGGLALDHYLG |
| [T5k]temporin-DRa (Frogs, amphibians, animals) | HFLGKLVNLAKKIL |
| Temporin-ALd (Frogs, amphibians, animals) | FLPIAGKLLSGLSGLL |
| Ranacyclin-B-RN6 (Frogs, amphibians, animals) | SALVGCWTKSYPPNPCFGRG |
| Citropin 1.1 M15 (Frogs, amphibians, animals) | GLFAVIKKVASVIKKL |
| Caerin-1.3 (Frogs, amphibians, animals) | GLLSVLGSVAQHVLPHVVPVIAEHL |
| I14M (truncated isoform of thanatin, residue 8-21) | IIYCNRRTGKCQRM |
| Maximin-S4 (chain of Maximins-S type B/C; toads, amphibians, animals) | RSNKGFNFMVDMIQALSK |
| Chaxapeptin (a class 2 lasso peptide; class 1 microcin, bacteriocins) | GFGSKPLDSFGLNFF |
| LFB A1 (derivative of LFB, residue substitution with alanine at position 1) | AKCRRWQWRMKKLGA |
| VESP-VB1 (Insects, animals) | FMPIIGRLMSGSL |
| G1 (Bac2A variant through single amino acid substitution) | GLARIVVIRVAR |
| UyCT5 (Arthropods, animals) | IWSAIWSGIKGLL |
| Cecropin A (1-8)-melittin (1-10)hybrid peptide | KWKLFKKIGIGAVLKVLT |
| Xenopsin precursor fragment (XPF; Frogs, amphibians, animals) | GWASKIGQTLGKIAKVGLKELIQPK |
| Caerin-2.1 (Frogs, amphibians, animals) | GLVSSIGRALGGLLADVVKSKGQPA |
| G20R (truncated isoform of thanatin, residue 1-20) | GSKKPVPIIYCNRRTGKCQR |
| Pln149 (Plantaricin 149; Bacteriocin; Derivatives: Pln149a) | YSLQMGATAIKQVKKLFKKKGG |
| Peptide BmKn2 (Biologically active peptide 4; NDBP-5.1; Arthropods, animals) | FIGAIARLLSKIF |
| Bac014 (Scrambled Variants of Bac2A) | RAVAVIIRLRRV |
| PAF26 (Trp-rich; combinatorial library) | RKKWFW |
| KR-20 (Derived from LL-37) | KRIVQRIKDFLRNLVPRTES |
| G2 (Bac2A variant through single amino acid substitution) | RGARIVVIRVAR |
| CNBr-cleaved lactoferricin Subfragment 2 | KKLGAPSITCVRRAFA |
| cathelicidin-BF15 (Snakes, reptiles, animals) | KFFRKLKKSVKKRAK |
| Bombinin-like peptide 2 (BLP-2; toads, amphibians, animals) | GIGSAILSAGKSALKGLAKGLAEHFAN |
| Odorranain-W1 (OdW1; Frogs, amphibians, animals) | GLFGKSSVWGRKYYVDLAGCAKA |
| NRC-3 (fish, chordates, animals) | GRRKRKWLRRIGKGVKIIGGAALDHL |
| Palustrin-2ISb-des-C7-des-N9 | GKKLFVNVLDKIRCKVAGGC |
| Actagardine (Gardimycin; Bacteriocin) | ASGWVCTLTIECGTVICAC |
| Cecropin A (1-7)-melittin (6-13)hybrid peptide | KWKLFKKLKVLTTGL |
| Temporin-ALd (Frogs, amphibians, animals) | FLPIAGKLLSGLSGLL |
| Ranacyclin-B-LK1 (Frogs, amphibians, animals) | SALVGCWTKSWPPKPCFGRG |
| R2 (Bac2A variant through single amino acid substitution) | RRARIVVIRVAR |
| Alyteserin-1a (toads, amphibians, animals) | GLKDIFKAGLGSLVKGIAAHVAN |
| CPF-SP1 (Frogs, amphibians, animals) | GFLGPLLKLGLKGVAKVLPHLIPSRQQ |
| Deserticolin-1 (Frogs, amphibians, animals) | GLADFLNKAVGKVVDFVKS |
| Leucocin H alpha(Bacteriocin) | WXIGVTGAALGTGKGVKNVI |
| Acipensin 6 (Ac6) | ILELAGNAARDNKKTRIIPRHLQL |
| Dermaseptin-1 (DShypo01, DPh-1; Frogs, amphibians, animals) | GLWSTIKNVGKEAAIAAGKAALGAL |
| UyCT3 (Arthropods, animals) | ILSAIWSGIKSLF |
| Odorranain-J-OA2 (Frogs, amphibians, animals) | GLFTLIKGAYKNDAPTVACN |
| Soybean toxin 17 kDa chain (SBTX 17 kDa chain; Plant defensin) | PNPKVFFDMTIGGQSAGRIVMEEYA |
| Temporin-ALi (Frogs, amphibians, animals) | FFPIVGKLLSGLL |
| Ocellatin-4 (Frogs, amphibians, animals) | GLLDFVTGVGKDIFAQLIKQI |
| UyCT2 (Arthropods, animals) | FWGKLWEGVKNAI |
| M-zodatoxin-Lt3a (M-ZDTX-Lt3a; Latarcin-3a, Ltc-3a; spiders, Arthropods, animals) | SWKSMAKKLKEYMEKLKQRA |
| Ranacyclin-B-RL1 (Frogs, amphibians, animals) | AALRGCWTKSIPPKPCPGKR |
| Protein PR-L2 (Plant defensin) | SVFAFENEQSSTIAPARLYK |
| Nigroain-B1 (Frogs, amphibians, animals) | CVISAGWNHKIRCKLTGNC |
| NRWC(Bacteriocin) | NRWCFAGDD |
| Aurein-2.1 (Frogs, amphibians, animals) | GLLDIVKKVVGAFGSL |
| L-amino-acid oxidase (LAAO, LAO, Oh-LAAO; Snakes, reptiles, animals) | HVINLEESFQEPEYENHLA |
| Bacteriocin ancovenin | CVQSCSFGPLTWSCDGNTK |
| Peptide BmKb1 (Non-disulfide-bridged peptide 4.2, NDBP-4.2; Arthropods, animals) | FLFSLIPSAISGLISAFK |
| Nigrocin-1-OR3 (Frogs, amphibians, animals) | GLISGLLGVGKMLVCGLSGLC |
| Nigrocin-1-OW2 (Frogs, amphibians, animals) | GILGNIVGMGKKIVCGLSGLC |
| D16W-N23 (single amino acid substitution) | GILDTLKQFAKGVGKWLVKGAAQ |
| Sclerosin(Bacteriocin) | TPALAVVTTVLPAAAVTTAKSV |
| Alyteserin-2Mb (toads, amphibians, animals) | ILGAIIPLVSGLLSHL |
| Novispirin G-10 (mutation of Ovispirin-1) | KNLRRIIRKGIHIIKKYG |
| Antimicrobial peptide PGQ (PGQ; Frogs, amphibians, animals) | GVLSNVIGYLKKLGTGALNAVLKQ |
| LL-23A9 (LL-23 variants) | LLGDFFRKAKEKIGKEFKRIVQR |
| Bacteriocin duramycin (Leucopeptin; Bacteriocin) | CKQSCSFGPFTFVCDGNTK |
| Nigroain-E1 (Frogs, amphibians, animals) | DCTRWIIGINGRICRD |
| Limnonectin-1Fa (Frogs, amphibians, animals) | SFPFFPPGICKRLKRC |
| Nigrocin-2ISa (Frogs, amphibians, animals) | GIFSTVFKAGKGIVCGLTGLC |
| Mutacin I (Bacteriocin) | FSSLSLCSLGCTGVKNPSFNSYCC |
| K18M (truncated isoform of thanatin, residue 4-21) | KPVPIIYCNRRTGKCQRM |
| Bacthuricin F4(Bacteriocin) | DWTXWSXL |
| UyCT1 (Arthropods, animals) | GFWGKLWEGVKNAI |
| Bac020 (Scrambled Variants of Bac2A) | RRAAVVLIVIRR |
| Nigrocin-1-OA3 (Frogs, amphibians, animals) | GIFLKVLGVGKKVLCGVSGLC |
| LFM A1 R9 W8 Y13 (LFM W8 derivative with residues substitution) | AKCLRWQWRMRKYGG |
| Dermaseptin-1 (DShypo01, DPh-1; Frogs, amphibians, animals) | GLWSTIKNVGKEAAIAAGKAALGAL |
| CGA47-70 (hrVS-1-derived peptide) | RILSILRHQNLLKELQDLALQGAK |
| Bac2A (a linear variant of bovine dodecapeptide) | RLARIVVIRVAR |
| Amolopin-2a (Frogs, amphibians, animals) | FLPIVGKLLSGLSGLL |
| Lantibiotic duramycin B (Bacteriocin) | CRQSCSFGPLTFVCDGNTK |
| Palustrin-1d (Frogs, amphibians, animals) | ALSILKGLEKLAKMGIALTNCKATKKC |
| Labyrinthopeptin A2 (Bacteriocin) | SDWSLWECCSTGSLFACC |
| Dybowskin-1CDYa (Frogs, amphibians, animals) | IIPLPLGYFAKKT |
| Pyrrhocoricin | VDKGSYLPRPTPPRPIYNRN |
| Grammistin Gs C (Group III grammistin; soapfish, chordates, animals) | NWRKILGKIAKVAAGLLGSMLAGYQV |
| Bacteriocin serracin-P 43 kDa subunit (Bacteriocin) | DYHHGVRVL |
| Cecropin A (1-8)-melittin (1-18)hybrid peptide | KWKLFKKIGIGAVLKVLTTGLPALIS |
| M-zodatoxin-Lt4b (M-ZDTX-Lt4b; Latarcin-4b, Ltc-4b; spiders, Arthropods, animals) | SLKDKVKSMGEKLKQYIQTWKAKF |
| Bacteriocin 97518 | ITSVSWCTPGCTSEGGGSGCSHCC |
| Dybowskin-4 (Frogs, amphibians, animals) | VWPLGLVICKALKIC |
| LMW peptide (Bacteriocin) | APVPFSCTRGCLTHLV |
| Plantaricin A(Bacteriocin) | AYSLQMGATAIKQVKKLFKKWGW |
| Alyteserin-1b (toads, amphibians, animals) | GLKEIFKAGLGSLVKGIAAHVAN |
| Fallaxidin 3.1 (Frogs, amphibians, animals) | GLLDLAKHVIGIASKL |
| Pediocin ACCEL(Bacteriocin) | KYYGNGVTXGKHSXXVDXG |
| Dermaseptin-H5 (Dermaseptin-like peptide 5, DMS5; Frogs, amphibians, animals) | GLWSTIKNVGKEAAIAAGKAVLGSL |
| Thuricin 4A-4 (bacteriocin) | WTTIVKVSKAVCKTGTCICTTSCSNCK |
| Metalnikowin-1 (Metalnikowin I; Insects, animals) | VDKPDYRPRPRPPNM |
| Ranacyclin-T (Frogs, amphibians, animals) | GALRGCWTKSYPPKPCK |
| M-zodatoxin-Lt5a (M-ZDTX-Lt5a; Latarcin-5, Ltc-5; spiders, Arthropods, animals) | GFFGKMKEYFKKFGASFKRRFANLKKRL |
| LFM A9 R1 W8 Y13 (LFM W8 derivative with residues substitution) | RKCLRWQWAMRKYGG |
| Sviceucin (bacteriocin) | CVWGGDCTDFLGCGTAWICV |
| Sviceucin (bacteriocin) | CVWGGDCTDFLGCGTAWICV |
| M-lycotoxin-Hc2a (M-LCTX-Hc2a; Lycotoxin-2; Lycotoxin II; spiders, Arthropods, animals) | KIKWFKTMKSIAKFIAKEQMKKHLGGE |
| Cathelicidin-6 (Antibacterial peptide BMAP-27, Myeloid antib | GRFKRFRKKFKKLFKKLSPVIPLLHLG |
| Cathelicidin-6 (Antibacterial peptide BMAP-27, Myeloid antib | GRFKRFRKKFKKLFKKLSPVIPLLHLG |
| Acidocin J1132 alpha peptide (Bacteriocin) | NPKVAHCASQIGRSTAWGAVSGA |
| Siamycin II(Bacteriocin) | CLGIGSCNDFAGCGYAIVCFW |
| Siamycin II(Bacteriocin) | CLGIGSCNDFAGCGYAIVCFW |
| Pleurocidin-like peptide AP2 (NRC-12; fish, chordates, animals; Predicted) | GWKKWFNRAKKVGKTVGGLAVDHYLG |
| Dybowskin-2 (Frogs, amphibians, animals) | FLIGMTQGLICLITRKC |
| Pleurocidin-like peptide GC3.8 (NRC-17; fish, chordates, animals; Predicted) | GWKKWLRKGAKHLGQAAIKGLAS |
| LFM R1,9 W8 Y13 (LFM W8 derivative with residues substitution) | RKCLRWQWRMRKYGG |
| Cecropin A (1-7)-melittin (2-9)hybrid peptide | KWKLFKKIGAVLKVL |
| Cecropin A (1-8)-melittin (1-12)hybrid peptide | KWKLFKKIGIGAVLKVLTTG |
| Ovispirin-1 (OV-1; N-terminal 18 amino acids of SMAP-29) | KNLRRIIRKIIHIIKKYG |
| Phylloxin (Frogs, amphibians, animals) | GWMSKIASGIGTFLSGMQQ |
| P18 (Cecropin A(1-8)-Magainin 2(1‚àí12) hybrid peptide analogue) | KWKLFKKIPKFLHLAKKF |
| Gramicidin S(Bacteriocin) | VKLFPVKLFP |
| R3 (Bac2A variant through single amino acid substitution) | RLRRIVVIRVAR |
| Ranacyclin B3 (Frogs, amphibians, animals) | AALKGCWTKSIPPKPCSGKR |
| Aurein-3.3 (Frogs, amphibians, animals) | GLFDIVKKIAGHIVSSI |
| Tricyclic peptide RP 71955 (Bacteriocin) | CLGIGSCNNFAGCGYAVVCFW |
| Tricyclic peptide RP 71955 (Bacteriocin) | CLGIGSCNNFAGCGYAVVCFW |
| Tricyclic peptide RP 71955 (Bacteriocin) | CLGIGSCNNFAGCGYAVVCFW |
| Tricyclic peptide RP 71955 (Bacteriocin) | CLGIGSCNNFAGCGYAVVCFW |
| VK10 (histone derived; reptiles; animals) | ARTKQTARKSTGGKAPRKQLAT |
| Odorranain-B1 (Frogs, amphibians, animals) | AALKGCWTKSIPPKPCFGKR |
| Bac8c (Bac2A variant) | RIWVIWRR |
| [L6]-IsCT (Mutant: W6L; IsCT analog) | ILGKILEGIKSLF |
| Nigrocin-1-OW5 (Frogs, amphibians, animals) | GILGNIVGMGKQVVCGLSGLC |
| Alyteserin-2Ma (toads, amphibians, animals) | FIGKLISAASGLLSHL |
| Gaegurin-6 (Gaegurin 6; GGN6; Frogs, amphibians, animals) | FLPLLAGLAANFLPTIICKISYKC |
| CP-P | KWKSFIKKLTSKFLHLAKKF |
| Grammistin Gs A (Group III grammistin; soapfish, chordates, animals) | WWRELLKKLAFTAAGHLGSVLAAKQSGW |
| Lantibiotic duramycin C (Bacteriocin) | CANSCSYGPLTWSCDGNTK |
| Japonicin-1CDYa (Frogs, amphibians, animals) | FFPLALLCKVFKKC |
| Citropin 1.1.3 (Frogs, amphibians, animals) | GLFDVIKKVASVIGLASP |
| Labyrinthopeptin A1(Bacteriocin) | SNASVWECCSTGSWVPFTCC |
| Uperin-3.5 (toads, amphibians, animals) | GVGDLIRKAVSVIKNIV |
| M-zodatoxin-Lt1a (M-ZDTX-Lt1a; Latarcin-1, Ltc-1, Ltc1; spiders, Arthropods, animals) | SMWSGMWRRKLKKLRNALKKKLKGE |
| Hominicin (Bacteriocin) | GTPATPFTPAITEITAAVIAG |
| Hlgut-defensin (H. longicornis midgut defensin; Ticks, Arthropods, animals; Predicted) | ACHAHCQSVGRRGGYCGNFRMTCYCY |
| Gallidermin (Bacteriocin; Preclinical) | IASKFLCTPGCAKTGSFNSYCC |
| Tigerinin-1 (Frogs, amphibians, animals) | FCTMIPIPRCY |
| Licheniocin 50.2(Bacteriocin) | WEEYNIIXQLGNKGQ |
| Actagardine (Gardimycin; Bacteriocin) | ASGWVCTLTIECGTVICAC |
| Actagardine (Gardimycin; Bacteriocin) | ASGWVCTLTIECGTVICAC |
| [K7]-IsCT (Mutant: E7K; IsCT analog) | ILGKIWKGIKSLF |
| ChaC7 (Chassatide C7; uncyclotides; Plant defensin) | IPCGESCVWIPCITAIAGCSCKNKVCYT |
| Hyicin 3682 (bacteriocin) | ITSFSLCTPGCAKTGSFNSYCC |
| Bac8a (Bac2A variant) | KIWVIRWR |
| Bac8b (Bac2A variant) | RIWVIRWR |
| Temporin-1Cc (Temporin 1Cc; Frogs, amphibians, animals) | FLPFLASLLTKVL |
| Bombinin (toads, amphibians, animals) | GIGALSAKGALKGLAKGLAEHFAN |
| Chensinin-1ZHa (Frogs, amphibians, animals) | LALKSGGWLRLFGLKDKKH |
| Temporin-1Ca (Temporin 1Ca; Frogs, amphibians, animals) | FLPFLAKILTGVL |
| H4-(86-100) (histone-derived) | VVYALKRNGRTLYGF |
| Hominicin (Bacteriocin) | ITPATPFTPAIITEITAAVIA |
| Nigroain-D3 (Frogs, amphibians, animals) | CVHWQTNTARTSCIGP |
| BI-32169(Bacteriocin) | GLPWGCPSDIPGWNTPWAC |
| BI-32169(Bacteriocin) | GLPWGCPSDIPGWNTPWAC |
| BI-32169(Bacteriocin) | GLPWGCPSDIPGWNTPWAC |
| BI-32169(Bacteriocin) | GLPWGCPSDIPGWNTPWAC |
| Vespid chemotactic peptide 5f (VCP 5f; Insects, animals) | FLPIPRPILLGLL |
| Antibacterial napin (Plants) | PAQPFRFPKHPQGPQTRPPI |
| Tigerinin-2 (Frogs, amphibians, animals) | RVCFAIPLPICH |
| Bacteriocin serracin-P 23 kDa subunit (Bacteriocin) | ALPKKLKYLNLFNDGFNYMGVV |
| Kassorin-S (PreproKassorin-S; Frogs, amphibians, animals) | FLGGILNTITGLL |
| LFC (fragment of mature caprine lactoferrin, residues 17 to 31) | SKCYQWQRRMRKLGA |
| Bass hepcidin (fish, chordates, animals) | GCRFCCNCCPNMSGCGVCCRF |
| Mutacin F-59.1 (Bacteriocin) | KYYGNGVTCGKHSXSVDWXKXT |
| LFB (fragment of bovine lalctoferricin, residues 17 to 31) | FKCRRWQWRMKKLGA |
| Nigroain-D3 (Frogs, amphibians, animals) | CVHWQTNTARTSCIGP |
| Myeloid antimicrobial peptide BMAP-27 (1-18) (mammals, animals) | GRFKRFRKKFKKLFKKLS |
| Myeloid antimicrobial peptide BMAP-27 (1-18) (mammals, animals) | GRFKRFRKKFKKLFKKLS |
| Chrysophsin-3 (fish, chordates, animals) | FIGLLISAGKAIHDLIRRRH |
| Bacteriocin 97518 | ITSVSWCTPGCTSEGGGSGCSHCC |
| Amphipathic peptide CT2 (StCT2; Non-disulfide-bridged peptide 5, NDBP-5; Arthropods, animals) | GFWGKLWEGVKSAI |
| Cerecidin A1(Bacteriocin) | TTPLCVGVIIGLTTSIKICK |
| W3 (Bac2A variant through single amino acid substitution) | RLWRIVVIRVAR |
| Odorranain-NR (Frogs, amphibians, animals) | GLLSGILGAGKHIVCGLTGCAKA |
| Odorranain-W2 (Frogs, amphibians, animals) | GLLRASSVWGRKYYVDLAGCAKA |
| Palustrin-2AJ1 (PL2AJ1; Frogs, amphibians, animals) | GFMDTAKNVAKNVAVTLIDKLRCKVTGGC |
| G12 (Bac2A variant through single amino acid substitution) | RLARIVVIRVAG |
| HNr (histone-derived) | VVYTLKRNGRTLYGF |
| PhTD-3 (PhTD3; primates, mammals, animals) | RCVCTRGFCRCVCTRGFC |
| Antimicrobial peptide 2 (AamAP2; Arthropods, animals) | FPFSLIPHAIGGLISAIK |
| Tigerinin-4 (Frogs, amphibians, animals) | RVCYAIPLPIC |
| Palustrin-2ISc (Frogs, amphibians, animals) | GFMDTAKNVAKNVAATLLDKLKCKITGGC |
| Pleurocidin-like peptide YT2 (NRC-7; fish, chordates, animals; Predicted) | RWGKWFKKATHVGKHVGKAALTAYL |
| Crabrolin (Insects, animals) | FLPLILRKIVTAL |
| HalA2 (one chain of haloduracin; Bacteriocin) | TTWPCATVGVSVALCPTTKCTSQC |
| dPSM | GIIKVIKSLIEQFTGK |
| Tricyclic peptide RP 71955 (Bacteriocin) | CLGIGSCNDFAGCGYAVVCFW |
| Tricyclic peptide RP 71955 (Bacteriocin) | CLGIGSCNDFAGCGYAVVCFW |
| Sub3 (Bac2A variant through three amino acids substitution) | RRWRIVVIRVRR |
| Caerin-1.11 (Frogs, amphibians, animals) | GLLGAMFKVASKVLPHVVPAITEHF |
| M-zodatoxin-Lt3b (M-ZDTX-Lt3b; Latarcin-3b, Ltc-3b; spiders, Arthropods, animals) | SWASMAKKLKEYMEKLKQRA |
| Pleurocidin-like peptide GcSc4C5 (NRC-14; fish, chordates, animals) | AGWGSIFKHIFKAGKFIHGAIQAHND |
| Odorranain-J-OA1 (Frogs, amphibians, animals) | GLFTLIKGAYKLDAPTVACN |
| W10 (Bac2A variant through single amino acid substitution) | RLARIVVIRWAR |
| Distinctin 2 (Frogs, amphibians, animals) | NLVSGLIEARKYLEQLHRKLKNCKV |
| Siamycin(Bacteriocin) | CLGVGSCNDFAGCGYAIVCFW |
| Siamycin(Bacteriocin) | CLGVGSCNDFAGCGYAIVCFW |
| Brevinin-1Lb (Frogs, amphibians, animals) | FLPMLAGLAASMVPKFVCLITKKC |
| Anantin(Bacteriocin) | GFIGWGNNIFGHYSGDF |
| Anantin(Bacteriocin) | GFIGWGNNIFGHYSGDF |
| Nigrocin-OR3 (Frogs, amphibians, animals) | GILSGLLGVGKMLVCGLSGLC |
| Pleurocidin-like peptide WF3 (NRC-5; fish, chordates, animals) | FLGALIKGAIHGGRFIHGMIQNHH |
| Warnericin RK (Bacteriocin) | MQFITDLIKKAVDFFKGLFGNK |
| Panitide L2 (plants) | QLPICGETCVLGGCYTPNCRCQYPICVR |
| NRC-2 (fish, chordates, animals) | WLRRIGKGVKIIGGAALDHL |
| Cypemycin (Bacteriocin) | ATPATPTVAQFVIQGSTICLVC |
| Cypemycin (Bacteriocin) | ATPATPTVAQFVIQGSTICLVC |
| Kassinatuerin-1 (Frogs, amphibians, animals) | GFMKYIGPLIPHAVKAISDLI |
| Peptide leucine arginine (pLR; Frogs, amphibians, animals) | LVRGCWTKSYPPKPCFVR |
| Ranatuerin-1C (Ranatuerin 1C; Frogs, amphibians, animals) | SMLSVLKNLGKVGLGLVACKINKQC |
| Lantibiotic michiganin-A (Bacteriocin) | SSSGWLCTLTIECGTIICACR |
| Bacteriocin serracin-P 23 kDa subunit (Bacteriocin) | ALPKKLKYLNLFNDGFNYMGVV |
| Bacteriocin cinnamycin (Lanthiopeptin Ro 09-0198) | CRQSCSFGPFTFVCDGNTK |
| Aurein-3.2 (Frogs, amphibians, animals) | GLFDIVKKIAGHIASSI |
| LFC W8 (tryptophan-modified caprine lactoferricin derivative) | SKCYQWQWRMRKLGA |
| Bombinin-H1/H3 (Frogs, amphibians, animals) | IIGPVLGMVGSALGGLLKKI |
| Bacteriocin duramycin (Leucopeptin; Bacteriocin) | CKQSCSFGPFTFVCDGNTK |
|  |  |
| Bacteriocin lacticin 3147 A2 (LtnA2; Bacteriocin; Preclinical) | TTPATPAISILSAYISTNTCPTTKCTRAC |
|  |  |
| Brevinin-1RTa (Frogs, amphibians, animals) | FLPLLAGVVANFLPQIICKIARKC |
| Lantibiotic duramycin C (Bacteriocin) | CANSCSYGPLTWSCDGNTK |
| Brevinin-1Ba (Frogs, amphibians, animals) | FLPFIAGMAAKFLPKIFCAISKKC |
| Piceain 1 (Plants) | KSLRPRCWIKIKFRCKSLKF |
| Citropin-2.1 (Frogs, amphibians, animals) | GLIGSIGKALGGLLVDVLKPKL |
| Chrysophsin-1 (fish, chordates, animals) | FFGWLIKGAIHAGKAIHGLIHRRRH |
| Bactofencin A (Bacteriocin) | KRKKHRCRVYNNGMPTGMYRWC |
| Aurein-2.5 (Frogs, amphibians, animals) | GLFDIVKKVVGAFGSL |
| Nigroain-E1 (Frogs, amphibians, animals) | DCTRWIIGINGRICRD |
| Aurein-5.2 (Frogs, amphibians, animals) | GLMSSIGKALGGLIVDVLKPKTPAS |
| Citropin 1.1 M14 (Frogs, amphibians, animals) | GLFDVIAKVASVIKKL |
| Microbisporicin A1 (Bacteriocin) | VTSWSLCTPGCTSPGGGSNCSFCC |
| Hejiangin-F1 (frog, amphibians, animals) | IPWKLPATFRPVERPFSKPFCRKD |
| Bacteriocin mutacin B-Ny266 (Preclinical) | FKSWSFCTPGCAKTGSFNSYCC |
| BsaA2(Bacteriocin) | ITSHSLCTPGCAKTGSFNSFCC |
| Nigrocin-OR1 (Frogs, amphibians, animals) | GILSGILGVGKKLVCGLSGLC |
| Microcin J25 (MccJ25; Bacteriocin) | GGAGHVPEYFVGIGTPISFYG |
| Microcin J25 (MccJ25; Bacteriocin) | GGAGHVPEYFVGIGTPISFYG |
| Preprotemporin-1SKa (Frogs, amphibians, animals) | FLPVILPVIGKLLNGILGK |
| Antimicrobial peptide ctriporin (Riporin; Arthropods, animals) | FLWGLIPGAISAVTSLIKK |
| Mutacin-1140 (Mutacin III; Bacteriocin) | FKSWSLCTPGCARTGSFNSYCC |
| Pleurocidin-like peptide WF4 (NRC-6; fish, chordates, animals) | GWGSIFKHGRHAAKHIGHAAVNHYL |
| Cerecidin A7(Bacteriocin) | TTPLCVGVIIGITASIKICK |
| Tilapia piscidin 4 (TP4; Oreoch-2; MSP-4; fish, animals) | FIHHIIGGLFSAGKAIHRLIRRRRR |
| Iseganan(IB-367) | RGGLCYCRGRFCVCVGR |
| Sub2 (Bac2A variant through two amino acids substitution) | RLRRIVVIRVRR |
| Lariatins B (lasso peptide; Bacteriocin) | GSQLVYREWVGHSNVIKGPP |
| LFP W8 (tryptophan-modified porcine lactoferricin derivative) | SKCRQWQWKIRRTNP |
| NRC-10 (fish, chordates, animals) | FFRLLFHGVHHVGKIKPRA |
| Metchnikowin-2 (Insects, animals) | RRQGPIFDTRPSPFNPNQPRPGPIY |
| Schmackerin-C1 (Frogs, amphibians, animals) | AAPRGGKGFFCKLFKDC |
| Tigerinin-3 (Frogs, amphibians, animals) | RVCYAIPLPICY |
| Grammistin Gs 2 (Grammistin Gs G; Group I grammistin; soapfish, chordates, animals) | LFGFLIPLLPHIIGAIPQVIGAIR |
| Odorranain-P1a (OdP1a; Brevinin-1HS1; Brevinin-1-OA2; Frogs, amphibians, animals) | VIPFVASVAAEMMQHVYCAASKKC |
| hLF1-11 | GRRRRSVQWCA |
| Temporin-ALf (Frogs, amphibians, animals) | FFPIVGKLLSGLSGLL |
| Gaegurin-5 (Gaegurin 5; GGN5; Brevinin-1EMa; Frogs, amphibians, animals) | FLGALFKVASKVLPSVFCAITKKC |
| Kassinatuerin-2Ma (Frogs, amphibians, animals) | FLGAIAAALPHVINAVTNAL |
| Dermaseptin-like PBN2 (DRP-PBN2; Plasticin-B1a; Frogs, amphibians, animals) | GLVTSLIKGAGKLLGGLFGSVTGGQS |
| M-zodatoxin-Lt2a (M-ZDTX-Lt2a; Latarcin-2a, Ltc-2a, Ltc2a; spiders, Arthropods, animals) | GLFGKLIKKFGRKAISYAVKKARGKH |
| PlnA-17 (Bacteriocin) | GATAIKQVKKLFKKWGW |
| Piceain 2 (Plants) | RPRCWIKIKFRCKSLKF |
| F3 (single amino acid substitution of Bac034, which is a scrambled Variant of Bac2A) | VRFRIRVAVIRA |
| Gramicidin C (GC; Bacteriocin) | VGALAVVVWLYLWLW |
| LFB-RI (derivative of bovine lactoferrin with residues substitution) | RRIIIRWRRI |
| LL-23V9 (LL-23 variants) | LLGDFFRKVKEKIGKEFKRIVQR |
| Salivaricin A (SalA; Bacteriocin; Preclinical) | KRGSGWIATITDDCPNSVFVCC |
| Ranalexin (Frogs, amphibians, animals) | FLGGLIKIVPAMICAVTKKC |
| Fallaxidin 4.1 (Frogs, amphibians, animals) | GLLSFLPKVIGVIGHLIHPPS |
| Bac-GM100 (Bacteriocin) | DWTFANWSCLVCDDCSVNLTV |
| BacCH91(Bacteriocin) | ITSFIGCTPGCGKTGSFNSFCC |
| Cytotoxic linear peptide IsCT2 (Non-disulfide-bridged peptide 5.3, NDBP-5.3; Arthropods, animals) | IFGAIWNGIKSLF |
| Buforin-2 (Buforin II; Fragment of Histone H2A; toads, amphibians, animals) | TRSSRAGLQFPVGRVHRLLRK |
| Iseganan(IB-367) | RGGLCYCRGRFCVCVGR |
| Andersonin-X1 (Frogs, amphibians, animals) | GLFSKFAGKGIVNFLIEGVE |
| Hylin-a1 (Hy-a1; Frogs, amphibians, animals) | IFGAILPLALGALKNLIK |
| Nigrocin-1-OW4 (Frogs, amphibians, animals) | GILSGVLGMGKKIVCGLRGLC |
| Dybowskin-6 (Frogs, amphibians, animals) | FLPLLLAGLPLKLCFLFKKC |
| Caerin-1.1 (Frogs, amphibians, animals) | GLLSVLGSVAKHVLPHVVPVIAEHL |
| Phylloseptin-3 (PS-3; Frogs, amphibians, animals) | FLSLIPHAINAVSALANHG |
| Salivaricin 9 (Sal9; Bacteriocin) | GNGVVLTLTHECNLATWTKKLKCC |
| Lactococcin K (Bacteriocin) | MVPTTFTLTTNNFLSDYQQLFI |
| Palustrin-1c (Frogs, amphibians, animals) | ALSILRGLEKLAKMGIALTNCKATKKC |
| Kasstasin (Frogs, amphibians, animals) | FIKELLPHLSGIIDSVANAIK |
| Thuricin-S (Bacteriocin) | DWTXWSXLVXAACSVELL |
| Lantibiotic epidermin (Bacteriocin) | IASKFICTPGCAKTGSFNSYCC |
| Bacteriocin ancovenin | CVQSCSFGPLTWSCDGNTK |
| Fallaxidin 3.2 (Frogs, amphibians, animals) | GLLDFAKHVIGIASKLG |
| Cecropin A (1-7)-melittin (4-11)hybrid peptide (CAM) | KWKLFKKAVLKVLTT |
| Limnonectin-1Fb (Frogs, amphibians, animals) | SFHVFPPWMCKSLKKC |
| Dybowskin-1 (Frogs, amphibians, animals) | FLIGMTHGLICLISRKC |
| PL-101 | FFHLHFHY |
| [T5k]temporin-DRa (Frogs, amphibians, animals) | HFLGKLVNLAKKIL |
| Pleurocidin-like peptide Hb18 (NRC-20; fish, chordates, animals; Predicted) | GFLGILFHGVHHGRKKALHMNSERRS |
| Gramicidin B (GB; Bacteriocin) | VGALAVVVWLFLWLW |
| Phylloseptin-1 (PS-1; Frogs, amphibians, animals) | FLSLIPHAINAVSAIAKHN |
| LFB A2 (derivative of LFB, residue substitution with alanine at position 2) | FACRRWQWRMKKLGA |
| Endochitinase 2 (Plant defensin) | SAIWFWMTPQSPK |
| Andersonin-W1 (Frogs, amphibians, animals) | ATNIPFKVHFRCKAAFC |
| Senegalin | FLPFLIPALTSLISSL |
| Antimicrobial peptide 1 (AamAP1; Arthropods, animals) | FLFSLIPHAIGGLISAFK |
| cLf 20-29 (fragment of caprine lalctoferricin, residues 20-29) | YQWQRRMRKL |
| Nigroain-C2 (Frogs, amphibians, animals) | FKTWKRPPFQTSCWGIIKE |
| Formicin (bacteriocin) | CEWYNISCQLGNKGQWCTLTKECQRSCK |
| Antimicrobial peptide 2 (XT-2; Frogs, amphibians, animals) | GVWSTVLGGLKKFAKGGLEAIVNPK |
| Palustrin-2ISa (Frogs, amphibians, animals) | GFMDTAKNVAKNVAVTLLDKLKCKITGGC |
| Nigrocin-OR2 (Frogs, amphibians, animals) | GILSGILGVGKMLVCGLSGLC |
| Acidocin J1132 beta peptide (Bacteriocin) | GNPKVAHCASQIGRSTAWGAVSGA |
| Acidocin J1132 beta peptide (Bacteriocin) | GNPKVAHCASQIGRSTAWGAVSGA |
| LL-37(13-37)(C-terminal fragment of LL-37; Human, mammals, animals) | IGKEFKRIVQRIKDFLRNLVPRTES |
| LFB-KW (derivative of bovine lactoferrin with residues substitution) | KKWWWKWKKW |
| Andersonin-Y1 (Frogs, amphibians, animals) | FLPKLFAKITKKNMAHIR |
| Apidaecin-1B (Apidaecin IB; Insects, animals) | GNNRPVYIPQPRPPHPRL |
| Hepcidin (fish, chordates, animals) | RCRFCCRCCPRMRGCGICCRF |
| RES-701-1(Bacteriocin) | GNWHGTAPDWFFNYYW |
| RES-701-1(Bacteriocin) | GNWHGTAPDWFFNYYW |
| Pleurain-A1 (Pleurain A1; Frogs, amphibians, animals) | SIITMTKEAKLPQLWKQIACRLYNTC |
| Ranacyclin-B-RN1 (Frogs, amphibians, animals) | SALVGCWTKSYPPKPCFGR |
| LFB-Rwa (derivative of bovine lactoferrin with residues substitution) | RRWWRRWRRW |
| Bac8d (Bac2A variant) | RRWVIWRR |
| Nigroain-B1 (Frogs, amphibians, animals) | CVISAGWNHKIRCKLTGNC |
| M-zodatoxin-Lt4a (M-ZDTX-Lt4a; Latarcin-4a, Ltc-4a; spiders, Arthropods, animals) | GLKDKFKSMGEKLKQYIQTWKAKF |
| Citropin-2.1.3 (Frogs, amphibians, animals) | GLIGSIGKALGGLLVDVLKPKLQAAS |
| Chrysophsin-2 (fish, chordates, animals) | FFGWLIRGAIHAGKAIHGLIHRRRH |
| Andersonin-C1 (Frogs, amphibians, animals) | TSRCIFYRRKKCS |
| Bacteriocin cinnamycin (Lanthiopeptin Ro 09-0198) | CRQSCSFGPFTFVCDGNTK |
| Ranalexin-1Cb (Ranatuerin 1Cb; Frogs, amphibians, animals) | FLGGLMKAFPAIICAVTKKC |
| CylLS (a structural subunit of cytolysin; Bacteriocin) | TTPACFTIGLGVGALFSAKFC |
| Pln149 (Plantaricin 149; Bacteriocin; Derivatives: Pln149a) | YSLQMGATAIKQVKKLFKKKGG |
| Ranacyclin-B-LK2 (Frogs, amphibians, animals) | SALVGCWTKSWPPKPCFGR |
| Dermadistinctin-Q2 (DD Q2; Frogs, amphibians, animals) | GLWSKIKEAAKTAGLMAMGFVNDMV |
| LSEI_2386 (m2386; Bacteriocin; Predicted) | DSIRDVSPTFNKIRRWFDGLFK |
| Andersonin-W2 (Frogs, amphibians, animals) | AVNIPFKVHFRCKAAFC |
| Antimicrobial peptide 1 (XT-1; Frogs, amphibians, animals) | GFLGPLLKLAAKGVAKVIPHLIPSRQQ |
| Bac034 (Scrambled Variants of Bac2A) | VRLRIRVAVIRA |
| CNBr-cleaved lactoferricin Subfragment 1 | FKCRRWQWR |
| Aurein-3.1 (Frogs, amphibians, animals) | GLFDIVKKIAGHIAGSI |
| LFH W8 (tryptophan-modified human lactoferricin derivative) | TKCFQWQWNMRKVRG |
| Lariatins A (lasso peptide; Bacteriocin) | GSQLVYREWVGHSNVIKP |
| LFB-6RW (derivative of bovine lactoferrin with residues substitution) | RRWWWR |
| TsAP-2 (T. serrulatus antimicrobial peptide 2; scorpions, arachnids, invertebrates, animals) | FLGMIPGLIGGLISAFK |
| LFB A3 (derivative of LFB, residue substitution with alanine at position 3) | FKARRWQWRMKKLGA |
| Dybowskin-2CDYa (Chensinin-1; Frogs, amphibians, animals) | SAVGRHGRRFGLRKHRKH |
| Phylloseptin 12 (PS-12; Frogs, amphibians, animals) | FLSLLPSIVSGAVSLAKKLG |
| Gomesin (Gm; Spiders, arachnids, Chelicerata, arthropods, invertebrates, animals) | QCRRLCYKQRCVTYCRGR |
| Pleurocidin-like peptide GC3.2 (NRC-18; fish, chordates, animals; Predicted) | GWKKWFTKGERLSQRHFA |
| Capistruin (Bacteriocin) | GTPGFQTPDARVISRFGFN |
| NRC-16 (fish, chordates, animals) | GWKKWLRKGAKHLGQAAIK |
| Bombinin-H5 (Frogs, amphibians, animals) | IIGPVLGLVGSALGGLLKKI |
| Sub5 (Bac2A variant through five amino acids substitution) | RRWKIVVIRWRR |
| PGLa (chain of PYLa/PGLa A; Frogs, amphibians, animals) | GMASKAGAIAGKIAKVALKAL |
| ChaC11 (Chassatide C11; uncyclotides; Plant defensin) | IPCGESCVWIPCISGMFGCSCKDKVCYS |
| Metchnikowin (Pro-rich; Insects, animals) | HRHQGPIFDTRPSPFNPNQPRPGPIY |
| Maculatin-1.4 (frog, amphibia, animals) | GLLGLLGSVVSHVLPAITQHL |
| Temporin-ALg (Frogs, amphibians, animals) | FFPIVGKLLFGLFGLL |
| Phylloseptin-1 (PSN-1; Frogs, amphibians, animals) | FLSLIPHIVSGVASIAKHF |
| GLFcin II (Lactoferrin fragment) | SKCYQWQRRMRKLGAPSITCIRRTS |
| Tachyplesin I (Tac; TP1; Horseshoe Crab, arachnids, Chelicerata, arthropods, invertebrates, animals) | KWCFRVCYRGICYRRCR |
| Andersonin-D1 (Frogs, amphibians, animals) | FIFPKKNIINSLFGR |
| Prepromelittin-related peptide (Frogs, amphibians, animals) | AIGSILGALAKGLPTLISWIKNR |
| Ranacyclin B5 (Frogs, amphibians, animals) | AALRGCWTKSIPPKPCSGKR |
| Antimicrobial peptide 4 (XT-4; Frogs, amphibians, animals) | GVFLDALKKFAKGGMNAVLNPK |
| Pleurain-A2 (Pleurain A2; Frogs, amphibians, animals) | SIITMTKEAKLPQSWKQIACRLYNTC |
| PAC113 | AKRHHGYKRKFH |
| Pleurocidin (NRC-4; fish, chordates, animals) | GWGSFFKKAAHVGKHVGKAALTHYL |
| Japonicin-1 (Frogs, amphibians, animals) | FFPIGVFCKIFKTC |
| MSI-78(Pexiganan) | GIGKFLKKAKKFGKAFVKILKK |
| Geobacillin II (nisin analog; Bacteriocin) | STIVCVSLRICNWSLRFCPSFKVRCPM |
| M-lycotoxin-Hc1a (M-LCTX-Hc1a; Lycotoxin I; spiders, Arthropods, animals) | IWLTALKFLGKHAAKHLAKQQLSKL |
| Maculatin-1.1 (Frogs, amphibians, animals) | GLFGVLAKVAAHVVPAIAEHF |
| LFB-RF (derivative of bovine lactoferrin with residues substitution) | RRFFFRFRRF |
| Nigroain-C2 (Frogs, amphibians, animals) | FKTWKRPPFQTSCWGIIKE |
| Gramicidin A (GA; Nonribosomally synthesized bacteriocin) | VGALAVVVWLWLWLW |
| Brevinin-1SY (Frogs, amphibians, animals) | FLPVVAGLAAKVLPSIICAVTKKC |
| Ranacyclin-B-AL1 (Frogs, amphibians, animals) | AAFRGCWTKNYSPKPCL |
| CP10A (Indolicidin peptide derivative; mammals, animals) | ILAWKWAWWAWRR |
| CP10A (Indolicidin peptide derivative; mammals, animals) | ILAWKWAWWAWRR |
| Venom antimicrobial peptide-9 (Meucin-18; NDBP-5; Arthropods, animals) | FFGHLFKLATKIIPSLFQ |
| Cathelicidin-5 (Antibacterial peptide BMAP-28; Myeloid antib | GGLRSLGRKILRAWKKYGPIIVPIIRIG |
| Cathelicidin-5 (Antibacterial peptide BMAP-28; Myeloid antib | GGLRSLGRKILRAWKKYGPIIVPIIRIG |
| Phylloxin (Frogs, amphibians, animals) | GWMSKIASGIGTFLSGMQQ |
| Hejiangin-A1 (Frogs, amphibians, animals) | RFIYMKGFGKPRFGKR |
| Lantibiotic duramycin B (Bacteriocin) | CRQSCSFGPLTFVCDGNTK |
| moroPC-NH2 (moronecidin-like peptide; fish, animals) | FFGHLFRGIINVGKHIHGLLSG |
| Pleurocidin-like peptide Hb26 (NRC-19; fish, chordates, animals; Predicted) | FLGLLFHGVHHVGKWIHGLIHGHH |
| Gallidermin (Bacteriocin; Preclinical) | IASKFLCTPGCAKTGSFNSYCC |
| Streptin 1 (Bacteriocin) | VGSRYLCTPGSCWKLVCFTTTVK |
| Lantibiotic streptin (Bacteriocin) | GSRYLCTPGSCWKLVCFTTTVK |
| bLf 20-29 (fragment of bovine lactoferricin, residues 20-29) | RRWQWRMKKL |
| Hominicin (Bacteriocin) | ITPATPFTPAIITEITAAVIA |
| Prepromelittin-related peptide (Frogs, amphibians, animals) | VIGSILGALASGLPTLISWIKNR |
| Variacin (Bacteriocin) | GSGVIPTISHECHMNSFQFVFTCCS |
| Frenatin-3 (Frogs, amphibians, animals) | GLMSVLGHAVGNVLGGLFKS |
| Pleurocidin-like peptide WFY (fish, chordates, animals) | FFRLLFHGVHHGGGYLNAA |
| Ranalexin-Vb (Frogs, amphibians, animals) | FLGGLFKLVPSVICAVTKKC |
| Temporin-ALe (Frogs, amphibians, animals) | FFPIVGKLLFGLSGLL |
| NAI-112(Bacteriocin) | VSTLSVSSPCPGWPSSFTWSNC |
| Japonicin-2 (Frogs, amphibians, animals) | FGLPMLSILPKALCILLKRKC |
| Siamycin I (Bacteriocin) | CLGVGSCNDFAGCGYAVVCFW |
| Bacteriocin mutacin B-Ny266 (Preclinical) | FKSWSFCTPGCAKTGSFNSYCC |
| Conolysin-Mt1 | FHPSLWVLIPQYIQLIRKILKSG |
| LFB-RW (derivative of bovine lactoferrin with residues substitution) | RRWWWRWRRW |
| Grammistin Gs 1 (Grammistin Gs F; Group I grammistin; soapfish, chordates, animals) | LFGFLIKLIPSLFGALSNIGRNRNQ |
| Arenicin-1 (Ar-1; marine polychaeta, animals) | RWCVYAYVRVRGVLVRYRRCW |
| DRP-PBN1 (Frogs, amphibians, animals) | FLSLIPHIVSGVAALAKHLG |
| Sub6 (Bac2A variant through six amino acids substitution) | RWWKIWVIRWWR |
| Phylloseptin-7 (PS-7; Frogs, amphibians, animals) | FLSLIPHAINAVSAIAKHF |
| Palustrin-1b (Frogs, amphibians, animals) | ALFSILRGLKKLGNMGQAFVNCKIYKKC |
| PlnA-22 (Bacteriocin) | YSLQMGATAIKQVKKLFKKWGW |
| Pleurocidin-like peptide GcSc4B7 (NRC-15; fish, chordates, animals; Predicted) | GFWGKLFKLGLHGIGLLHLHL |
| Amolopin-1b (Frogs, amphibians, animals) | FLPLAVSLAANFLPKLFCKITKKC |
| TsAP-1 (T. serrulatus antimicrobial peptide 1; scorpions, arachnids, invertebrates, animals) | FLSLIPSLVGGSISAFK |
| Lividin-1 (Brevinin-1-OR2; Frogs, amphibians, animals) | ILPFVAGVAAEMMQHVYCAASKKC |
| Phylloseptin-2 (PS-2; Frogs, amphibians, animals) | FLSLIPHAINAVSTLVHHF |
| Gramicidin B (GB; Bacteriocin) | VGALAVVVWLFLWLW |
| Ranalexin-1G (Frogs, amphibians, animals) | FLGGLMKIIPAAFCAVTKKC |
| Mutacin-1140 (Mutacin III; Bacteriocin) | FKSWSLCTPGCARTGSFNSYCC |
| Ruminococcin A (RumA; Bacteriocin) | GNGVLKTISHECNMNTWQFLFTCC |
| Caerin-1.9 (Frogs, amphibians, animals) | GLFGVLGSIAKHVLPHVVPVIAEKL |
| Butyrivibriocin OR79 (Bacteriocin; Predicted) | GNGVIKTISHECHMNTWQFIFTCCS |
| Soybean toxin 27 kDa chain (SBTX 27 kDa chain; Plant defensin) | ADPTFGFTPLGLSEKANLQIMKAYD |
| Temporin-L (Temporin-1Tl; temporin-Tl; TL; Frogs, amphibians, animals) | FVQWFSKFLGRIL |
| Brevinin-1RTb (Frogs, amphibians, animals) | FLGSLLGLVGKVVPTLFCKISKKC |
| Gramicidin C (GC; Bacteriocin) | VGALAVVVWLYLWLW |
| Brevinin-1Pb (Frogs, amphibians, animals) | FLPIIAGIAAKVFPKIFCAISKKC |
| Streptococcin A-FF22 (Antibacterial peptide SA-FF22; Bacteriocin) | GKNGVFKTISHECHLNTWAFLATCCS |
| Gramicidin A (GA; Nonribosomally synthesized bacteriocin) | VGALAVVVWLWLWLW |
| Phylloseptin-7 (PS-7; Frogs, amphibians, animals) | FLSLIPHAINAVSAIAKHFG |
| Cytotoxic linear peptide IsCT2 (Non-disulfide-bridged peptide 5.3, NDBP-5.3; Arthropods, animals) | IFGAIWNGIKSLF |
| Salivaricin B (SboB; Bacteriocin; Preclinical) | GGGVIQTISHECRMNSWQFLFTCCS |

| **DBASSP** | |
| --- | --- |
| **Peptide Name** | **Peptide Sequence** |
| Ib-AMP4 | QWGRRCCGWGPGRRYCRRWC |
| Epinecidin-1 | FIFHIIKGLFHAGKMIHGLVTRRRH |
| Tilapia piscidin 3, TP3 | FIHHIIGGLFSVGKHIHSLIHGH |
| Arenicin-3 | GFCWYVCVYRNGVRVCYRRCN |
| Clavaspirin | FLRFIGSVIHGIGHLVHHIGVAL |
| Dermaseptin-SP4 | SLWSSIKDMAAAAGRAALNAVNGILNP |
| Brevinin-1Pb | FLPIIAGIAAKVFPKIFCAISKKC |
| Moomysin | SYHWGDYHDWHHGWYGWWDD |
| Marcin-22, MAC-22 | FFGHLFKLATKIIPSFFRRKNQ |
| Fallaxidin 4.1 | GLLSFLPKVIGVIGHLIHPPS |
| Magainin-AM1, Magainin-F1, Magainin-R2, Magainin-BM1 | GIKEFAHSLGKFGKAFVGGILNQ |
| Vaby A | GLPVCGETCAGGTCNTPGCSCSWPICTRN |
| Clavanin D | AFKLLGRIIHHVGNFVYGFSHVF |
| Fowlicidin, Myeloid antimicrobial peptide 27 | RFGRFLRKIRRFRPKVTITIQGSARFG |
| Pleurain-A1 | SIITMTKEAKLPQLWKQIACRLYNTC |
| Piscidin-1, Pis1 | FIHHIIGWISHGVRAIHRAIHG |
| Cyclotide chassatide C4 | GASCGETCFTGICFTAGCSCNPWPTCTRN |
| Hymenochirin-4B | IKIPAFVKDTLKKVAKGVISAVAGALTQ |
| Brevinin-1SPb | FLPIIAGMAAKVICAITKKC |
| Megin 2 | FFVLKFLLKWAGKVGLEHLACKFKNWC |
| Brevinin-Bf | FLPFIAGMAANFLPKIFCAISKKC |
| Rock Bream Piscidin 7, RP7 | FFGRLKAVFRGARQGWKEHRY |
| MS Piscidin-2 | FLKHIKSFWRGAKAIFRGARQGWREHR |
| Caerin 1.11 | GLFSVLGSVAKHVVPRVVPVIAEHL |
| Clavanin C | VFHLLGKIIHHVGNFVYGFSHVF |
| Clavanin C | VFHLLGKIIHHVGNFVYGFSHVF |
| Kasseptin 1Md | IIGAIAAALPHVINAIKNTF |
| Brevinin-1CG4 | FLSTLLNVASKVVPTLFCKITKKC |
| Phylloseptin-B1, PBN1 | FLSLIPHIVSGVAALAKHLG |
| Arenicin-1 | RWCVYAYVRVRGVLVRYRRCW |
| Grammistin GsF, Grammistin Gs 1 | LFGFLIKLIPSLFGALSNIGRNRNQ |
| Pteroicidin-alpha, alpha-Pte | FIHHIIGGLFHVGKSIHDLIR |
| Pteroicidin-alpha, alpha-Pte-AMD | FIHHIIGGLFHVGKSIHDLIR |
| Conolysin-Mt | FHPSLWVLIPQYIQLIRKILKSG |
| Brevinin-1-OR9 | ILPFVAGVAAMEMEHVYCAASKKC |
| Clavanin A | VFQFLGKIIHHVGNFVHGFSHVF |
| ChBac7.5Nalpha, Bac7.5 protein (102-123) | RRLRPRRPRLPRPRPRPRPRPR |
| Ponericin-W5 | FWGALIKGAAKLIPSVVGLFKKKQ |
| Brevinin CTcu4 | FLPFIAGMAAKFLPKIFCAISKK |
| Gaduscidin-1 | FIHHIIGWISHGVRAIHRAIH |
| Gaegurin-LK1 | FIGPVLKMATSILPTAICKGFKKC |
| Hainanenin-1 | FALGAVTKLLPSLLCMITRKC |
| Antifungal protein, Psc-AFP | EWEPVQNGGSSYYMVPRIWA |
| M-poneratoxin-Dq3a, Dq-2562 | FWGTLAKWALKAIPAAMGMKQNK |
| Cathelicidin ModoCath5 | WYQLIRTFGNLIHQKYRKLLEAYRKLRD |
| Lactoferricin B | FKCRRWQWRMKKLGAPSITCVRRAFA |
| XPF-SE2 | GLASTIGSLLGKFAKGGAQAFLQPK |
| Piscidin-3 | FIHHIFRGIVHAGRSIGRFLTG |
| Sd-Piscidin | FIHHIIKGIFHIGKMIHSAINRRRH |
| Moronecidin-like peptide PC | FFGHLFRGIINVGKHIHGLLSG |
| Fowlicidin 3, Cathelicidin-3 | KRFWPLVPVAINTVAAGINLYKAIRRK |
| M-poneritoxin-Nc1a | FWGAAAKMLGKALPGLISMFQKN |
| Piscidin Trematocine | FFGHLLRGIVSVGKHIHGLITG |
| Cathelicidin-5, Myeloid antibacterial peptide 28, BMAP-28 | GGLRSLGRKILRAWKKYGPIIVPIIRIG |
| BMAP28 | GGLRSLGRKILRAWKKYGPIIVPIIRIG |
| Cathelicidin-3, CATH-3 | RVKRFWPLVPVAINTVAAGINLYKAIRRK |
| Prepromelittin-related peptide, Peptide VR-23 | VIGSILGALASGLPTLISWIKNR |
| Pandinin-2, Pin2, Non-disulfide-bridged peptide 4.1, NDBP-4.1 | FWGALAKGALKLIPSLFSSFSKKD |
| Cathelicidin PAM2 | RPWAGNGSVHRYTVLSPRLKTQ |
| Cathelicidin-3.4, ChBac3.4 | RFRLPFRRPPIRIHPPPFYPPFRRFL |
| Cathelicidin-3.4, ChBac3.4 | RFRLPFRRPPIRIHPPPFYPPFRRFL |
| Japonicin-2LF | FIVPSIFLLKKAFCIALKKC |
| Python Cathelicidin CATHPb4 | TRSRWRRFIRGAGRFARRYGWRIALGLVG |
| Ranatuerin-2TOb | GLLNVIKDTAQNLFAAALEKLKCKVTKCN |
| Lycotoxin-1, M-lycotoxin-Hc1a | IWLTALKFLGKHAAKHLAKQQLSKL |
| Lycotoxin I, M-lycotoxin-Hc1a | IWLTALKFLGKHAAKHLAKQQLSKL |
| Odorranain-J-OA2 | GLFTLIKGAYKNDAPTVACN |
| Hepcidin 1, Hepcidin 6 | CRFCCRCCPRMRGCGLCCRF |
| Pleurocidin | GWGSFFKKAAHVGKHVGKAALTHYL |
| Pleurocidin -AMD, NRC-04 | GWGSFFKKAAHVGKHVGKAALTHYL |
| Pleurocidin | GWGSFFKKAAHVGKHVGKAALTHYL |
| SmHep1P, Sm-Hepcidin-1 | QSHISLCRWCCNCCKANKGCGFCCKF |
| Fusogenic peptide FP23, Envelope glycoprotein gp160 (517-539) | AVGIGALFLGFLGAAGSTMGARS |
| Piscidin-2, Pis2 | FLHHIVGLIHHGLSLFGDRAD |
| Brevinin-1CG2 | FLPIVAGLAANFLPKIVCKITKKC |
| Truncated apolipoprotein C-I, APOC1(64-88), Apo5 | FSTKTRNWFSEHFKKVKEKLKDTFA |
| Antimicrobial peptide odorranain B6, Ranacyclin B5 | AALRGCWTKSIPPKPCSGKR |
| Piscidin 1 WB | FFHHIFRGIVHVGKTIHKLVTG |
| Brevinin-1SN1 | FLPAVLKVAAHILPTAICAISRRC |
| Brevinin-1ISa | FLPGVLRLVTKVGPAVVCAITRNC |
| Halocyntin | FWGHIWNAVKRVGANALHGAVTGALS |
| Kasseptin 1Ma | FLGAIAAALPHVINAVTNAL |
| Meucin-25 | VKLIQIRIWIQYVTVLQMFSMKTKQ |
| Metchnikowin, Mtk-1 | HRHQGPIFDTRPSPFNPNQPRPGPIY |
| Cathelicidin CATH3, Cc-CATH3 | RVRRFWPLVPVAINTVAAGINLYKAIRRK |
| Brevinin-1-OR10 | FLPAVLLVATHVLPTVFCAITRKC |
| PGLa | GMASKAGAIAGKIAKVALKAL |
| Maculatin 1.4 | GLLGLLGSVVSHVLPAITQHL |
| Brevinin-1RL1 | FFPLIAGLAARFLPKIFCSITKRC |
| Chionodracine | FFGHLYRGITSVVKHVHGLLSG |
| Microcin J25 | GGAGHVPEYFVGIGTPISFYG |
| Python Cathelicidin CATHPb2 | KRNGFRKFMRRLKKFFAGGGSSIAHIKLH |
| PGLa-AM1, PGLa-R1, PGLa-BM1 | GMASKAGSVLGKVAKVALKAAL |
| Tp-HAMP2 | RKRCRFCCNCCPGKQGCVFCCGF |
| Ascaphin-1 | GFRDVLKGAAKAFVKTVAGHIAN |
| Ranatuerin-2SSa | GLISTIWNTASNVAGTLTDSVKCKFKKC |
| Brevinin-1BYb | FLPILASLAAKLGPKLFCLVTKKC |
| Ubonodin | GGDGSIAEYFNRPMHIHDWQIMDSGYYG |
| NCR247, MtN27 family | RNGCIVDPRCPYQQCRRPLYCRRR |
| Brevinin family peptide Yodha | SMLLLFFLGTISLSLCQDDQERC |
| Caerin-1.5 | GLLSVLGSVVKHVIPHVVPVIAEHL |
| Oreochromicin 1 | FIHHIIGGLFSVGKHIHGLIHGH |
| Caerin-1.8 | GLFKVLGSVAKHLLPHVVPVIAEKL |
| Brevinin-1VLa | FLGAIAGVAAKFLPKVFCFITKKC |
| Chensinin-1CEb | LALERRSGWLRLFGLKPRRKH |
| Astacidin PcAst-1b/c | SNVYRPPPYRPVYRPLRRPGYRP |
| Cathelicidin-2, CATH-2 | RFGRFLRKIRRFRPKVTITIQGSARF |
| Clavanin-B | VFQFLGRIIHHVGNFVHGFSHVF |
| Caerin-1.2 | GLLGVLGSVAKHVLPHVVPVIAEHL |
| Piscidin 1, Moronecidin | FFHHIFRGIVHVGKTIHRLVTG |
| Cathelicidin Cl, dCATH | KRFWQLVPLAIKIYRAWKRR |
| Brevinin1 HYba2 Cyclic AMD | FFPGIIKVAGAILPTAICAITKRC |
| Cathelicidin-1, CATH-1, Fowlicidin-1 | RVKRVWPLVIRTVIAGYNLYRAIKKK |
| Cathelicidin-1, CATH-1, Fowlicidin-1 | RVKRVWPLVIRTVIAGYNLYRAIKKK |
| Cu Hepcidin, CuHepc | QSHLSLCRWCCNCCHNKSCGFCCKF |
| Metchnikowin 2, Mtk-2 | HRRQGPIFDTRPSPFNPNQPRPGPIY |
| Chrysophsin-2 | FFGWLIRGAIHAGKAIHGLIHRRRH |
| Moronecidin-like peptide NC | FFWHHIGHALDAAKRVHGMLSG |
| Bacteriocin plantaricin A, PlnA | YSLQMGATAIKQVKKLFKKWGW |
| ToAP2 | FFGTLFKLGSKLIPGVMKLFSKKKER |
| Brevinin-1AA1 | FFPSIAGLAAKFLPKIFCSITKRC |
| M-zodatoxin-Lt4a, Latarcin 4a, Ltc-4a | GLKDKFKSMGEKLKQYIQTWKAKF |
| Brevinin-Bc | FLPFIAGVAAKFLPKIFCAISKKC |
| Lycosin-II | VWLSALKFIGKHLAKHQLSKL |
| Kassinatuerin-1 | GFMKYIGPLIPHAVKAISDLI |
| Odorranin-HP, Odorranain-W-RA1 | GLLRASSVWGRKYYVDLAGCAKA |
| Dermaseptin S4 | ALWMTLLKKVLKAAAKAALNAVLVGANA |
| Carnobacteriocin Y, CbnY | SAILAITLGIFATGYGMGVQKAINDRRKK |
| MS Piscidin-3 | FIFHVIKGLFHAGKMIHGLVTRRRH |
| Brevinin-1PLb | FLPLIAGLAANFLPKIFCAITKKC |
| Brevinin-1-OR6 | IIPFVAGVAAEMMEHVYCAASKKC |
| Brevinin-1-AJ1 | FLSTLLKVAFKVVPTLFCPITKKC |
| Brevinin-1PRc | FFPMLAGVAARVVPKVICLITKKC |
| Brevinin-1PLc | FLPVIAGVAAKFLPKIFCAITKKC |
| Maculatin-1.1 | GLFGVLAKVAAHVVPAIAEHF |
| Hymenochirin-5Ph | ITIPPIVKNTLKKFIKGAVSALMS |
| Glycine-rich protein, GRPSp | IPAMEPAARVKRSPGYGGCSPRWACGGYG |
| Arenicin-2 | RWCVYAYVRIRGVLVRYRRCW |
| LSEI_2163, m2163 | KRKCPKTPFDNTPGAWFAHLILGC |
| Ranalexin-1Ca | FLGGLMKAFPALICAVTKKC |
| Brevinin-1AUb | FLPILAGLAANILPKVFCSITKKC |
| Brevinin-ALb, Amolopin-1b | FLPLAVSLAANFLPKLFCKITKKC |
| Plantaricin S alpha, Pls-alpha | RNKLAYNMGHYAGKATIFGLAAWALLA |
| Horse cathelicidin 2, Myeloid cathelicidin 2, eCATH-2 | KRRHWFPLSFQEFLEQLRRFRDQLPFP |
| SA-hepcidin 1 | QSHLSMCRYCCNCCRNNKGCGFCCKF |
| Odorranain-F-OW1 | GFMNTAKNVAKNVAVTLLDNLKCKITGGC |
| Bombinin-like peptide 1, BLP-1 | GIGASILSAGKSALKGLAKGLAEHFAN |
| Cathelicidin As-CATH7 | KRVNWRKVGRNTALGASYVLSFLG |
| Circulin-F | AIPCGESCVWIPCISAAIGCSCKNKVCYR |
| Hepcidin TH2-2 | GIKCCFCCGCCNSGVCELCCRF |
| Cathelicidin Gg-CATH5 | TRRKWWKKVLNGAIKIAPYILD |
| Toxin LyeTx 1 | IWLTALKFLGKNLGKHLAKQQLAKL |
| Brevinin-1VLd | FLPLIAGVAANFLPKIFCLISKKC |
| Of Moronecidin, Rbmoro | FFHHIFNGLVGVGKTIHRLI |
| Antimicrobial peptide 1, XT-1 | GFLGPLLKLAAKGVAKVIPHLIPSRQQ |
| Brevinin-1HN1 | FLPLIASLAANFVPKIFCKITKKC |
| Dermaseptin DMS-DA6 | GVWGIAKIAGKVLGNILPHVFSSNQS |
| Phylloseptin-4, PS-4 | FLSLIPHAINAVSTLVHHSG |
| Cyclotide Cter M, Cyclotide cliotide T3 | GLPTCGETCTLGTCYVPDCSCSWPICMKN |
| Hejiangin-F1 | IPWKLPATFRPVERPFSKPFCRKD |
| Brevinin-Be | FLPAIVGAAAKFLPKIFCVISKKC |
| Moronecidin | IFHHIFKGIVHVGKTIHRLVTG |
| As Cathelicidin 5, As-CATH5 | TRRKFWKKVLNGALKIAPFLLG |
| Cloacaenodin | GHSVDRIPEYFGPPGLPGPVLFYS |
| Brevinin-1-OR7 | QLPFVAGVACEMCQCVYCAASKKC |
| Caerin-1.4 | GLLSSLSSVAKHVLPHVVPVIAEHL |
| Urumin | IPLRGAFINGRWDSQCHRFSNGAIACA |
| Cyclotide mela-7 | GLPTCGETCFKGKCYTPGCSCSYPICKKN |
| Brevinin 2-Ia | GIKSALLGIAKNVGMSLLQKAQCKLSGSC |
| Maximin 9 | GIGRKFLGGVKTTFRCGVKDFASKHLY |
| Magainin-B1 | GKFLHSAGKFGKAFLGEVMIG |
| Neutrophil defensin 2, HNP-2 | CYCRIPACIAGERRYGTCIYQGRLWAFCC |
| Cyclotide chassatide C8, Uncyclotide ChaC8 | AIPCGESCVWIPCISTVIGCSCSNKVCYR |
| Kasseptin 1Mc | FVGAIAAALPHVISAIKNAL |
| Caerin-1.1 | GLLSVLGSVAKHVLPHVVPVIAEHL |
| Cathelicidin-5, myeloid antimicrobial peptide BMAP-28 | GGLRSLGRKILRAWKKYGPIIVPIIRI |
| Nigrocin-1-OW4 | GILSGVLGMGKKIVCGLRGLC |
| Palustrin-2ISd | GFMSTASNVLTNVAGTVMDKLKCKFTGAC |
| Buforin-2, Histone H2A | TRSSRAGLQFPVGRVHRLLRK |
| Cycloviolacin O15 | GLVPCGETCFTGKCYTPGCSCSYPICKKN |
| Brevinin-1ULf | FFGSTIGALANFLPSLISKIRN |
| As Cathelicidin 6, As-CATH6 | TRWLWLLRGGLKAAGWGIRAHLNRNQ |
| Clavanin E | LFKLLGKIIHHVGNFVHGFSHVF |
| Brevinin-1 | FLPVLAGIAAKVVPALFCKITKKC |
| ChMAP-28, MAP28 Protein (102-128) | GRFKRFRKKLKRLWHKVGPFVGPILHY |
| Brevinin-1BYa | FLPILASLAAKFGPKLFCLVTKKC |
| Gaegurin-5 | FLGALFKVASKVLPSVKCAITKKC |
| Varv E, Varv peptide E | GLPICGETCVGGTCNTPGCSCSWPVCTRN |
| Odorranain-M2 antimicrobial peptide, Odorranain-M1 | ATAWDFGPHGLLPIRPIRIRPLCGKDKS |
| Pseudhymenochirin-2Pa | GIFPIFAKLLGKVIKVASSLISKGRTE |
| Lividin-1 protein, Brevinin-1-OR1 | LPFVAGVAAEMMQHVYCAASKKC |
| Caerin-1.20 | GLFGILGSVAKHVLPHVIPVVAEHL |
| Brevinin-1LTa | FFGTALKIAANVLPTAICKILKKC |
| So-Piscidin | IWGLIAHGVAHVGSLIHGLVNG |
| Alarin | APAHRSSTFPKWVTKTERGRQPLRS |
| Latarcin-2a, Ltc-2a | GLFGKLIKKFGRKAISYAVKKARGKH |
| Pseudhymenochirin-1Pb | IKIPSFFRNILKKVGKEAVSLIAGALKQS |
| Marmelittin, Peptide BmKb1 | FLFSLIPSAISGLISAFKGRRKRDLN |
| Brevinin-Bb | FLPAIAGMAAKFLPKIFCAISKKC |
| Cereucin XB, CexB | MKYLGTLIKGAAGGAGAYVGEKIYNWYKN |
| Dermaseptin-S9 | GLRSKIWLWVLLMIWQESNKFKKM |
| RV-23 | RIGVLLARLPKLFSLFKLMGKKV |
| Maximin 5 | SIGAKILGGVKTFFKGALKELASTYLQ |
| Trichoplaxin | FFGRLKSVWSAVKHGWKAAKSR |
| Gaegurin-5 | FLGALFKVASKVLPSVFCAITKKC |
| Raniseptin-1 | AWLDKLKSLGKVVGKVALGVAQNYLNPQQ |
| Brevinin -1 Gra, Brevinin-1E-OG6 | FLPLLAGLAANFLPKIFCKITKKC |
| Odorranain-P1a, Brevinin-1-OA2 | VIPFVASVAAEMMQHVYCAASKKC |
| Grammistin Gs G, Grammistin Pp 4a | LFGFLIPLLPHIIGAIPQVIGAIR |
| Meucin-22, MUC-22 | FFGHLFKLATKIIPSLFQRKKE |
| Psyle C | KLCGETCFKFKCYTPGCSCSYPFCK |
| Brevinin-1E | FLPLLAGLAANFLPKIFCKITRKC |
| Brevinin-1RE2 | VIPFVASVAAEMMQHVYCAASKRC |
| Cathelicidin CATH3, Pc-CATH1 | RIKRFWPVVIRTVVAGYNLYRAIKKK |
| Tilapia piscidin 4, TP4 | FIHHIIGGLFSAGKAIHRLIRRRRR |
| Piscidin-4, Oreochromicin 2 | FIHHIIGGLFSAGKAIHRLIRRRRR |
| Pleurocidin-like peptide WF4, NRC-06 | GWGSIFKHGRHAAKHIGHAAVNHYL |
| Brevinin-1Ed | VIPFVASVAAEMMHHVYCAASKRC |
| Cliotide T21 | DLQCAETCVHSPCIGPCYCKHGLICYRN |
| Moronecidin-like peptide | FFRNLWKGAKAAFRAGHAAWRA |
| Putative antimicrobial peptide A Pacific variant, Ci-PAP-A22 | ALRSAVRTVARVGRAVLPHVAI |
| Varv peptide F | GVPICGETCTLGTCYTAGCSCSWPVCTRN |
| Dermaseptin-H10, DRS-H10 | GLWSTIKNVAAAAGKAALGAL |
| Bactofencin A | KRKKHRCRVYNNGMPTGMYRWC |
| Brevinin-1DYa, Amurin-2c | FLSLALAALPKFLCLVFKKC |
| Chrysophsin-1 | FFGWLIKGAIHAGKAIHGLIHRRRH |
| Amurin-2a | FLPLLLAGLPSFLCLVFKKC |
| Dybowskin-6 | FLPLLLAGLPLKLCFLFKKC |
| Brevinin-1Sc | FFPIVAGVAGQVLKKIYCTISKKC |
| Piceain 1 | KSLRPRCWIKIKFRCKSLKF |
| FPA-Bombinin-BO | GIGGALLSAGKAALKGLAKGLAEHFAN |
| Brevinin-Ba | FLPFIAGMAAKFLPKIFCAISKKC |
| Maximin H55 | ILGPVISTIGNALGGLLKNL |
| Brevinin-1Ma | FLPILAGLAANLVPKLICSITKKC |
| CPF-LM1 | GFGSFLGSLFKTGLKIIPKLLPSIQQ |
| Horse cathelicidin 1, Myeloid cathelicidin 1, eCATH-1 | KRFGRLAKSFLRMRILLPRRKILLAS |
| Brevinin-1RTa | FLPLLAGVVANFLPQIICKIARKC |
| Lividin-1 protein, Brevinin-1-OR2 | ILPFVAGVAAEMMQHVYCAASKKC |
| Palustrin-1b | ALFSILRGLKKLGNMGQAFVNCKIYKKC |
| Hepcidin 2, AS-hepc2 | SPAGCRFCCGCCPNMRGCGVCCRF |
| Sm-Moronecidin | FFRHIVGAISRIFGQKQRDMAD |
| Maximin 3 | GIGGKILSGLKTALKGAAKELASTYLH |
| Ascaphin-5 | GIKDWIKGAAKKLIKTVASHIANQ |
| Piscidin-like peptide, Pc-Piscidin | IWGLIAHGVGHVGRLIHGLIRG |
| Somuncurin-4.2 | YYQVSEERRRDLASLARLYALAR |
| Cyclotide mela-2 | GKPTCGETCFKGKCYTPGCTCSYPLCKKD |
| Cathelicidin ModoCath4 | SKTKRRSLLKRLGDGIRGFWNGFRGRK |
| Pictuseptin-1 | GFLDTLKNIGKTVGRIALNVLT |
| Magainin-SE1 | GLKEVLHSTKKFAKGFITGLTGQ |
| Antixoidant peptide, Odorranain-J-OA1 | GLFTLIKGAYKLDAPTVACN |
| Brevinin-1LTc, Brevinin-1LT1 | FMGSALRIAAKVLPAALCQIFKKC |
| U2-theraphotoxin-Pc1a, Psalmopeotoxin II, PcFK2 | RCLPAGKTCVRGPMRVPCCGSCSQNKCT |
| Dermaseptin-B3, Dermaseptin DRS-DI4-like peptide, Dermaseptin-6, DStar 06 | ALWKNMLKGIGKLAGQAALGAVKTLVGA |
| Brevinin-1BLc, Brevinin-1BLa | FLPIIAGIAAKFLPKIFCTISKKC |
| Pelteobagrin | GKLNLFLSRLEILKLFVGAL |
| Kassinatuerin-2 | FIQYLAPLIPHAVKAISDLI |
| Warnericin RK | MQFITDLIKKAVDFFKGLFGNK |
| Pleurocidin-like peptide WF3, NRC-05 | FLGALIKGAIHGGRFIHGMIQNHH |
| Lycosin-I | RKGWFKAMKSIAKFIAKEKLKEHL |
| Maximin 28 | GIGTKFLGGVKTALKGALKELASTYVN |
| Hymenochirin-2B | LKIPGFVKDTLKKVAKGIFSAVAGAMTPS |
| XPF-C1 | GWASKIGQALGKVAKVGLQQFIQPK |
| Androctonin | RSVCRQIKICRRRGGCYYKCTNRPY |
| Cathelin-related peptide SC5, Antibacterial peptide SMAP-29, Myeloid antibacterial peptide MAP-29 | RGLRRLGRKIAHGVKKYGPTVLRIIRIAG |
| Pro-adrenomedullin, PAMP | ARLDVASEFRKKWNKWALSR |
| Brevinin-2-related peptide B-GR23, 2GHe | GVTFNALKGVAKTVAAQLLKTAR |
| Brevinin-1CSa | FLPILAGLAAKIVPKLFCLATKKC |
| Brevinin-1AA2 | FLPAVLRVAAKVGPAVFCAITQKC |
| Brevinin-1Lb | FLPMLAGLAASMVPKFVCLITKKC |
| Odorranain-O-RA peptide, Odorranain-k1 | AVPLIYNRPGIYVTKRPKGK |
| Tu-AMP2 beta chain | TPRPVCAATCDCKIITGTKCPPGYEK |
| Melittin-S | GIGAVLKVLSTGLPALISWIKRKRQQ |
| Peptide 73-L, 73L | RLWDIVRRWVGWLGPLGVRG |
| Antimicrobial peptide 1 | VGECVRGRCPSGMCCSQFGYCGKGPKYCG |
| Maximin H4 | ILGPVISKIGGVLGGLLKNL |
| Tricyclic peptide MS-271 | CLGVGSCNDFAGCGYAIVCFW |
| Siamycin I | CLGVGSCNDFAGCGYAIVCFW |
| Bombinin-BO1 | GIGSAILSAGKSIIKGLAKGLAEHF |
| Caerin-1.6 | GLFSVLGAVAKHVLPHVVPVIAEKL |
| Cyclotide chassatide C7, Uncyclotide ChaC7 | IPCGESCVWIPCITAIAGCSCKNKVCYT |
| Brevinin-1HSb, Brevinin-1JDb | FLPAVLRVAAQVVPTVFCAISKKC |
| Brevinin-1-OR4 | INPFVAGVAAEMMQHVYCAASKKC |
| Kalata Polypeptides K1 | PICTRNGLPVCGETCFGGTCNTPGCTCTW |
| Dermaseptin-01 / DS 01 | GLWSTIKQKGKEAAIAAAKAAGQAALGAL |
| Smp24 | IWSFLIKAATKLLPSLFGGGKKDS |
| M-zodatoxin-Lt3b, Latarcin-3b, Ltc-3b | SWASMAKKLKEYMEKLKQRA |
| Ranatuerin-2P | LMDTVKNVAKNLAGHMLDKLKCKITGC |
| Streptomonomicin, STM | SLGSSPYNDILGYPALIVIYP |
| Aborycin | CLGIGSCNDFAGCGYAVVCFW |
| Dicentracin-like peptide | FLRSLLRGAKAIYRGARAGWRG |
| Tilapia piscidin 2, TP2 | GECIWDAIFHGAKHFLHRLVNP |
| Antimicrobial protein plp, Peptide HKPLP | FLGLIFHGLVHAGKLIHGLIHRNRG |
| Kalata Polypeptides K2 | PVCTRNGLPVCGETCVGGTCNTPGCTCSW |
| Vitri C | GLPICGETCVGGTCNTPGCFCTWPVCTRN |
| Ponericin-L2 | LLKELWTKIKGAGKAVLGKIKGLL |
| Ranatuerin-1Ga | SMISVLKNLGKVGLGFVACKVNKQC |
| Maximin H52 | ILGLVISTIGNVLGGLLKNL |
| Brevinin-1V | FLPLIASVAANLVPKIFCKITKKC |
| Sm-Hepcidin 1 | QSHLSLCRWCCNCCNRYKGCGFCCKF |
| Brevinin-1Sa | FLPAIVGAAGQFLPKIFCAISKKC |
| Brevinin-1Ya | FLPVIAGVAANFLPKLFCAISKKC |
| Hainanenin-5 | FALGAVTKRLPSLFCLITRKC |
| Cyanophlyctin | FLNALKNFAKTAGKRLKSLLN |
| Brevinin-1DYb, Amurin-2 | FLSLALAALPKLFCLIFKKC |
| Brevinin-1OKa | FFGSMIGALAKGLPSLISLIKK |
| Salusin-beta | AIFIFIRWLLKLGHHGRAPP |
| M-poneritoxin-Na1b | FLGALLKIGAKLLPSVVGLFKKKQQ |
| Vitri D | GLPVCGETCFTGSCYTPGCSCNWPVCNRN |
| Caerin-5.1, Rothein 4.1 | AEILFGDVRPPWMPPPIFPEMP |
| Ranacyclin-AJ | AAFRGCWTKSYSPKPCLGKR |
| Phyloseptin-SP1 | FLSLIPHVISAIPHVVNALSNL |
| Caerin-1.18 | GLFSVLGSVAKHLLPHVVPVIAEKL |
| Cathelicidin As-CATH8 | KRVNWAKVGRTALKLLPYIFG |
| Brevinin CTcu5 | LIAGLAANFLPQILCKIARKC |
| SMAP28, SMAP-29, Cathelin-related peptide SC5 | RGLRRLGRKIAHGVKKYGPTVLRIIRIA |
| Piscidin-2 beta, Pis2 beta | FLHHIVGLIHHGKLDMYRSNN |
| Shepherin I, Antimicrobial peptide shep-GRP | GYGGHGGHGGHGGHGGHGGHGHGGGGHG |
| Pleurain-J1 antimicrobial peptide | FIPGLRRLFATVVPTVVCAINKLPPG |
| MS Piscidin-1 | FLGTLLHGAVHVSKILHGIMGGDH |
| Brevinin-1SPd | FFPIIAGMAAKVICAITKKC |
| Kalata Polypeptides K3 | PICKRNGLPVCGETCTLGTCYTQGCTCSW |
| Brevinin-1-OR5 | ILPFVAGVAAEMMKHVYCAASKKC |
| Chrysophsin-3 | FIGLLISAGKAIHDLIRRRH |
| Dermaseptin-PS2 | ALWKTLLKNVGKAAGKAVLNAVTDMVNQ |
| Hepcidin | GCRFCCNCCPNMSGCGVCCRF |
| Kassiniatuerin-3 | FIQHLIPLIPHAIQGIKDIF |
| Plantaricin S beta, Pls-beta | KKKKQSWYAAAGDAIVSFGEGFLNAW |
| Brevinin-1LF1 | FLPMLAGLAANFLPKIICKITKKC |
| Ishikawain-5 | SPYRCGSPDSRGSENTRCLIKK |
| Ranatuerin-2SKa | GLLDAIKDTAQNLFANVLDKIKCKFTKC |
| Con10 | FWSFLVKAASKILPSLIGGGDDNKSSS |
| Caerin-1.7 | GLFKVLGSVAKHLLPHVAPVIAEKL |
| Brevinin CTcu2 | FLPLLAGLAANFLPKIFCKITRK |
| Dermaseptin-4, Dermaseptin-S4 | ALWMTLLKKVLKAAAKALNAVLVGANA |
| Lividin-D1 | KNNFCQVLYVWLLRLGKQCFVKFSKDVET |
| XPF-SE4 | GVWTTILGGLKKFAKGGLEALTNPK |
| Cliotide T15 | GLPICGETCFKTKCYTKGCSCSYPVCKRN |
| EA-CATH1, EA Cathelicidin 1 | KRRGSVTTRYQFLMIHLLRPKKLFA |
| Bacteriocin plantaricin A | KSSAYSLQMGATAIKQVKKLFKKWGW |
| Cyclotide 6, Viba 17 | GLPVCGETCVGGTCNTPGCGCSWPVCTRN |
| D94, Bacteriocin plantaricin J, Bacteriocin PlnJ | GAWKNFWSSLRKGFYDGEAGRAIRR |
| Brevinin-1OKc | FFGSIIGALAKGLPSLISLIKK |
| Magainin-1 | GIGKFLHSAGKFGKAFVGEIMKS |
| Brevinin-1BLa | FLPAIVGAAAKFLPKIFCAISKKC |
| BMAP-27 | GRFKRFRKKFKKLFKKLSPVIPLLHL |
| Phenol-soluble modulin alpha 4 peptide, PSMalpha4 | MAIVGTIIKIIKAIIDIFAK |
| Cruzioseptin-1, CZS-1 | GFLDIVKGVGKVALGAVSKLF |
| Ocellatin-PT1 | GVFDIIKDAGKQLVAHAMGKIAEKV |
| Magainin-B2, Magainin-MW1 | GIGKFLHSAGKFGKAFLGEVMKS |
| Orexin B, ORXB | RSGPPGLQGRLQRLLQASGNHAAGILTM |
| Pleurain-D1 antimicrobial peptide | FLSGILKLAFKIPSVLCAVLKNC |
| Odorranain-MISa | ATAWNLGPHGLRPIRPIRIRPLCGKDKS |
| Ranalexin-1G | FLGGLMKIIPAAFCAVTKKC |
| Lariatin B | GSQLVYREWVGHSNVIKGPP |
| CPF-B1 | GLGSLLGKAFKIGLKTVGKMMGGAPREQ |
| BHV12L-Bombinin (26-50), BHL-bombinin | GIGGALLSFGKSALKGLAKGLAEHF |
| Cathelicidin-BF-AMD | KFFRKLKKSVKKRAKEFFKKPRVIGVSIPF |
| Brevinin-1TP3 | FLPGLIKVAVGVGSTILCKITKKC |
| Picturin-3 | GVFKDALKQFGAALLDQAANALKPK |
| mBjAMP1 | NLCASLRARHTIPQCRKFGRR |
| PMAP-23, Myeloid antibacterial peptide 23 | RIIDLLWRVRRPQKPKFVTVWVR |
| PMAP-23, Myeloid antibacterial peptide 23 | RIIDLLWRVRRPQKPKFVTVWVR |
| Hepcidin-1, BpHep-1 | QSHLSMCRWCCNCCRGNKGCGPCCKF |
| Odorranain-J1 | GLFTLIKCAYQLIAPTVACN |
| Ponericin-W6 | FIGTALGIASAIPAIVKLFK |
| Brevinin-1PRa | FLPVLTGLTPSIVPKLVCLLTKKC |
| Brevinin-1PRb | FLPVLAGLTPSIVPKLVCLLTKKC |
| Cyclotide chassatide C10 | GEYCGESCYLIPCFTPGCYCVSRQCVNKN |
| Hepcidin, Bthepc, CtHep | QSHLSLCRWCCNCCHNKGCGFCCKF |
| Hepcidin-2, BpHep-2 | GIKCKFCCGCCTPGVCGLCCRF |
| Palustrin-2GN2 | GFMDTAKNVFKNVAVTLLDKLKCKIAGGC |
| Ranatuerin-2CHa | GLMDTVKNAAKNLAGQLLDRLKCKITGC |
| SA-hepcidin 2 | NPAGCRFCCGCCPNMIGCGVCCRF |
| Melittin | GIGAVLKVLTTGLPALISWIKRKRQQ |
| Melittin | GIGAVLKVLTTGLPALISWIKRKRQQ |
| M-zodatoxin-Lt1a, Latarcin-1, Ltc-1 | SMWSGMWRRKLKKLRNALKKKLKGE |
| Hymenochirin-5Pa | ITIPPIVKDTLKKFFKGGIAGVMGKSQ |
| Alvinellacin | RGCYTRCWKVGRNGRVCMRVCT |
| Kalata-B1 | GLPVCGETCVGGTCNTPGCTCSWPVCTRN |
| Vitri B | GYPICGESCVGGICNIPGCSCSWPVCTTN |
| Nigrocin-1-OR3 | GILSGLLGVGKMLVCGLSGLC |
| Grammistin Gs A | WWRELLKKLAFTAAGHLGSVLAAKQSGW |
| Histatin 5 | DSHAKRHHGYKRKFHEKHHSHRGY |
| Histatin 5 | DSHAKRHHGYKRKFHEKHHSHRGY |
| Brevinin-1SPa | FFPIIAGMAAKLIPSLFCKITKKC |
| Gaegurin-6 | FLPLLAGLAANFLPTIICKISYKC |
| Nigrocin-1-OW5 | GILGNIVGMGKQVVCGLSGLC |
| Maximin 78 | GIGGALLSVGKLALKGLANVLADKFAN |
| Brevinin-1TRa | FLPVIAGIAANVLPKLFCKLTKRC |
| Maximin 77 | GIGGALLSAGKSALKGLAKGLAEHL |
| Pituitary adenylate cyclase-activating polypeptide 38, PACA27 | HSDGIFTDSYSRYRKQMAVKKYLAAVL |
| Odorranain-B1 | AALKGCWTKSIPPKPCFGKR |
| Antimicrobial peptide odorranain B4, Ranacyclin B3 | AALKGCWTKSIPPKPCSGKR |
| Brevinin-1TOa | GIGSILGVIAKGLPTLISWIKNR |
| Kasseptin 1Mb | FFGAIAAALPHVISAIKNAL |
| Sm Dermaseptin-like peptide, SmDLP | DLWNSIKDMAAAAGRAALNAVTGMVNQ |
| Heterin 2 | FWGALAKGALKLIPSLVSSFTKKD |
| Odorranain-P-RA1 peptide, Brevinin-1-OA1 | VIVFVASVAAEMMQHVYCAASKKC |
| Brevinin-1CG1 | FLSTALKVAANVVPTLFCKITKKC |
| Lactoferricin-B, Lfcin-B | FKCRRWQWRMKKLGAPSITCVRRAF |
| Brevinin-1CHb | FLPVIAGLAAKVLPKLFCAITKKC |
| Bactrocerin-1 | VGKTWIKVIRGIGKSKIKWQ |
| Caerin 1.9 | GLFGVLGSIAKHVLPHVVPVIAEKL |
| Css54 | FFGSLLSLGSKLLPSVFKLFQRKKE |
| Brevinin CTcu3 | LPLLAGLAANFLPKIFCKITRK |
| Antimicrobial peptide EcAMP2.1 | DRCSQQCQHHRDPDRKQQCMRECRRH |
| Brevinin-1Pd | FLPIIASVAANVFSKIFCAISKKC |
| Brevinin-1Yb | FLPIIAGAAAKVVQKIFCAISKKC |
| Caerin-1.10 | GLLSVLGSVAKHVLPHVVPVIAEKL |
| Maximin 39 | GIGTKFLGGVKTALKGALKELAFTYVN |
| Brevinin-1RE1 | FLPGLECVSGKIVPTVFCAITRIC |
| Brevinin-1CG3 | FLSTLLNVASNVVPTLICKITKKC |
| Siamycin II | CLGIGSCNDFAGCGYAIVCFW |
| Ceratotoxin-like peptide Ctx-Ha | GWLDVAKKIGKAAFNVAKNFI |
| Hepcidin TH1-5 | GIKCRFCCGCCTPGICGVCCRF |
| Cathelicidin-6, Myeloid antibacterial peptide 27, BMAP-27 | GRFKRFRKKFKKLFKKLSPVIPLLHLG |
| Cathelicidin-6, Myeloid antibacterial peptide 27, BMAP-27 | GRFKRFRKKFKKLFKKLSPVIPLLHLG |
| Lycotoxin-2, M-lycotoxin-Hc2a | KIKWFKTMKSIAKFIAKEQMKKHLGGE |
| XPF-SE3 | GFWTTAAEGLKKFAKAGLASILNPK |
| Tu-AMP2 alpha chain | KSCCRNTTARNCYNVCRIPG |
| Brevinin-1VLe | FLPLIAGVAASILPKIFCFITKKC |
| Brevinin-1Pa | FLPIIAGVAAKVFPKIFCAISKKC |
| LSer-Proline-rich peptide 4, LSer-PRP4 | SWIKKDKFPSSTGPYNPNPPPPRF |
| Sviceucin | CVWGGDCTDFLGCGTAWICV |
| Polydim-I | AVAGEKLWLLPHLLKMLLTPTP |
| Polydim-I | AVAGEKLWLLPHLLKMLLTPTP |
| Antimicrobial peptide 2, XT-2, XPF-St1 | GVWSTVLGGLKKFAKGGLEAIVNPK |
| Im-5 | FLGSLFSIGSKLLPGVIKLFQRKKQ |
| CPF-SE1 | GFLGPLLKLGLKGVAKVIPHLIPSRQQ |
| Nigroain-B-MS1 | CVVSSGWKWNYKIRCKLTGNC |
| M-zodatoxin-Lt5a, Latarcin-5, Ltc-5 | GFFGKMKEYFKKFGASFKRRFANLKKRL |
| Hymenochirin-5Pg | ITIPPIVKDTLKKFIKGAISSVM |
| Brevinin-1RTb | FLGSLLGLVGKVVPTLFCKISKKC |
| TA1686 Hepcidin-25 / LEAP | DTHFPICIFCCGCCHRSKCGMCCKT |
| Pleurain-E1 antimicrobial peptide | AKAWGIPPHVIPQIVPVRIRPLCGNV |
| CPF-P4 | GFGSFLGKALKAALKIGANVLGGAPEQ |
| Specialicin | CLGVGSCVDFAGCGYAVVCFw |
| Rothein 2.1 | AGGLDDLLEPVLNSADNLVHGL |
| Brevinin-1JDa | FLPAVIRVAANVLPTVFCAISKKC |
| Hedyotide B2 | IQCGESCVWIPCISSAWGCSCKNKICSS |
| P28, Pep27, Pep27 protein | MRKEFHNVLSSGQLLADKRPARDYNRK |
| Turkey heterophil peptide THP3 | LSCKRGTCHFGRCPSHLIKGSCSGG |
| Brevinin-1DYc | FLPLLLAGLPKLLCLFFKKC |
| Spinulosain-A1 | AMPWRPATGLLPIKPTHIKPLCGDD |
| Maximin 68 | GIGGALLSAGKAALKGLAKVLV |
| Brevinin-1TSa | FLGSIVGALASALPSLISKIRN |
| Catesbeianin-1 | MMRVMRRKTKVIWEKKDFIGLYSID |
| Brevinin-1DY1 | FLPLLAGLAANFLPTIICKIARKC |
| Isracidin, Alpha-S1-casein | RPKHPIKHQGLPQEVLNENLLRF |
| Brevinin-1AUa | FLPILAGLAAKLVPKVFCSITKKC |
| Brevinin-1HSa | FLPAVLRVAAKIVPTVFCAISKKC |
| Alyteserin-1b | GLKEIFKAGLGSLVKGIAAHVAN |
| Cathelicidin Gg-CATH7 | KRVNWRKVGLGASYVMSWLG |
| Ps Hepcidin | QSHLSLCRYCCNCCRNKGCGYCCKF |
| CPF-M1 | GLGSLLGKAFKFGLKTVGKMMAGAPREQ |
| Figainin 2, Figainin-02 | FLGAILKIGHALAKTVLPMVTNAFKPKQ |
| Daiyunin-2 | FFGTKGIFSKVEPIFCKISHSC |
| PGLa-AM2, PGLa-BM3 | GMASTAGSVLGKLAKAVAIGAL |
| Brevinin-1BYc | FLPILASLAATLGPKLLCLITKKC |
| Caerin-1.17 | GLFSVLGSVAKHLLPHVAPIIAEKL |
| Saha-CATH6, Saha cathelicidin 6 | KRIRFFERIRDRLRDLGNRIKNRIRDFFS |
| Kalata B7 | GLPVCGETCTLGTCYTQGCTCSWPICKRN |
| Brevinin1 HYba1 Cyclic AMD | FFPGIIKVASAILPTAICAITKRC |
| Pseudin-2 | GLNALKKVFQGIHEAIKLINNHVQ |
| Brevinin-2TEa | GIGSMLLGLAKNVGMSLLNKAQCKISGKC |
| Brevinin-1TEa | FFGPLIKIATGVLPNLICKALGKC |
| M-zodatoxin-Lt4b, Latarcin-4b, Ltc-4b | SLKDKVKSMGEKLKQYIQTWKAKF |
| Muscin | EWKLPDLIINHITLTRRNCFKYRCG |
| Maximin 31 | GIGGALLSAGKSALKGLAKGLAEHF |
| CPF-C2 | GLGSLLGKALKFGLKAAGKFMGGEPQQ |
| Grammistin C, Gs C | NWRKILGKIAKVAAGLLGSMLAGYQV |
| Raniseptin-3 | AWLDKLKSIGKVVGKVAIGVAKNLLNPQ |
| Pyrrhocoricin | VDKGSYLPRPTPPRPIYNRN |
| Ranatuerin-2ZHa | GLADYWRTAFRANFANLGPGIRCKSARC |
| Ixosin | GLHKVMREVLGYERNSYKKFFLR |
| Ixosin amide | GLHKVMREVLGYERNSYKKFFLR |
| XPF-St4 | GWASSIGSILGKFAKGGAQAFLQPK |
| Brevinin-1VLc | FLPVIASVAAKVLPKVFCFITKKC |
| Capitellacin | SPRVCIRVCRNGVCYRRCWG |
| Dermaseptin-1, DPh-1, DShypo 01, Dermaseptin-H4, DMS4 | GLWSTIKNVGKEAAIAAGKAALGAL |
| Brevinin-1TP4 | FLPGLIKAAVGIGSTIFCKISRKC |
| Cyclotide 7, Viba 15 | GLPVCGETCVGGTCNTPGCACSWPVCTRN |
| Brevinin-Bd | FLPAIAGVAAKFLPKIFCAISKKC |
| XPF-SE1 | GLFLDTLKKFAKAGMEAVTNPK |
| Cycloviolin B | GTACGESCYVLPCFTVGCTCTSSQCFKN |
| Nigrocin-1-OA3 | GIFLKVLGVGKKVLCGVSGLC |
| Brevinin-1CHa | FLPIIAGVAAKVLPKLFCAITKKC |
| Cereucin XA, CexA | MGKKIGKWIITGAAGWAGWEIGEGIWK |
| Pleskein-2 | FFLLPIPNDVKCKVLGICKS |
| Brevinin-1PMa | FLPLIAGVAAKVLPKIFCAISKKC |
| Maculatin-1.2 | GLFGVLAKVASHVVPAIAEHFQA |
| Nigrocin-2ISa | GIFSTVFKAGKGIVCGLTGLC |
| Frenatin 4.2 | GFLEKLKTGAKDFASAFVNSIK |
| Caerin-4.1 | GLWQKIKSAAGDLASGIVEGIKS |
| Maximin H46 | ISGPVLGLVGNALGGLIKKI |
| Cereucin HA, CehA | MAKIGKWVVKGAAGYLGWEIGEGIWK |
| Brevinin-1CG5 | FLPMLAGLAANFLPKIVCKITKKC |
| Odorranain-F-OA4 | GFMATAKNVAKNMDVTLLDNLKCKITKAC |
| Antimicrobial peptide PGQ, Preproprotein pGQ | GVLSNVIGYLKKLGTGALNAVLKQ |
| Frenatin-4 | GFLDKLKKGASDFANALVNSIKGT |
| Ranalexin | FLGGLIKIVPAMICAVTKKC |
| M-poneritoxin-Nc2a | FVKELWDKVKKMGSAAWSAAKGAFA |
| Ocellatin-2I | GLLDFFKGAGKELLTHLASQI |
| Nigrocin-1-OW2 | GILGNIVGMGKKIVCGLSGLC |
| Cathelicidin-PY | RKCNFLCKLKEKLRTVITSHIDKVLRPQG |
| Hepcidin TH2-3 | QSHLSLCRWCCNCCRSNKGC |
| Ranatuerin-2PLe | GIMDSVKNAAKNLAGQLLDTIKCKITAC |
| Dermaseptin-AC4, DRP-AC4 | SLWGKLKEMAAAAGKAALNAVNGLVNQ |
| Phenol-soluble modulin alpha 2 peptide, psmA2 | MGIIAGIIKFIKGLIEKFTGK |
| Brevinin-1LF2 | FLPIVASLAANFLPKIICKITKKC |
| Caerin-2.2 | GLVSSIGRALGGLLADVVKSKEQPA |
| Picturin-2 | GVFKDALKQFGAALLDKAANALKPK |
| Cyclotide Vigno 5 | GLPLCGETCVGGTCNTPGCSCGWPVCVRN |
| Parkerin | GWANTLKNVAGGLCKITGAA |
| Cyclotide vibi-D | GLPVCGETCFGGRCNTPGCTCSYPICTRN |
| Brevinin-1GHa | FLGAVLKVAGKLVPAAICKISKKC |
| mBjAMP1 | NLCASLRARHTIPQCKKFGRR |
| Lividin-5, Ranacyclin-B-RL1 | AALRGCWTKSIPPKPCPGKR |
| M-zodatoxin-Lt3a, Latarcin-3a, Ltc-3a, La47 | SWKSMAKKLKEYMEKLKQRA |
| Kalata B2 | GLPVCGETCFGGTCNTPGCSCTWPICTRD |
| Dermaseptin-5, Dermaseptin-S5 | GLWSKIKTAGKSVAKAAAKAAVKAVTNAV |
| Meucin-24 | GRGREFMSNLKEKLSGVKEKMKNS |
| Phenol-soluble modulin alpha 1 peptide, PSMalpha1 | MGIIAGIIKVIKSLIEQFTGK |
| Dermaseptin-PS4, Der-PS4 | ALWKTLLKHVGKAAGKAALNAVTDMVNQ |
| Maximin H23 | ILGPVISTIGNVLGGLLKNL |
| Cyclotide chassatide C11, Uncyclotide ChaC11 | IPCGESCVWIPCISGMFGCSCKDKVCYS |
| Maculatin 1.3 | GLLGLLGSVVSHVVPAIVGHF |
| Hymenochirin-5Pb | FKIPPIVKDTLKKFFKGGIAGVMGQ |
| CPF-SP1 | GFLGPLLKLGLKGVAKVLPHLIPSRQQ |
| Sm-Piscidin | KGARQAWKDYKYNRNMQKMNQGYGQQGG |
| Alyteserin-1a | GLKDIFKAGLGSLVKGIAAHVAN |
| XPF-SP1 | GFWSSALEGLKKFAKGGLEALTNPK |
| Antimicrobial peptide 4, XT-4 | GVFLDALKKFAKGGMNAVLNPK |
| Brevinin-1HB1 | FLPAIIGMAAKVLPAFLCKITKKC |
| Ranacyclin-B-LK1 | SALVGCWTKSWPPKPCFGRG |
| Hymenochirin-3B | IKIPAVVKDTLKKVAKGVLSAVAGALTQ |
| Dermadistinctin-Q2, DD Q2 | GLWSKIKEAAKTAGLMAMGFVNDMV |
| Lasiocepsin | GLPRKILCAIAKKKGKCKGPLKLVCKC |
| Lasiocepsin AMD | GLPRKILCAIAKKKGKCKGPLKLVCKC |
| Maximin 32 | GIGGKILGGLKTALKGAAKELASTYLH |
| Odorranain-W1 | GLFGKSSVWGRKYYVDLAGCAKA |
| Bombinin-like peptide 2, BLP-2 | GIGSAILSAGKSALKGLAKGLAEHFAN |
| Brevinin-1ITa | IVPFLLGMVPKLVCLITKKC |
| Ps Hepcidin | AFKCKFCCGCCRAGVCGLCCRF |
| Brevinin-1PTa | FMGGLIKAATKIVPAAYCAITKKC |
| Brevinin-1TP1 | FLPGLIKAAVGVGSTILCKITKKC |
| Hymenochirin-5Pc | ITIPPIIKDTLKKFFKGGIAGVMGKSQ |
| Brevinin-1SY, Brevinin-1S, RaCa15 | FLPVVAGLAAKVLPSIICAVTKKC |
| MCh-AMP1 | LSVKAFTGIQLRGVCGIEVKARG |
| Ponericin-W4 | GIWGTALKWGVKLLPKLVGMAQTKKQ |
| Hymenochirin-5Pd | ITIPPIVKDTLKKFFKGGIAGVMGQ |
| Cyclotide mech-3 | GLPTCGETCTLGKCNTPKCTCNWPICYKN |
| Varv peptide A | GLPVCGETCVGGTCNTPGCSCSWPVCTRN |
| Ranatuerin-2PRc | GILDSFKDVAKGVATHLLNMAKCKMTGC |
| Xenopsin precursor fragment-amide (XPF-amide) | GWASKIGQTLGKIAKVGLKELIQPK |
| Brevinin-1-RAA10 antimicrobial peptide, Brevinin-1-OA12 | FLPAVIRVAANVLPTAFCAISKKC |
| SmHep2P, Sm-Hepcidin-2 | GMKCKFCCNCCNLNGCGVCCRF |
| Brevinin 1-Ia | FLGALLKIGAKVLPSVLCGIFKKC |
| Ulmin-1ULa | LALRTAGWLRLLGFRDKKKN |
| Brevinin-1-OR11 | LAFVAGVAAEMMQHVYCAASKKC |
| Dermaseptin-SP5 | SLRSSIKDMAAAAGRAALNAVNGIVNP |
| XPF-AN1, XPF-R2 | GWASKIGQTLGKMAKVGLQELIQPK |
| Vasoactive intestinal peptide, VIP | HSDAVFTDNYTRLRKQMAVKKYLNSILN |
| Ranatuerin-2Ya | GLMDTIKGVAKTVAASWLDKLKCKITGC |
| Cycloviolacin O16 | GLPCGETCFTGKCYTPGCSCSYPICKKIN |
| Brevinin-1JDc | FLPAVLRVAAKVVPTVFCLISKKC |
| Kenojeinin I | GKQYFPKVGGRLSGKAPLAAKTHRRLKP |
| Maximin 63 | GIGGVLLGAGKATLKGLAKVLAEKYAN |
| Dermal antimicrobial peptide Hs-1 | FLPLILPSIVTALSSFLKQG |
| Maximin 15 | GIGTKILGGVKAALKGALKELASTYVN |
| Ranatuerin-2YJ | GLMDIFKVAVNKLLAAGMNKPRCKAAHC |
| Psyle A | GIACGESCVFLGCFIPGCSCKSKVCYFN |
| Wuchuanin-E1 | CVDIGFSPTGKRPPFCPYPG |
| Caerin-1.3 | GLLSVLGSVAQHVLPHVVPVIAEHL |
| Magainin-M1 | GIGKFLHSAGKFGKAFIGEIMKS |
| brevinin-1ZHa | FLPFLLSALPKVFCFFSKKC |
| Nigroain-A antimicrobial peptide, Ranacyclin-B-RN6 | SALVGCWTKSYPPNPCFGRG |
| Pleurain-a1-thel | RILTMTKRVKMPQLYKQIVCRLFKTC |
| Brevinin-1GHd, Brevinin-1HL | FLGALFKVASKLVPAAICSISKKC |
| Taipehensin-1TP1 | TLIWEFYHQILDEYNKENKG |
| Brevinin-1-OR3 | IDPFVAGVAAEMMQHVYCAASKKC |
| Cyclotide chassatide C1 | GDACGETCFTGICFTAGCSCNPWPTCTRN |
| Brevinin-Eu | VIPFVASVAAEMMQHIFCAASRKC |
| Truncated apolipoprotein C-Ia, APOC1(67-88), Apo6 | KTRNWFSEHFKKVKEKLKDTFA |
| Varv peptide D | GLPICGETCVGGSCNTPGCSCSWPVCTRN |
| Palustrin-1c | ALSILRGLEKLAKMGIALTNCKATKKC |
| Dermadistinctin-L, DD L | ALWKTLLKNVGKAAGKAALNAVTDMVNQ |
| Proline-rich peptide SP-B | APPGARPPPGPPPPGPPPPGP |
| Ranacyclin-HB1 | GAPKGCWTKSYPPQPCFGKK |
| Maximin 42 | SIGAKILGGVKTFFKGALKELAFTYLQ |
| Ishikawain-8 | GIFSVLNEVCKKNDYKPEICAHFSQNKP |
| D-L-Peptide 73, D-L73 | GPlGvRGKRLWDIVRRWVGWL |
| Magainin-AN2, Magainin-AM2, magainin-F3, Magainin-BM2, Magainin-R3 | GVSKILHSAGKFGKAFLGEIMKS |
| Japonicin-2 | FGLPMLSILPKALCILLKRKC |
| Pseudin-1 | GLNTLKKVFQGLHEAIKLINNHVQ |
| Odorranain-F1 | GFMDTAKNAAKNVAVTLLDNLKCKITKAC |
| Enterocin NKR-5-3D | TPGGIDFISGGPHVAQDVLNAIKNFFK |
| Gaegurin-RN1 | FIGPVLKIAAGILPTAICKIFKKC |
| Maximin H39 | ILGPVLGLVGNALGGLIKKL |
| XPF-F1 | GWASKIGQTLGKMAKVGLQEIIQPK |
| Gaegurin-LK2 | FLGPIIKMATGILPTAICKGLKKC |
| Magainin-2 | GIGKFLHSAKKFGKAFVGEIMNS |
| Hepcidin-20 | ICIFCCGCCHRSKCGMCCKT |
| Brevinin-1Pc | FLPIIASVAAKVFSKIFCAISKKC |
| Plantaricin ZJ5 | KTKQQFLIKAQTQLFKVFGYTL |
| Thaulin-1 | NGNLLGGLLRPVLGVVKGLTGGLGKK |
| Cyclotide mech-2 | GLPTCGETCTLGKCNTPKCTCNWPICYKD |
| Dermaseptin-like PBN2 | GLVTSLIKGAGKLLGGLFGSVTGGQS |
| Pleurain-A2 | SIITMTKEAKLPQSWKQIACRLYNTC |
| Cruzioseptin-3, CZS-3 | GFLDVVKHIGKAALGAVTHLINQ |
| Dermaseptin-AC | GMFTNMLKGIGKLAGKAALGAVKTLA |
| Dermaseptin t-DPH1 | GLWSKIKNVAAAAGKAALGAL |
| Brevinin-1CDYa | LLSLALAALPKLFCLIFKKC |
| Caerin 1.19 | GLFKVLGSVAKHLLPHVAPIIAEKL |
| Peptide DK25 | DVNDLKNLCAKTHNLLPMCAMFGKK |
| Enkelytin | FAEPLPSEEEGESYSKEPPEMEKRYGGFM |
| Vitri E, Cyclotide C | GLPVCGETCVGGTCNTPGCSCSWPVCFRN |
| Arcumycin | CLPSGDCPDFLGCGRAIWCI |
| PGLa-LM1 | GMASKAGSVAGKIAKFALGAL |
| Palustrin-2AJ1 | GFMDTAKNVAKNVAVTLIDKLRCKVTGGC |
| Scolopin 2 | GILKKFMLHRGTKVYKMRTLSKRSH |
| Ponericin-W1 | WLGSALKIGAKLLPSVVGLFKKKKQ |
| CPF-MW2 | GLGSLLGKAFKFGLKTVGKMMGGAPREE |
| Gaegurin-RN5 | FLGPIIKIATGILPTAICKFLKKC |
| Nigroain-K1 | SLWETIKNAGKGFIQNILDKIR |
| Brevinin-1La, Brevinin-1PRd | FLPMLAGLAASMVPKLVCLITKKC |
| Brevinin-1OS | FLPGILKVAANVVPGVICAITKKC |
| Ranatuerin-2TRa | GIMDSIKGAAKEIAGHLLDNLKCKITGC |
| Nigrocin-2L, Nigrocin-2LVb | GILSGILGMGKKLVCGLSGLC |
| Gaegurin-RN4 | FVGPVLKIAAGILPTAICKIYKKC |
| Alyteserin-1c | GLKEIFKAGLGSLVKGIAAHVAS |
| Brevinin-1TP2 | FLPGLIKAAVGIGSTIFCKISKKC |
| Frenatin-3 | GLMSVLGHAVGNVLGGLFKPKS |
| Maculatin-3.1 | GLLQTIKEKLESLESLAKGIVSGIQA |
| Bombinin-like peptide 3, BLP-3 | GIGAAILSAGKSALKGLAKGLAEHF |
| Tilapia piscidin 5, TP5 | QLQGKQVSGEVVQKVLQELIQSVAKP |
| PGLa-B2 | GMASKAGSIVGKIAKIALGAL |
| Dermaseptin-SP3 | SLWSSIKDMAAAAGRAALNAVNGIVNP |
| Pilosulin 3 | IIGLVSKGTCVLVKTVCKKVLKQ |
| Ocellatin-PT5 | GVFDIIKDAGRQLVAHAMGKIAEKV |
| Cruzioseptin-17, CZS-17 | GFLDVVKGVGKAALGAVTHLINQGEQ |
| Dermaseptin-PT9 | GLWSKIKDAAKTAGKAALGFVNEMV |
| Pentadactylin, Ocellatin-P1 | GLLDTLKGAAKNVVGSLASKVMEKL |
| Cathelicidin-RC1 | KKCKFFCKVKKKIKSIGFQIPIVSIPFK |
| Ceratotoxin-like peptide Ctx-Ha | GWLDVAKKIGKAAFNVAKNFL |
| Brevinin-1H | FALGAVTKVLPKLFCLITRKC |
| Magainin-C1 | GVGKFLHSAKKFGQALASEIMKS |
| Brevinin-1SN2 | FMGTALKIAANVLPAAFCKIFKKC |
| Odorranain-L2 antimicrobial peptide, Odorranain-L1 | VEVQVRDKGKGIYGLSPLRQPAP |
| Dermaseptin DRS-CA-1 | ALWKDLLKNVGKAAGKAVLNKVTDMVNQ |
| Brevinin-1-OR8 | ILPFVAGVAAEMMEHVYCAASKKC |
| Odorranain-NR, OGG1 antimicrobial peptide | GLLSGILGAGKHIVCGLTGCAKA |
| Andersonin-M3 peptide, Andersonin-H3 | VAIYGRDDRSDVCRQVQHNWLVCDTY |
| Maximin 49 | GIGGVLLSAGKAALKGLTKVLAEKYAN |
| Nigrocin-OG4 | GLLSGILGAGKHIICGLSGLC |
| Ocellatin-1I | GLLDLLKGAGKGLLTHLASQI |
| Delta-lysin I | MAADIISTIGDLVKLIINTVKKFQK |
| Ranatuerin-2PRh | GILDTVKGVAKDVAAHLLNMVKCKITGC |
| Hymenochirin-1Pa | LKLSPKTKDTLKKVLKGAIKGAIAIASMA |
| Cruzioseptin-16, CZS-16 | GFLDVLKGVGKAALGAVTHLINQGEQ |
| Caerin-3.1 | GLWQKIKDKASELVSGIVEGVK |
| Bombinin-H2 | IIGPVLGLVGSALGGLLKKI |
| Sm-NK-lysin | GLLKSLCRKFVKVHLPELIEELTTTD |
| Nigrocin-2JDb | GIFGKILGVGKKVLCGLSGMC |
| Ranatuerin-2PRa | GLMDVFKGAAKNLLASALDKIRCKVTKC |
| Nigrocin-2HSa | GLLGSLFGAGKKVACALSGLC |
| Brevinin-1Yc | FLPIIAGAAAKVVEKIFCAISKKC |
| Phenol soluble modulin alpha, PSMa | MADVIAKIVEIVKGLIDQFTQK |
| Cyclotide mela-6 | GLPTCGETCFKGKCYTPGCSCSYPICKKD |
| Maximin-1 | GIGTKILGGVKTALKGALKELASTYAN |
| Somuncurin-4.3 | NNEENELRRRVSFNRAVIHSLLG |
| CPF-SE2 | GFLGPLLKLGLKGAAKLLPQLLPSRQQ |
| Thanatin -AMD | GSKKPVPIIYCNRRTGKCQRM |
| Peptide DF22 | DVNDLKNLCAKTHNLLPMCAMF |
| Ranatuerin-2TOa | GLLNVIKDTAQNLFAAALDKLKCKVTKCN |
| Ocellatin-V3 | GVLDILTGAGKDLLAHALSKLSEKV |
| Grammistin Pp 3 | NWRKILGQIASVGAGLLGSLLAGYE |
| PGLa-like peptide, Antimicrobial peptide 5, XT-5, PGLa-SP1 | GMATKAGTALGKVAKAVIGAAL |
| Ranatuerin-2PLc, Ranatuerin-2Va | GLLDTIKNTAKNLAVGLLDKIKCKMTGC |
| Saha-CATH5, Saha cathelicidin 5 | KRIGLIRLIGKILRGLRRLG |
| Cruzioseptin-2, CZS-2 | GFLDVIKHVGKAALGVVTHLINQ |
| Nigrocin-1-OR2 | GILSGILGVGKMLVCGLSGLC |
| CPF-P3 | GFGSFLGKALKAALKIGANVLGGAPQQ |
| Odorranain-F-OA1 | GFMDTAKNVAKNMAGNLLDNLKCKITKAC |
| Hymenochirin-5Pe | ITIPPIVKDTLKKFIKGAISGVM |
| Brevinin-2-related peptide B-GR23-2, 2GHh | GVITDALKGAAKTVAAELPRKAH |
| Ponericin-L1, U1-poneritoxin-Ng2a, Bmâ€‘ponericinâ€‘L1 | LLKELWTKMKGAGKAVLGKIKGLL |
| Pseudin B | GLNTLKKVIQGLHEVIKLVNNHA |
| Ranalexin-Vb | FLGGLFKLVPSVICAVTKKC |
| Maximin 45 | GIGGKILGGLRTALKGAAKELAATYLH |
| Pictuseptin-3 | GFLDTLKNIGKTVGKVALDVAKNVLT |
| Nigrocin-2ISb | GILGTVFKAGKGIVCGLTGLC |
| Dermaseptin-PC, DM-PC | ALWKSILKNVGKAAGKAVLNAVTDMVNQ |
| Hymenochirin-5B | IKIPPIVKDTLKKVAKGVLSTIAGALST |
| XPF-B2 | GWASKIGTQLGKMAKVGLKEFVQS |
| Pleurain-D4 antimicrobial peptid | FRSGILKLASKIPSVLCAVLKNC |
| Ranatuerin-2SN1 | GFLNTAMNTVTNLAGTLMDKAKCKIRGC |
| Nigrocin-2SCc | GILSNVLGMGKKIVCGLSGLC |
| Bombinin-BV | GIGGALLNVGKVALKGLAKGLAEHFAN |
| Hylaseptin-4 | GIGDILKNLAKAAGKAALHAVGESL |
| Raniseptin-6 | ALLDKLKSLGKVVGKVALGVVQNYLNPRQ |
| PGLa-SE2 | GMATAAGTTLGKLAKFVIGAV |
| M-myrmicitoxin(01)-Tb1a, TeBi1 | KIKIPWGKVKDFLVGGMKAVGKK |
| M-myrmicitoxin(01)-Tb1a, Bicarinalin, P16 | KIKIPWGKVKDFLVGGMKAV |
| Ranatuerin-2CHb | GLMDTIKGVAKNVAASLLEKLKCKVTGC |
| Maximin-H7 | ILGPVIKTIGGVIGGLLKNL |
| Maximin H2 | ILGPVLSMVGSALGGLIKKI |
| Panurgine K, PNG-K | LDVKKIICVACKIKPNPACKKICPK |
| Cyclotide mela-1 | GKYTCGETCFKGKCYTPGCTCSYPICKKD |
| Ponericin-G4 | DFKDWMKTAGEWLKKKGPGILKAAMAAAT |
| Ranatuerin-2SEa, Palustrin-2AR | GFISTVKNLATNVAGTVIDTIKCKVTGGC |
| Brevinin-2 related peptide B2RP | GIWDTIKSMGKVFAGKILQNL |
| Ranatuerin-1, Ranatuerin-1CBa | SMLSVLKNLGKVGLGFVACKINKQC |
| Omega-conotoxin MVIIA, Ziconotide | CKGKGAKCSRLMYDCCTGSCRSGKC |
| Brevinin 2R | KLKNFAKGVAQSLLNKASCKLSGQC |
| HsAp | SGTSEKERESGRLLGVVKRLIVCFRSPFP |
| Ocellatin-4 | GLLDFVTGVGKDIFAQLIKQI |
| Palustrin-1d | ALSILKGLEKLAKMGIALTNCKATKKC |
| Maximin 4 | GIGGVLLSAGKAALKGLAKVLAEKYAN |
| Skh-AMP1 | GRTSKQELCTWERGSVRQADKTIAG |
| Alyteserin-1Ma, Preproalyteserin-1/2Ma | GFKEVLKADLGSLVKGIAAHVAN |
| Ganodermin | AGETHTVMINHAGRGAPKLVVGGKKLS |
| Nigrosin-RA3 antimicrobial peptide, Nigrocin-1-OA1 | GLLSGVLGVGKKIVCGLSGLC |
| LSEI_2386, m2386 | DSIRDVSPTFNKIRRWFDGLFK |
| Bombinin-H1 | IIGPVLGMVGSALGGLLKKI |
| Maximin-H3 | ILGPVLGLVGNALGGLIKKI |
| Dermaseptin-H5, Dermaseptin-like peptide 5, DMS5 | GLWSTIKNVGKEAAIAAGKAVLGSL |
| Dermaseptin-PS3, DPS3, Dermaseptin-4 | ALWKDILKNAGKAALNEINQIVQ |
| Nigrocin-1-OW3 | GILGNIVGMGKKVVCGLSGLC |
| Tk-AMP-X2 | ADDRCERMCQRYHDRREKKQCMKGCRYG |
| Dermaseptin-PS1 | GLWKSLFKNVGKAAGKAALNAVTDMVNQ |
| Frenatin 4.1 | GFLEKLKTGAKDFASAFVNSIKGT |
| Bacteriocin BaCf3 | GNHDCCMGQLPKCMLGPGQGAATCQGK |
| Nigrocin-1-OA2 | GIFGKILGVGKKTLCELSGMC |
| Dermaseptin-SP2 | ASWKVFLKNIGKAAGKAVLNSVTDMVNQ |
| Nigrocin-2ISc | GILSTVFKAGKGIVCGLSGLC |
| Maximin H54 | ILGPVLGLDGNALGGLIKKI |
| Caerin 2.1 | GLVSSIGRALGGLLADVVKSKGQPA |
| Prepromelittin-related peptide, MRP, AR-23 | AIGSILGALAKGLPTLISWIKNR |
| Hymenochirin-1B | IKLSPETKDNLKKVLKGAIKGAIAVAKMV |
| Hymenochirin-12B | LKIPGFVRDTLKKVAKEIFSAVTGAVTQS |
| Jindongenin-1a | DSMGAVKLAKLLIDKMKCEVTKAC |
| Nigrocin-2 | GLLSKVLGVGKKVLCGVSGLC |
| Chensinin-3CE, Chensinin-2CE | NFAEIFAAVNKLIKQGVVKG |
| Palustrin-2ISc, Lividin-11, Odorranain-F-OR1 | GFMDTAKNVAKNVAATLLDKLKCKITGGC |
| Nigrosin-OG20, Grahamin-1 | GLLSGILGAGKHIVCGLSGLC |
| Andersonin-R1 | ENAEEDIVLMENLFCSYIVGSADSFWT |
| CPF-P2 | GLASFLGKALKAGLKIGSHLLGGAPQQ |
| M-myrmeciitoxin-Mp2b | LIGLVSKGTCVLVKTVCKKVLKQ |
| Odorranain-F-OA3 | GFMDTAKNVAKNEAGNLLDNLKCKITKAC |
| Hymenochirin-10B | IKIPPIVKDTLKKVAKGVLSTVADALSKS |
| Dermaseptin-B4, Dermaseptin-2, Dermaseptin BIV | ALWKDILKNVGKAAGKAVLNTVTDMVNQ |
| Maximin H1 | ILGPVISTIGGVLGGLLKNL |
| Plasticin-TR | GLVSGLLNSVTGLLGNLAGGGL |
| Palustrin-2ISa | GFMDTAKNVAKNVAVTLLDKLKCKITGGC |
| Andersonin-X1 | GLFSKFAGKGIVNFLIEGVE |
| Ranatuerin-2PRe | GIMNTVKDVATGVATHLLNMVKCKITGC |
| Scolopin 1 | FLPKMSTKLRVPYRRGTKDYH |
| Nigrocin-2GRa, Grahamin-1 | GLLSGILGAGKNIVCGLSGLC |
| Odorranain-H2 antimicrobial peptide, Odorranain-H1 | GIFGKILGVGKKVLCGLSGVC |
| Ocellatin-PT2 | GVFDIIKDAGKQLVAHATGKIAEKV |
| Dermaseptin-like PBN2, PBN2 KF | GLVTSLIKGAGKLLGGLFGSVTG |
| Nigrocin-2JDa | GIFGKILGAGKKVLCGLSGLC |
| Ranatuerin-1C | SMLSVLKNLGKVGLGLVACKINKQC |
| Caerin-4.3 | GLWQKIKQAAGDLASGIVEGIKS |
| Magainin-C2 | GVGKFLHSAKKFGQALVSEIMKS |
| Acipensin 6, Ac6 | ILELAGNAARDNKKTRIIPRHLQL |
| Nigrosin-2Sb, Nigrocin-1-OW1 | GILSGVLGMGKKIVCGLSGLC |
| Bombinin-like peptide 7, BPL-7 | GIGGALLSAGKSALKGLAKGLAEHFAN |
| Maximin-H5 | ILGPVLGLVSDTLDDVLGIL |
| Lactoferricin B like peptide, LBLP | RMKKLGNHKVSCERNTKRCRKAI |
| Dermaseptin PD-3-6, PD36 | GVVTDLLNTAGGLLGNLVGSLSG |
| dahlein 5.6 | GLLASLGKVFGGYLAEKLKPK |
| Hymenochirin-1Pb | LKLSPETKDTLKKVLKGAIKGAIAIASLA |
| Insulin A chain | GIVEQCCASVCSLYQLENYCN |
| Dermaseptin-TO, Dermaseptin-1 | ALWKDLLKNVGIAAGKAALNKVTDMVNQ |
| Nigrosin-OG21 antimicrobial peptide | GLLSGVLGVGKKVLCGLSGLC |
| Syphaxin | GVLDILKGAAKDLAGHVATKVINKI |
| Andersonin-R peptide, Hejiangin-E1 | SADQTGMNKAALSPIRFISKSV |
| Kasstasin | FIKELLPHLSGIIDSVANAIK |
| Citropin-2.1 | GLIGSIGKALGGLLVDVLKPKL |
| Palustrin-2HB1 | GLWTTIKEGLKKFSLGVLDKIRCKIAGGC |
| CPF-P5 | GFGSFLGKALKAALKIGADVLGGAPQQ |
| Nigrocin-OG5 | GLLSGILGAGKQKVCGLSGLC |
| Ocellatin-PT4 | GVFDIIKGAGKQLIAHAMGKIAEKV |
| Hymenochirin-7B | KIPPVVKDTLKKVAKGVLSAVAGALS |
| Odorranain-Q2 antimicrobial peptide | APFCLGYLSPKLKDMEPKPRG |
| Hymenochirin-6B | IKVPAIVKDTQKKVAKGVISTVADALSKS |
| Dermaseptin DRS-DU-1 | ALWKSLLKNVGKAAGKAALNAVTDMVNQ |
| Citropin-2.1.3 | GLIGSIGKALGGLLVDVLKPKLQAAS |
| Brevinin-2-related peptide B-GR23-1, 2GHd | GLVTDTLKGAAKTVAAELLRKAH |
| Maximin H37 | ILGPVLGLVSNTLDDVLGIL |
| Lividin-7c protein, Lividin-7a, Nigrocin-1-OR1 | GILSGILGVGKKLVCGLSGLC |
| Nigrosin-OG13 | GLLSGILGAGKHIVCGLSGLR |
| Nigrocin-2S, Nigrocin-2SCa | GILSGILGAGKSLVCGLSGLC |
| Plasticin-L1 | GLVNGLLSSVLGGGQGGGGLLGGIL |
| Leptoglycin | GLLGGLLGPLLGGGGGGGGGLL |
| Ocellatin-V2 | GVLDILKGAGKDLLAHALSKISEKV |
| Odorranain-F-RA1 antimicrobial peptide, Odorranain-F-OA2 | GFMDTAKNVAKNMAVTLLDNLKCKITKAC |
| Laticeptin, Ocellatin-L1 | GVVDILKGAAKDLAGHLATKVMNKL |
| Ranatuerin-2Cb | GLFLDTLKGLAGKLLQGLKCIKAGCKP |
| XPF-St7 | GLLSNVAGLLKQFAKGGVNAVLNPK |
| M-myrmeciitoxin-Mp2a | IDWKKVDWKKVSKKTCKVMLKACKFL |
| Ranatuerin-2PLf | GIMDTVKNAAKDLAGQLDKLKCRITGC |
| Cryptonin | GLLNGLALRLGKRALKKIIKRLCR |
| Picturin-1 | GVFKDALKQLGAALLDKAANALKPK |
| Rothein 2.2 | AGDDETLLKPVLNSLDNLVSGL |
| Aurein-5.2 | GLMSSIGKALGGLIVDVLKPKTPAS |
| Nigrosin-2V, Nigrocin-2VB | SILSGNFGVGKKIVCGLSGLC |
| Alyteserin-1Mb | GFKEVLKAGLGSLVKGIPAHVAN |
| Nigrocin-2GRb, Odorranain-H1 | GLFGKILGVGKKVLCGLSGMC |
| Ocellatin-V1 | GVVDILKGAGKDLLAHALSKLSEKV |
| Ranatuerin-2B | GLLDTIKGVAKTVAASMLDKLKCKISGC |
| SAMPH1, Histone H1 | AEVAPAPAAAAPAKAPKKKAAAKPKKAGPS |
| Maximin 41 | GIGGALLSVGKSALKGLTKGLAEHF |
| Pictuseptin-2 | GFLDTLKNIGKTVGGIALNVLT |
| Caerin-2.6 | GLVSSIGKVLGGLLADVVKSKGQPA |
| Spinigerin | HVDKKVADKVLLLKQLRIMRLLTRL |
| Dermaseptin-PH | ALWKEVLKNAGKAALNEINNLVQ |
| Distinctin chain 1 | ENREVPPGFTALIKTLRKCKII |
| Caerin-3.5 | GLWEKVKEKANELVSGIVEGVK |
| Nigrocin-AA1 | GLLSGILGAGKRIVCGLSGLC |
| Ranatuerin-4 | FLPFIARLAAKVFPSIICSVTKKC |
| Thaulin-Sl | DLLNGLLNPVLGIANGLTGGLVKK |
| Maximin-2 | GIGTKILGGVKTALKGALKELASTYVN |
| Ranatuerin-2PLd | GIMDSVKNVAKNIAGQLLDKLKCKITGC |
| Caerin-4.2 | GLWQKIKSAAGDLASGIVEAIKS |
| Ranatuerin-2PLx, R2PLx | GIMDTVKNAAKNLAGQLLDKLKCKITAC |
| P1-Hp-1971 | TKPTLLGLPLGAGPAAGPGKR |
| Splendipherin | GLVSSIGKALGGLLADVVKSKGQPA |
| Brevinin-1ZHb, Amurin-2b | FLPLLLSALPSFLCLVFKKC |
| Ranatuerin-2ARa | GLMDTVKNAAKNLAGQLLDTIKCKMTGC |
| Distinctin chain 2 | NLVSGLIEARKYLEQLHRKLKNCKV |
| Nigrocin-2HSb | GLLGSIFGAGKKIACALSGLC |
| PGLa-AN2 | GMASKAGSVLGKLAKVAIGAL |
| Ocellatin-PT3 | GVIDIIKGAGKDLIAHAIGKLAEKV |
| Ranatuerin-2PLb | GIMDTVKNAAKDLAGQLLDKLKCRITGC |
| Fallaxin (1-22), Ocellatin-LB1 | GVVDILKGAAKDIAGHLASKVM |
| Ranatuerin-2ARb | GILDTIKNAAKTVAVGLLEKIKCKMTGC |
| Ranatuerin-2ONa | GLMDTVKNAAKNLAGQMLDKLKCKITGSC |
| Nigrocin-PN | GLLGKILGAGKKVLCGVSGLC |
| Brevinin-2Va | GIWDTLKNVGKAVLGKVLENV |
| Rothein 3.1 | ASAAGAVRAGDDETLLKPVLNSLDNLVSGL |
| Ocellatin-LB2 | GVVDILKGAAKDIAGHLASKVMN |
| PGLa-MW1 | GMASKAGSVLGKITKIALGAL |
| Misgurin | RQRVEELSKFSKKGAAARRRK |
| FM-Cathelicidin | LKTKALNKLKQKLQAVGNLIGSVIKG |
| Monomeric Homotarsinin, Htr-M | NLVSDIIGSKKHMEKLISIIKKCR |
| Ranatuerin-2BYb | GIMDSVKGLAKNLAGKLLDSLKCKITGC |
| Ranatuerin-2PLa | GIMDTVKNVAKNLAGQLLDKLKCKITAC |
| Ocellatin-L2 | GVVDILKGAAKDLAGHLATKVMDKL |
| Fallaxin, Ocellatin-F1 | GVVDILKGAAKDIAGHLASKVMNKL |
| Andersonin-L peptide, Andersonin-G1 | KEKLKLKCKAPKCYNDKLACT |
| Palustrin-2DY1 | GIMDTIKNAAKDVVQSLLNKASCKLAKTC |
| Ocellatin-1N | GAVVDILKGAGKNLLSLALNKLSEKV |
| Panurgine R, PNG-R | LDVKKIICVACKIRPNPACKKICPK |
| Enkelytin | FAEPLPSEEEGEXYXKEVPEMEKRYGGFM |
| Palustrin-2GN3 | GILDTLKQLGKAAAQSLLSKAACKLAKTC |
| Nigrocin-RE1, Nigrocin-HL | GLLSGILGAGKKIVCGLSGLC |
| Caerin-3.2 | GLWEKIKEKASELVSGIVEGVK |
| Guentherin, AMP-3 | VIDDLKKVAKKVRRELLCKKHHKKLN |
| Antimicrobial peptide EP-20 | EGPVGLADPDGPASAPLGAP |
| Nigrocin-OG2 | GLLGKILGVEKKVLCGLSGMC |
| Musca domestica antifungal peptide-1, MAF-1A | KKFKETADKLIESAKQQLESLAKEMK |
| Andersonin-6 antimicrobial peptide, Andersonin-S1 | DANVENGEDAEDLTDKFIGLMG |
| Ranatuerin-2VLb | GIMDTIKGAAKDLAGQLLDKLKCKITKC |
| Cyclotide mela-4 | GKPICGETCFKGKCYTPGCTCSYPICKKN |
| Cyclotide mela-3 | GKPICGETCFKGKCYTPGCTCSYPICKKD |
| Antimicrobial peptides He-1 | DDDKTEEEDDKENETTKVVE |
