# Supplementary material for: From computational screening to in vitro validation: exploring antimicrobial peptides against Pseudomonas aeruginosa
Source: Front Microbiol. 2026 Jun 11;17:1796090. doi: 10.3389/fmicb.2026.1796090 (PMC13294467; doi:10.3389/fmicb.2026.1796090)
Supplement: Supplementary file 3 [file Table_3.DOCX]

Table 2. Physiological properties of the parent peptides along with the mutant peptides

| **PEPTIDE NAME** | **PEPTIDE SEQUENCE** | **LENGTH** | **HALF-LIFE [SEC]** | **STABILITY** | **HYDROPHOBICITY (KJ/MOL)** | **MOLECULAR WEIGHT** | **CHARGE** | **ISOELECTRIC POINT** | **RELATIVE STABILITY** | **CLASS** |
| --- | --- | --- | --- | --- | --- | --- | --- | --- | --- | --- |
| **Chicken CATH-2** | **RFGRFLRKIRRFRPKVTITIQGSARFG** | **27** | **0.0001** | **Low** | **10.989** | **3265.32** | **9** | **7.392** | **3.124** | **Non-CPP** |
| CC_11 | RFGRCCRKIRRFRPKVTITIQGSARFG | 27 | 0.135 | Normal | 11.374 | 3211.25 | 9 | 7.341 | 2.934 | **CPP** |
| CC_26 | RFGRFPRKIRRFRPKVTITIQGSARFG | 27 | 0.0001 | Low | 11.452 | 3249.27 | 9 | 7.404 | 3.04 | **Non-CPP** |
| CC_29 | RCGRFLRKIRRFRPKVTITIQGSARFG | 27 | 0.0001 | Low | 11.252 | 3221.28 | 9 | 7.376 | 3.027 | **CPP** |
| CC_34 | RFGRFLRKIRRFRPKVTITIQGSARFG | 27 | 0.0001 | Low | 10.989 | 3265.32 | 9 | 7.392 | 3.124 | **Non-CPP** |
| CC_17 | RFGRFLRKIRRFRPKITITIQGSARIG | 27 | 0.0001 | Low | 11.004 | 3245.34 | 9 | 7.414 | 3.149 | **CPP** |
|  |  |  |  |  |  |  |  |  |  |  |
| **OMIGANAN** | **ILRWPWWPWRRK** | **12** | **1.036** | **High** | **11.383** | **1780.33** | **4** | **7.515** | **4.073** | **CPP** |
| OM_14 | IIRIPWWPWRRK | 12 | 1.229 | High | 10.85 | 1707.28 | 4 | 7.529 | 3.974 | **CPP** |
| OM_22 | ILRWPWWPKRRK | 12 | 0.97 | Normal | 15.108 | 1722.29 | 5 | 7.836 | 3.811 | **CPP** |
| OM_34 | ILRIPWWPWRRK | 12 | 0.97 | Normal | 10.958 | 1707.28 | 4 | 7.526 | 3.985 | **CPP** |
| OM_29 | ILRCPWWPWRRK | 12 | 0.876 | Normal | 11.342 | 1697.25 | 4 | 7.445 | 3.788 | **CPP** |
| OM_35 | ILRWPIWPWRRK | 12 | 0.955 | Normal | 10.958 | 1707.28 | 4 | 7.526 | 3.985 | **CPP** |
|  |  |  |  |  |  |  |  |  |  |  |
|  |  |  |  |  |  |  |  |  |  |  |
| **APOC1** | **FSTKTRNWFSEHFKKVKEKLKDTFA** | **25** | **0.077** | **Low** | **13.588** | **3132.03** | **7.5** | **7.049** | **3.023** | **CPP** |
| AP_15 | FSTKTRCCFSEHFKKVKEKLKDTFA | 25 | 0.436 | Normal | 11.98 | 2976.81 | 4.5 | 6.569 | 2.864 | **Non-CPP** |
| AP_18 | FSTKTRNWFSCCFKKVKEKLKDTFA | 25 | 0.43 | Normal | 11.16 | 3025.88 | 4 | 6.501 | 2.967 | **Non-CPP** |
| AP_29 | FSTKTRNWFSIIFKKVKEKLKDTFA | 25 | 0.0001 | Low | 10.792 | 3045.94 | 4 | 6.579 | 3.156 | **Non-CPP** |
| AP_34 | FSTKTRNWFSEHFKKVKEKIKITFA | 25 | 0.0001 | Low | 9.756 | 3068 | 5.5 | 6.772 | 3.186 | **CPP** |
| AP_61 | FSTKTRNWFSECFKKVKEKLKDTFA | 25 | 0.105 | Normal | 10.876 | 3069.92 | 4 | 6.518 | 3.072 | **Non-CPP** |
| AP_65 | FSTKTRNWFSEHFKKVKEKCKDTFA | 25 | 0.131 | Normal | 11.848 | 3093.9 | 4.5 | 6.583 | 3.005 | **Non-CPP** |
| AP_66 | FSTKTRNWFSEHFKKVKEKLKCTFA | 25 | 0.1 | Low | 9.84 | 3091.98 | 5.5 | 6.711 | 3.101 | **Non-CPP** |
|  |  |  |  |  |  |  |  |  |  |  |
| **Mutacin** |  |  |  |  |  |  |  |  |  |  |
| **MU_1** | **NRWWQGVVPTVSYECRMNSWQHVFTCC** | 27 | 0.377 | Normal | 3.215 | 3318.18 | 1.5 | 6.035 | 3.168 | **Non-CPP** |
| MU_2 | KRKWQGVVPTVSYECRMNSWQHVFTCC | 27 | 0.561 | Normal | 5.489 | 3274.21 | 3.5 | 6.338 | 3.04 | **Non-CPP** |
| MU_9 | NRWWQGVVPTVSYKCRKNSWQHVFTCC | 27 | 0.537 | Normal | 5.196 | 3314.22 | 4.5 | 6.424 | 3.115 | **Non-CPP** |
| MU_27 | CRCWQGVVPTVSYECRMNSWQHVFTCC | 27 | 0.763 | Normal | 2.141 | 3224.13 | 1.5 | 5.99 | 3.019 | **Non-CPP** |
| MU_36 | NRWWQGVVPTVSYECRMCSCQHVFTCC | 27 | 0.763 | Normal | 2.141 | 3224.13 | 1.5 | 5.99 | 3.019 | **Non-CPP** |
| MU_49 | NRWWQGVVPTVSYECRMISIQHVFTCC | 27 | 0.354 | Normal | 1.8 | 3244.19 | 1.5 | 6.062 | 3.194 | **Non-CPP** |
|  |  |  |  |  |  |  |  |  |  |  |
| **Ec-Pis2** |  |  |  |  |  |  |  |  |  |  |
| **ec_pis2-original** | **FFFHIIKGLFHAGRMIHGLV** | 20 | 0.0001 | Low | -2.135 | 2341.19 | 3.5 | 6.541 | 3.446 | **Non-CPP** |
| ec_31 | FFFHIIKGLFHAGRMIHGCC | 20 | 0.693 | Normal | -1.845 | 2335.16 | 3.5 | 6.449 | 3.249 | **Non-CPP** |
| ec_53 | PFFHIIKGLFHAGRMIHGLV | 20 | 0.153 | Normal | -1.32 | 2291.13 | 3.5 | 6.582 | 3.327 | **Non-CPP** |
| ec_56 | FFFPIIKGLFHAGRMIHGLV | 20 | 0.0001 | Low | -2.725 | 2301.16 | 3 | 6.476 | 3.416 | **Non-CPP** |
| ec_76 | FIFHIIKGLFHAGRMIHGLV | 20 | 0.004 | Low | -2.01 | 2307.18 | 3.5 | 6.568 | 3.434 | **Non-CPP** |
| ec_78 | FFFIIIKGLFHAGRMIHGLV | 20 | 0.0001 | Low | -3.415 | 2317.21 | 3 | 6.463 | 3.523 | **Non-CPP** |
|  |  |  |  |  |  |  |  |  |  |  |
| **SAAP 148** |  |  |  |  |  |  |  |  |  |  |
| **SP_1** | **LKRVWKRVFKLLKRYWRQLKKPVR** | 24 | 0.205 | Normal | 16.183 | 3226.43 | 11 | 7.871 | 3.595 | **CPP** |
| SP_48 | PKRVWKRVFKLLKRYWRQLKKPVR | 24 | 0.172 | Normal | 16.704 | 3210.38 | 11 | 7.885 | 3.501 | **CPP** |
| SP_49 | LKRVPKRVFKLLKRYWRQLKKPVR | 24 | 0.157 | Normal | 16.546 | 3137.33 | 11 | 7.888 | 3.463 | **CPP** |
| SP_57 | CKRVWKRVFKLLKRYWRQLKKPVR | 24 | 0.232 | Normal | 16.321 | 3216.4 | 11 | 7.833 | 3.491 | **CPP** |
| SP_58 | LKRVCKRVFKLLKRYWRQLKKPVR | 24 | 0.219 | Normal | 16.162 | 3143.35 | 11 | 7.836 | 3.453 | **CPP** |
| SP_70 | LKRVWKRVFKILKRYWRQLKKPVR | 24 | 0.181 | Normal | 16.129 | 3226.43 | 11 | 7.873 | 3.59 | **CPP** |
|  |  |  |  |  |  |  |  |  |  |  |
| **Pleurocidin** |  |  |  |  |  |  |  |  |  |  |
| PL_1 | GWGSFFKKAAHVGKHVGKAALTHYL | 25 | 1.04 | High | 1.04 | 1098.4 | 2 | 6.595 | 2.712 | Non-CPP |
| PL_4 | GWGSFKKKAAKVGKHVGKAALTHYL | 25 | 1.212 | High | 6.27 | 1079.4 | 3 | 7.021 | 2.478 | Non-CPP |
| PL_18 | GWGSFFKKAAHVGKHPGKAAPTHYL | 25 | 1.04 | High | **1.04** | 1098.4 | 2 | 6.595 | 2.712 | Non-CPP |
| PL_13 - | GWGSPPKKAAHVGKHVGKAALTHYL | 25 | 1.222 | High | 4.3 | 998.28 | 2 | 6.759 | 2.234 | Non-CPP |
| PL_61 | GWGSFFKKAAHVGKHVGKAALTHPL | 25 | 1.026 | High | 2.024 | 2645.49 | 5.5 | 6.719 | 2.718 | Non-CPP |
| **PcAst-1a** |  |  |  |  |  |  |  |  |  |  |
| PC_1 - | SNGYRPAYRPAYRPSYRPGK | 20 | 0.396 | Normal | 12.545 | 2356.89 | 5 | 7.067 | 2.72 | CPP |
| PC_6 | SCGCRPAYRPAYRPSYRPGK | 20 | 1.109 | High | 10.555 | 2285.88 | 5 | 7.018 | 2.577 | Non-CPP |
| PC_15 | SNGPRPAYRPAYRPSPRPGK | 20 | 0.713 | Normal | 12.335 | 2224.77 | 5 | 7.131 | 2.519 | Non-CPP |
| PC_19 | SIGCRPAYRPAYRPSCRPGK | 20 | 1.239 | High | 9.76 | 2235.87 | 5 | 7.036 | 2.584 | Non-CPP |
| PC_20 | SNGIRPAYRPAYRPSCRPGK | 20 | 0.789 | Normal | 11.185 | 2246.84 | 5 | 7.054 | 2.614 | Non-CPP |
